# Metaproteome analysis reveals that syntrophy, competition, and phage-host interaction shape microbial communities in biogas plants

**DOI:** 10.1186/s40168-019-0673-y

**Published:** 2019-04-27

**Authors:** R. Heyer, K. Schallert, C. Siewert, F. Kohrs, J. Greve, I. Maus, J. Klang, M. Klocke, M. Heiermann, M. Hoffmann, S. Püttker, M. Calusinska, R. Zoun, G. Saake, D. Benndorf, U. Reichl

**Affiliations:** 1 Bioprocess Engineering, Otto von Guericke University, Universitätsplatz 2, 39106 Magdeburg, Germany; 2 Max Planck Institute for Dynamics of Complex Technical Systems, Bioprocess Engineering, Sandtorstraße 1, 39106 Magdeburg, Germany; 3 Center for Biotechnology (CeBiTec), University Bielefeld, Universitätsstraße 27, 33615 Bielefeld, Germany; 4 Department Bioengineering, Leibniz Institute for Agricultural Engineering and Bioeconomy (ATB), Max-Eyth-Allee 100, 14469 Potsdam, Germany; 5 Department Technology Assessment and Substance Cycles, Leibniz Institute for Agricultural Engineering and Bioeconomy (ATB), Max-Eyth-Allee 100, 14469 Potsdam, Germany; 6 Environmental Research and Innovation (ERIN), Luxembourg Institute of Science and Technology, 41 rue du Brill, L-4422 Belvaux, Luxembourg; 7 Otto von Guericke University, Institute for Databases and Software Engineering, Universitätsplatz 2, 39106 Magdeburg, Germany

**Keywords:** Metaproteomics, Phages, Anaerobic digestion, Anaerobic Digestion Model 1, Phage-host interactions, Microbiomes

## Abstract

**Background:**

In biogas plants, complex microbial communities produce methane and carbon dioxide by anaerobic digestion of biomass. For the characterization of the microbial functional networks, samples of 11 reactors were analyzed using a high-resolution metaproteomics pipeline.

**Results:**

Examined methanogenesis archaeal communities were either mixotrophic or strictly hydrogenotrophic in syntrophy with bacterial acetate oxidizers. Mapping of identified metaproteins with process steps described by the Anaerobic Digestion Model 1 confirmed its main assumptions and also proposed some extensions such as syntrophic acetate oxidation or fermentation of alcohols. Results indicate that the microbial communities were shaped by syntrophy as well as competition and phage-host interactions causing cell lysis. For the families *Bacillaceae*, *Enterobacteriaceae*, and *Clostridiaceae*, the number of phages exceeded up to 20-fold the number of host cells.

**Conclusion:**

Phage-induced cell lysis might slow down the conversion of substrates to biogas, though, it could support the growth of auxotrophic microbes by cycling of nutrients.

**Electronic supplementary material:**

The online version of this article (10.1186/s40168-019-0673-y) contains supplementary material, which is available to authorized users.

## Background

The anaerobic digestion of organic waste and energy crops to biogas consisting of methane (CH_4_) and carbon dioxide (CO_2_) constitutes an important renewable energy source. A multitude of different bacterial and archaeal species catalyze the different degradation steps providing energy for biomass growth.

In agricultural biogas plants (BGPs), biomass conversion into biogas is incomplete. Based on the theoretical gas potential, the conversion of volatile solids (VS) to biogas from particulate organic matter is only about 30–60% [1] indicating that complete utilization of biomass by the microbial community is impeded by so far unknown mechanisms. Missing enzymes for specific biochemical reactions or high generation times of essential microbial species are discussed as an explanation [2]. In order to determine the specific causes of the low biomass degradation efficiency and to develop strategies for increasing biogas yields, detailed knowledge about the abundances and the physiology of main microbial groups in the BGPs is required [3]. Overall, anaerobic conditions in BGPs provide a smaller total energy gain for microorganisms in contrast to aerobic conditions. Furthermore, sequentially fermenting bacteria and archaea divide this energy into little portions close to thermodynamic limits. The major conversion steps carried out by different microbial groups are hydrolysis, acidogenesis, acetogenesis, and methanogenesis. During hydrolysis, extracellular enzymes hydrolyze biopolymers such as cellulose, proteins, and lipids into their respective monomers. In subsequent acidogenesis, these monomers are fermented to volatile organic acids and alcohols, molecular hydrogen (H_2_), and CO_2_. In the following acetogenesis, volatile organic acids and alcohols are fermented to acetate, H_2_, and CO_2_. For the conservation of energy, these secondary fermentation reactions depend on subsequent homoacetogenesis or methanogenesis which both consume H_2_ changing the thermodynamic equilibrium towards its products. Finally, methanogenesis is the production of CH_4_ from acetate (acetoclastic methanogenesis), H_2_, and CO_2_ (hydrogenotrophic methanogenesis) as well as from methylated compounds (methylotrophic methanogenesis) by methanogenic archaea.

So far, the majority of metabolic pathways have been characterized in pure culture experiments concerning the involved enzymes and the thermodynamic conditions [4, 5]. Simplified structured models such as the Anaerobic Digestion Model 1 [6–8] are used for simulations to assist BGP operation. The Anaerobic Digestion Model 1 is able to predict experimental results of biogas production and biogas composition based on multiple steps describing biochemical as well as physicochemical processes and the abundance of main microbial groups. However, the Anaerobic Digestion Model 1 does not cover more complex biological interactions and mechanisms such as the metabolic versatility of individual microorganisms, the functional interchangeability of different microbial taxa, or the competition and syntrophic interactions between bacteria and archaea [4]. In particular, the impact of the presence of certain microorganisms as well as their specific metabolic pathways on the overall process is still poorly understood and not covered by the Anaerobic Digestion Model 1. For example, syntrophic acetate oxidation is the reversed pathway of homoacetogenesis [9]. Depending on the conditions, the thermodynamic equilibrium between CO_2_, H_2_, and acetate is shifted preferring either syntrophic acetate oxidation or homoacetogenesis [10]. Finally, competition may also have a major effect on the taxonomic and functional composition of microbial communities. For example, species of the archaeal family *Methanosaetaceae* possess enzymes with a high acetate affinity and may suppress other acetate-consuming microorganisms [11]. However, competition is not limited to substrates. For example, certain microbial species may kill other species by the expression of bacteriocins, which lyse or inhibit their competitors [12].

Another recent finding is the presence of phages shaping the microbial communities in anaerobic digestion [13, 14]. Replication of phages results in the lysis of host microorganisms and is discussed to cause significant process disturbances due to removal of essential microbial groups [14]. Details about the interaction of phages and the microbial communities in BGPs are rare because phages are difficult to detect due to their small size and low biomass. Furthermore, only a few phage sequences are known, and the dynamics of phage-host interaction were only studied for few bacterial and archaeal species. For example, bacteria and archaea may defend phage attacks by the expression of CRISPR proteins, which snip out phage genes from their own genome [15]. In summary, all these issues impede the understanding of the microbial communities in BGPs and hamper process development and optimization.

Over the last years, various “omics” studies investigated the taxonomic and functional structure of microbial communities in BGPs. These studies focused on individual genes [16–18], transcripts [19, 20], or used approaches such as metagenomics [21–23], metatranscriptomics [24, 25], and metaproteomics [26–30] to assess the complexity of microbial communities. In contrast to metagenomics and metatranscriptomics, the main advantage of metaproteomics is that expressed enzymes can be detected and quantified. This also includes the detection of phages by the identification of phage proteins. This is in contrast to metagenomics and metatranscriptomics that both study only genes but cannot distinguish between the presence of phages and their inactive genes incorporated into host cell genomes.

The aim of our in-depth metaproteomics study was to identify which mechanisms shape the taxonomic and functional composition of microbial communities in BGPs. Eleven BGPs were investigated at two time points using SDS-PAGE for pre-fractionation of proteins and subsequent liquid chromatography (LC) coupled to a high-resolution Orbitrap Elite tandem mass spectrometer (MS/MS). Proteins were identified using the MetaProteomeAnalyzer software [31]. Subsequently, the taxonomic and functional compositions of the microbial communities were analyzed. Mapping of identified metaproteins to the different metabolic pathways confirmed the Anaerobic Digestion Model 1 and revealed some indications for additional metabolites pathways such as syntrophic acetate oxidation and microbial interactions. In particular, the presence of phages and antimicrobial peptides and proteins was detected. Most likely both influence the microbial biomass turnover and are discussed regarding their impact on the microbial community and on the process model.

## Results

### Operation parameters confirm stable operation of biogas plant operation

In this study, seven large-scale BGPs constructed as continuous stirred-tank reactors (CSTR) encompassing a reactor volume range of 1100–3000 m^3^ and three plug-flow reactors (equipped with a secondary CSTR) covering a volume of 270–350 m^3^ (Table 1) were investigated. Additionally, one laboratory scale CSTR with 3-L working volume was included in this study. Nine reactors were operated under mesophilic process conditions (39.7–43.4 °C), while two parallel (plug-flow) reactors were run under thermophilic conditions (52.2–53.4 °C). Biogas production determined by the daily total biogas volume flux of specific BGPs varied between 2342–22,800 m^3^ biogas per day, with the plug-flow reactors typically achieving the highest biogas productivities of up to 24 m^3^ biogas per day and cubic meter fermenter volume. For the latter, the largest amounts of biogas were produced in the secondary CSTRs. The BGPs were operated with a variety of agricultural feedstocks, characterized by a high proportion of maize silage and manure. Organic loading rates (OLRs) ranged between 1.3–6.1 kg volatile solids (VS) per cubic meter fermenter volume and day and hydraulic retention times (HRTs) between 15.1–86.0 days. The plug-flow reactor systems showed the highest OLRs and the shortest HRTs. All monitored BGPs constantly produced biogas containing about 50% (*v*/*v*) CH_4_. In the large-scale BGPs, the total solids (TS) content of the fermenting liquid was approximately 10%. In contrast, the fluid in the laboratory scale reactor only contained about 4% (*m*/*v*) TS. The ratio of total volatile fatty acids to total alkalinity (TVFA/TA) ranged from 0.1 to 0.6, and the pH values ranged from 7.2 to 8.9. The total acid content was below 2 g L^−1^ in most BGPs investigated. The plug-flow reactor systems BGP_05a and BGP_05b showed considerably higher acid contents in the range of 3.6–10.7 g L^−1^ compared to the CSTR systems analyzed. Acetate (average 80% (*m*/*v*)) dominated the determined VFAs, followed by propionate (mean 16.7% (*m*/*v*)), valerate (mean 8.4% (*m*/*v*)), and butyrate (mean 6.5% (*m*/*v*)). The total ammonia nitrogen (TAN) reached values between 1.8–6.2 g L^−1^.

**Table 1:**
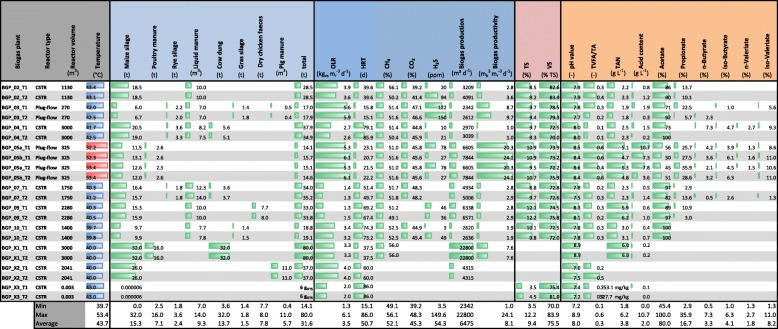
Technical and chemical process parameters of the investigated BGPs

T1 and T2 corresponded to the first and second sampling date. BGP 05 comprised two parallel process lines which were labeled with a and b. Circles indicate the approximate percentage of the substrates, biogas composition or acid composition. Mesophilic process temperature is marked in the column “Temperature” by blue bars and thermophilic process temperature is marked by red bars. Green bars just visualize the values of the different parameters. CSTR: continuously stirred tank reactor; OLR: organic loading rate; HRT: hydraulic retention time; VS: volatile solids; TS: total solids; TVFA/TA: total volatile fatty acids to total alkalinity; TAN: total ammonia nitrogen; m_b_^3^: cubic meter biogas; m_r_: cubic meter reactor volume; kg_vs_: kilogram VS

### Protein extraction and identification enabled comprehensive insight into the microbial communities

SDS-PAGE (Additional file 1: Figure S1) revealed reproducible protein patterns for the technical (separate protein extraction) and biological (different time points) replicates of individual BGPs. Between BGPs, however, protein bands can sometimes differ (for example, BGP05a and BGP04 (Additional file 1: Figure S1C+D). LC-MS/MS measurements of all samples resulted in a total of 14,977,296 MS/MS spectra. Among these spectra, 3,678,352 spectra were identified. The number of identifications per BGP sample varied between 143,423 spectra for the laboratory scale reactor BGP_X3 (lowest number) and 473,462 spectra for BGP_05a (highest number). For removal of redundant hits, protein identifications were grouped into metaproteins using the UniProt Reference Clusters (UniRef) 50 as a grouping criterion [32, 33]. Finally, 16,977 annotated metaproteins were assigned to 181 microbial families and 233 biological processes (UniProtKB Keywords) (Additional file 2: Table S1). However, not all metaproteins could be assigned to a specific order. About 35% of metaproteins were assigned to higher taxonomic level or in worst case to root, only (Fig. 2, Additional file 12).

### Cluster analysis revealed major differences between thermophilic, mesophilic, and lab-scale biogas plants

**Fig. 1:**
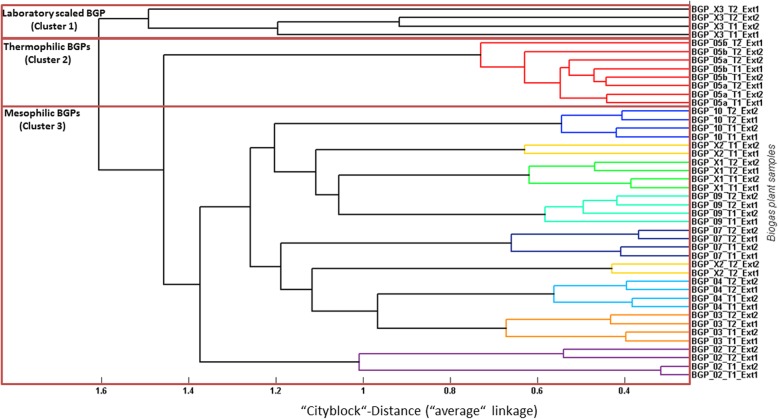
Cluster analysis of all samples based on archaeal and bacterial metaproteins. Cluster analysis was carried out for all metaproteins based on the “cityblock” distance and an “average” linkage using Matlab. All BGPs were colored in a different color. Three main clusters could be observed which were linked with laboratory scaled reactors as well as the process temperature

Reproducibility of the metaproteomic workflow was examined for all samples by hierarchical clustering using “cityblock” distance and “average” linkage based on all metaproteins (Fig. 1, Additional file 3: Note 1). As expected, the highest similarity between metaproteins was observed for technical replicates. Corresponding with the stable process conditions of BGPs, samples taken at different time points were also very similar, except for BGP_X2 which clustered separately. A potential explanation might be an increased TVFA/TA (0.2 for time point 1 and 0.5 for time point 2, Table 1). Overall, three main clusters were found: one for the laboratory scale reactor (BGP_X3), one for thermophilic BGPs (BGP_05a and BGP_05b), and one for mesophilic BGPs (BGP_2, BGP_3, BGP_04, BGP_07, BGP_09, BGP_10, BGP_X1, BGP_X2).

### Metaproteome analysis revealed insight into the major microbial taxonomies and functions

**Fig. 2:**
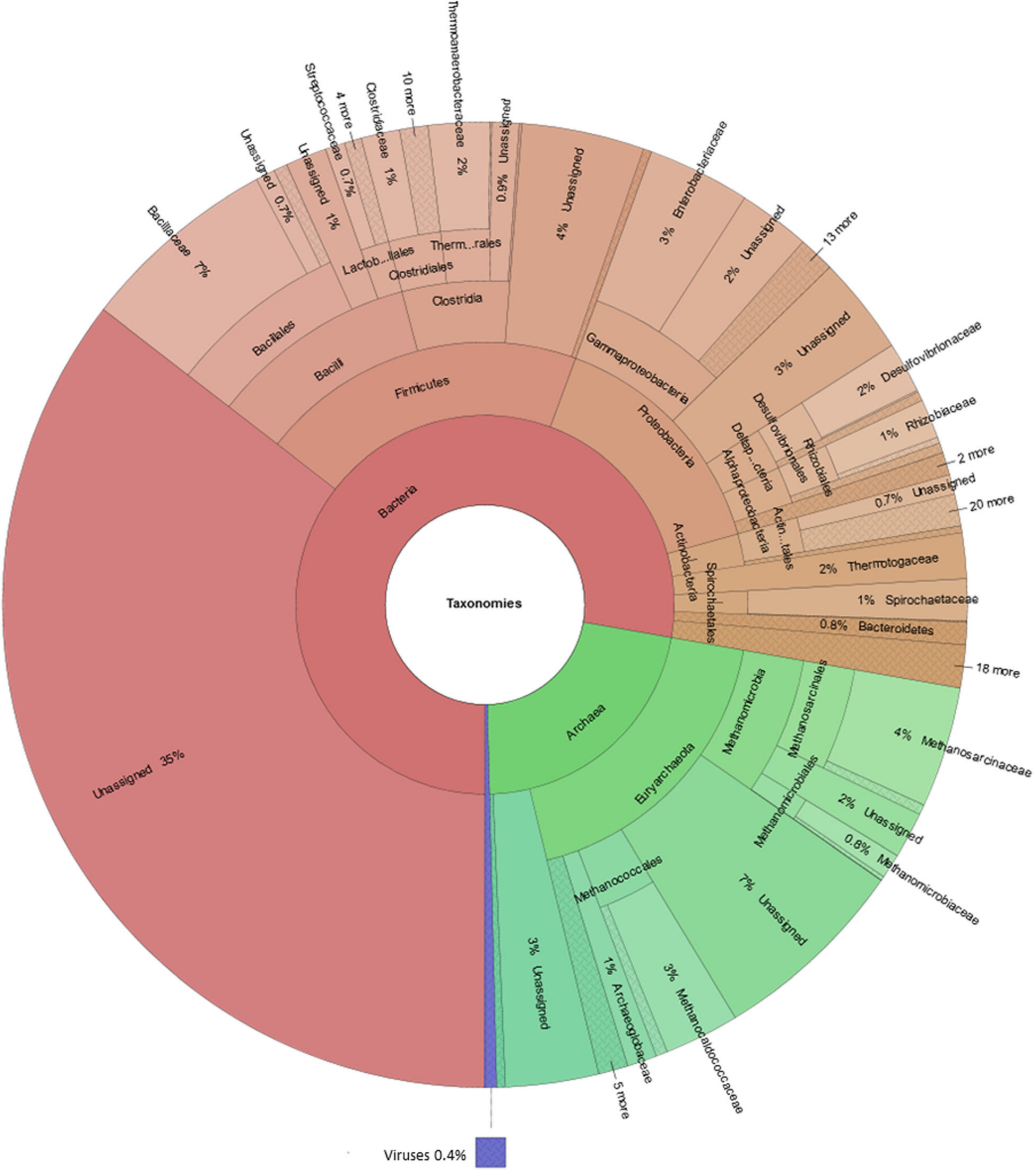
Krona plot of identified bacteria, archaea and viruses. The krona plot shows all taxonomic levels based on the NCBI taxonomy starting from superkingdom to family level and the *associated* abundances based on the number of identified spectra summed over all BGPs. Therefore, all 562,390 identified microbial and viral spectra from all 10,970 metaproteins were fed into the krona plot. For more details please refer to the Additional file 4 “C_InputKronaPlot”. In contrast, the calculation of the phage abundance in Additional file 7: Table S5 considers also metaproteins that were assigned on root level, only. These metaprotein were assigned to phages based on their function. An interactive version of this Figure can be found in Additional file 12

As a first overview about the microbial community structure and the metabolic functionality of the sampled BGPs, the identified microbial families and biological processes are summarized for all analyzed BGPs in Fig. 2, Additional file 12. On average, and based on the spectral abundance, the microbial communities consist of 77.8% ± 30.7% bacteria (minimum 62.60%, maximum 93.58%), 21.9% archaea ± 13.1% (minimum 6.23%, maximum 37.13%), and 0.4% ± 0.3 viruses (minimum 0.11%, maximum 1.21%)*.* Dominant bacterial families were *Bacillaceae* (6.7% ± 2.9%, minimum 2.49%, maximum 9.33%), *Enterobacteriaceae* (3.4% ± 2.0%, minimum 1.39%, maximum 19.50%), *Thermoanaerobacteraceae* (2.1% ± 2.1%, minimum 0.00%, maximum 9.78%), and *Thermotogaceae* (1.5% ± 3.0%, minimum 0.07%, maximum 5.94%). In the samples of the thermophilic BGPs (BGP_05a and BGP_05b), higher amounts of bacterial families associated with thermophilic conditions (e.g., *Thermotogaceae*) were found. Dominant archaeal families were *Methanosarcinaceae* (4.1% ± 3.7%, minimum 0.42%, maximum 9.57%), *Methanocaldococcaceae* (2.5% ± 1.4%, minimum 0.79%, maximum 4.12%), and *Archaeoglobaceae* (1.0% ± 0.5%, minimum 0.30%, maximum 2.19%).

The main biological processes in BGPs, as identified by their UniProtKB Keyword, were “Transport” (18.8% ± 6.2%, minimum 13.86%, maximum 31.46%), “Methanogenesis” (8.5% ± 9.1%, minimum 1.81%, maximum 29.49%), “One-carbon metabolism” (4.9% ± 3.0%, minimum 1.35%, maximum 10.79%), “Carbohydrate metabolism” (4.2% ± 2.0%, minimum 0.92%, maximum 13.42%), and “Sugar transport” (4% ± 1.7%, minimum 1.94%, maximum 10.99%). A more detailed overview of identified taxa and biological processes is given in Additional file 2: Table S1: Worksheet S3 and S4.

**Fig. 3:**
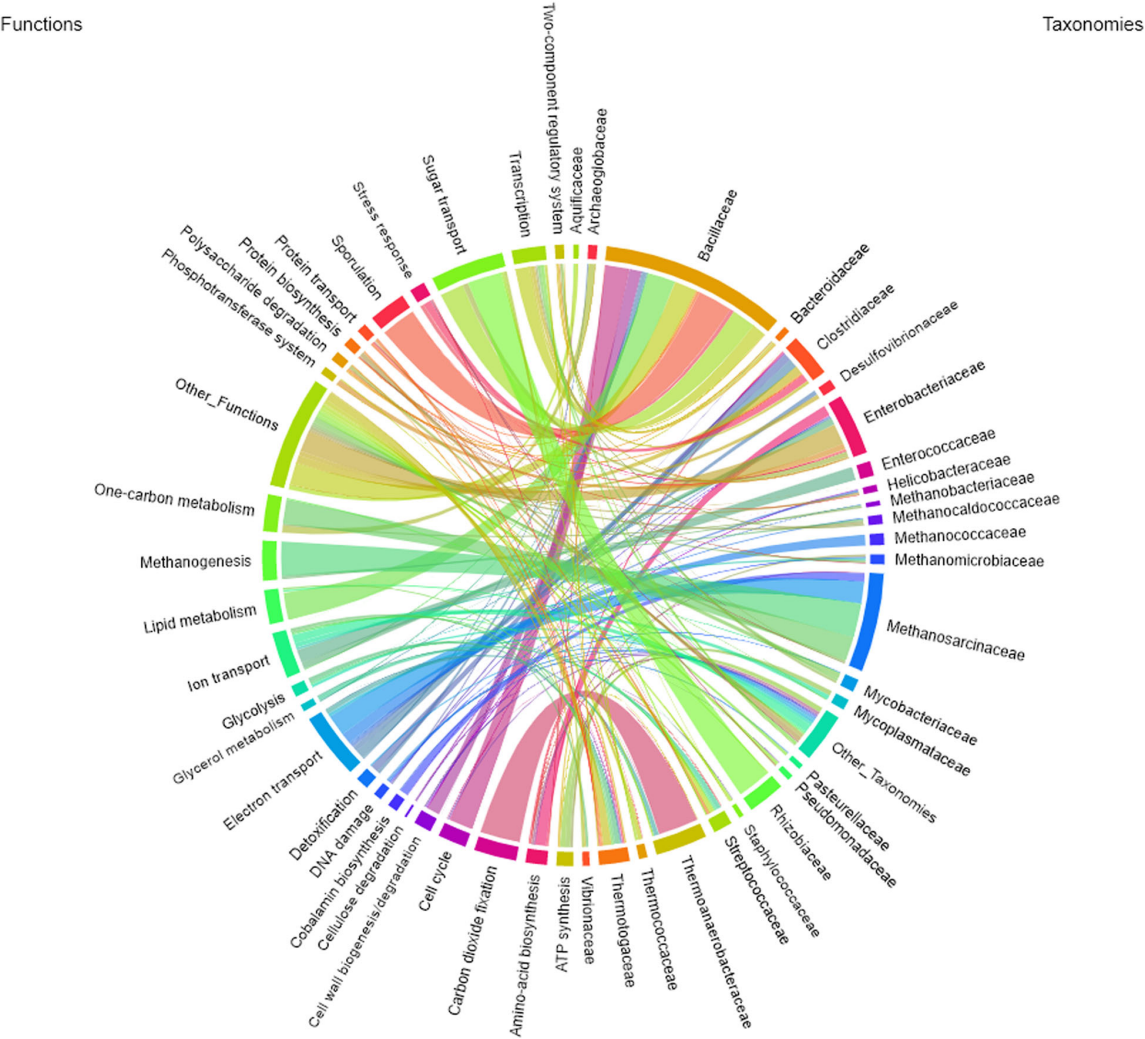
Linkage between taxa and functions. The chord diagram shows the link between taxonomic families and biological processes for the 20 most abundant taxonomic families and 20 most abundant biological processes based on the number of spectral counts summed for all BGPs. The size of a circle segment corresponds to the spectral abundance of a taxon or biological process, while the arcs connecting them correspond to the amount of spectra shared by two entities. The data were exported directly from the MetaProteomeAnalyzer and are stored in Additional file 4: Table S2. In contrast to the print version of this figure, the interactive plot enables to visualize and select all families and biological processes. An interactive version of this Figure can be found in Additional file 13

In order to link metaprotein taxonomies with their respective functions, a chord diagram (Fig. 3, Additional file 13, Additional file 4: Table S2) was created using the NCBI taxonomic families [34] and the UniProtKB keywords of the category “Biological Process”. In accordance with the krona plot (Fig. 2, Additional file 12), *Bacillaceae* was the most dominant family among all classified families. A high number of metaproteins assigned to this family were linked to multiple functions associated with degradation of biomass including sugar transport, carbohydrate metabolism, and lipid metabolism. Furthermore, members of the family *Bacillaceae* expressed large amounts of metaproteins for transcription and sporulation.

The high abundance of sugar transporters assigned to *Rhizobiaceae* and *Bacillaceae* indicates either competition, functional redundancy, or functional differentiation. Whereas the alpha-glucosides-binding periplasmic protein of *Rhizobiaceae* was highly abundant in all BGP, the probable arabinose-binding protein of *Bacillaceae* was detected in several BGP, only. The presence of the arabinose-specific transporter is related to hemicellulose degradation releasing arabinose could hint for functional differentiation of BGP. Cellulose degradation was linked based on the identified metaproteins to the families *Clostridiaceae* and *Thermotogaceae* using an interactive version of the chord diagram (Fig. 3, Additional file 13, Additional file 4: Table S2).

The family *Methanosarcinaceae* dominated methanogenesis in the chord diagram but not completely in the krona plot. The obvious discrepancy between the taxonomic composition of methanogenic archaea in the krona plot and the chord diagram is caused by the fact that many metaproteins could not be assigned to a family rank. For example, the protein V-type ATP synthase subunit C (UniRef50_A0B9K4) was assigned only to the order *Methanosarcinales* but not to a specific family. Low abundant biological processes (“Other_Functions”) were mainly assigned to well-characterized families such as *Bacillaceae* (e.g., “Aromatic hydrocarbons catabolism”, “Cell shape”, “Germination”) and *Enterobacteriaceae* (“DNA condensation”, “Lipopolysaccharide biosynthesis”, “Purine metabolism”). Probably, this is reasoned by the fact that these families comprise well-studied microorganisms such as *Escherichia coli* and *Bacillus subtilis*, for which the proteins are well annotated.

### Detailed assignment of metaproteins regarding their role in anaerobic digestion

The strength of metaproteomics is that individual metaproteins can be quantified and mapped to actually occurring pathways in anaerobic digestion. Therefore, detailed assignments of metaproteins to hydrolysis (Additional file 5: Table S3 A_Hydrolysis) and substrate uptake (Additional file 5: Table S3 B_Substrate_Uptake), fermentation pathways (Additional file 5: Table S3 C_Fermentation), amino acid metabolism (Additional file 5: Table S3 D_AA Metabolism), and CH_4_ production through methanogenesis (Additional file 5: Table S3 E_Methanogenese) were made.

All identified metaproteins were mapped to the Anaerobic Digestion Model 1 [6–8] in order to summarize the results which were presented and discussed in detail in Additional file 3: Note 1. Most of the process steps of the Anaerobic Digestion Model 1 were covered by the identified metaproteins, i.e., biomass degradation to CH_4_ and CO_2_. However, no evidence for lipid degradation and valerate fermentation were found in the investigated agricultural BGPs as shown by the absence of enzymes for hydrolysis and uptake of lipids resp. its degradation products. In contrast, enzymes for hydrolysis and uptake of proteins/peptides and carbohydrates were found in high abundance. Amino acids were subsequently deaminated to ammonia and short-chain fatty acids. For example, glycine was deaminated by both glycine reductase and the glycine cleavage system in order to balance the redox potential (Additional file 3: Note 1) [35].

The identification of large amounts of metaproteins involved in alcohol (24.76% ± 19.89% of the enzymes assigned to fermentation; minimum 2.02%, maximum 54.30%) and lactate fermentation (5.74% ± 3.79% of the enzymes assigned to fermentation; minimum 0.01%, maximum 13.85%) (Additional file 5: Table S3 C_Fermentation) suggest that both pathways play a central role in BGPs. Interestingly, the corresponding process steps are not covered by the Anaerobic Digestion Model 1.

**Fig. 4:**
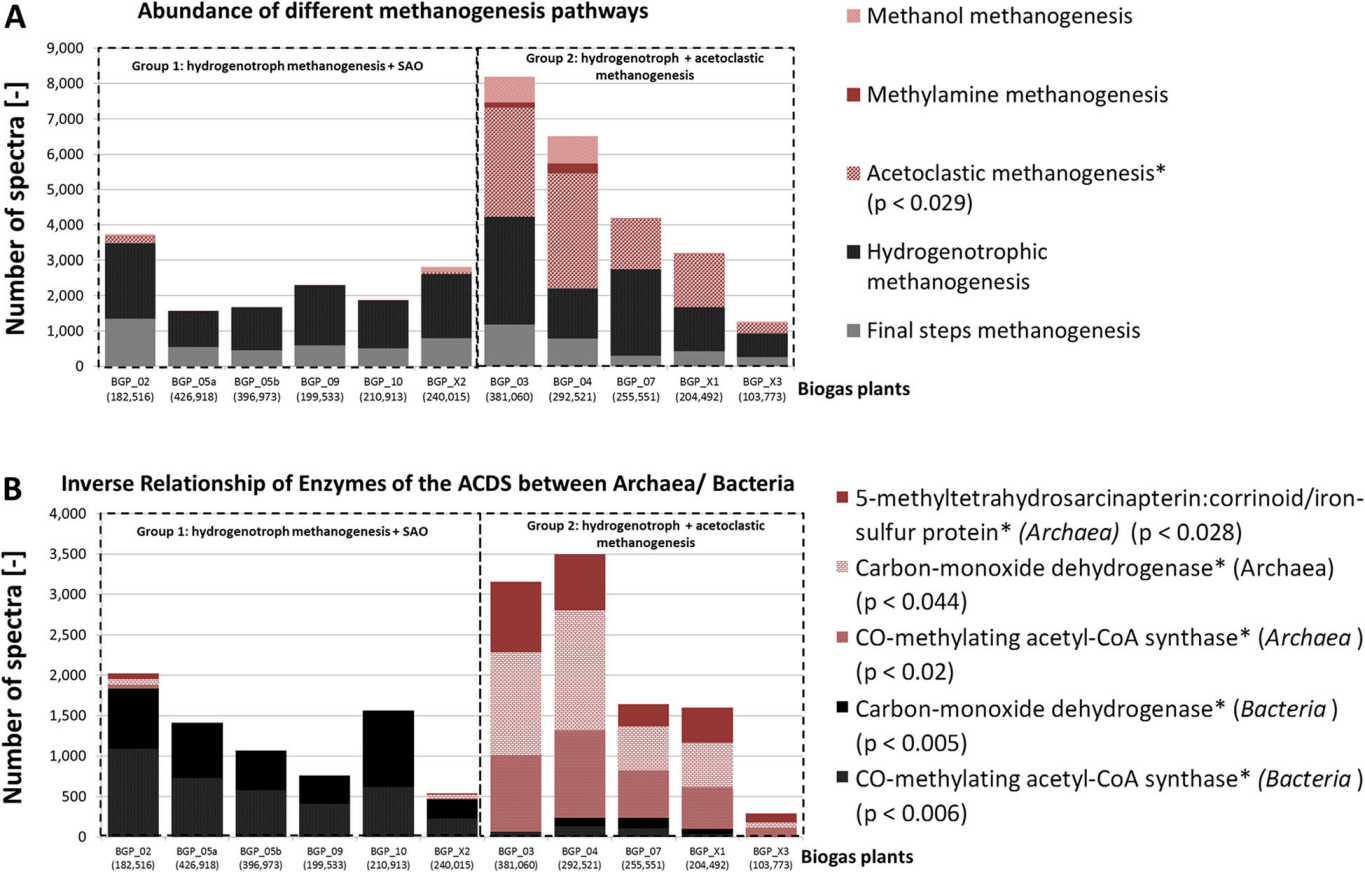
Abundance of methanogenesis pathways as well as of archaeal and bacterial acetyl-CoA decarbonylase/synthase (ACDS). Spectral counts of representative metaproteins for A.) methanogenesis pathway and B.) each ACDS metaprotein (Additional file 5: Table S3 E_Methanogenese) sorted by archaeal and non-archaeal and summed. The black bars indicate bacterial one carbon metabolism and hydrogenotrophic methanogenesis. The red bars are associated with either acetoclastic methanogenesis or acetoclastic methanogenesis as well as the methanol and methylamine pathways. Differences between both groups of BGPs were validated by student’s t-test and highlighted by “*” and the associated p-values. The parentheses under the sample names on the x-axis show the total number of identified microbial spectra for each BGP

In the analyzed BGPs, methanogenesis was carried out either by a combination of hydrogenotrophic and acetoclastic (i.e., mixotrophic) methanogens or, exclusively, by strictly hydrogenotrophic methanogens. In the latter case, large amounts of the bacterial acetyl-CoA decarbonylase/synthase (ACDS) protein complex (Fig. 4) were present to replace the archaeal ACDS, which belongs to the acetoclastic methanogenesis. Furthermore, evidence of phages, antimicrobial peptides as well as proteins, and proteins involved in the microbial immune defense were found (Additional file 6: Table S4 and Additional file 7: Table S5). This implied differing mechanism of competition and killing of individual microbial groups. This issue is also neglected in the Anaerobic Digestion Model 1 but will be considered in more detail in the following paragraph.

### Fate of microbial biomass

The fate of microbial biomass was investigated, since the balance between microbial death and re-growth may affect the anaerobic digestion process and the biogas production. There are three principal reasons for microbial death: (1) microorganisms die due to unfavorable environmental conditions, (2) microorganisms are lysed by phages (Additional file 7: Table S5), and (3) microorganisms are killed by other microorganisms (e.g., directly by predatory bacteria or mediated by secretion of antimicrobial peptides and proteins) (Additional file 8: Table S6).

**Fig. 5:**
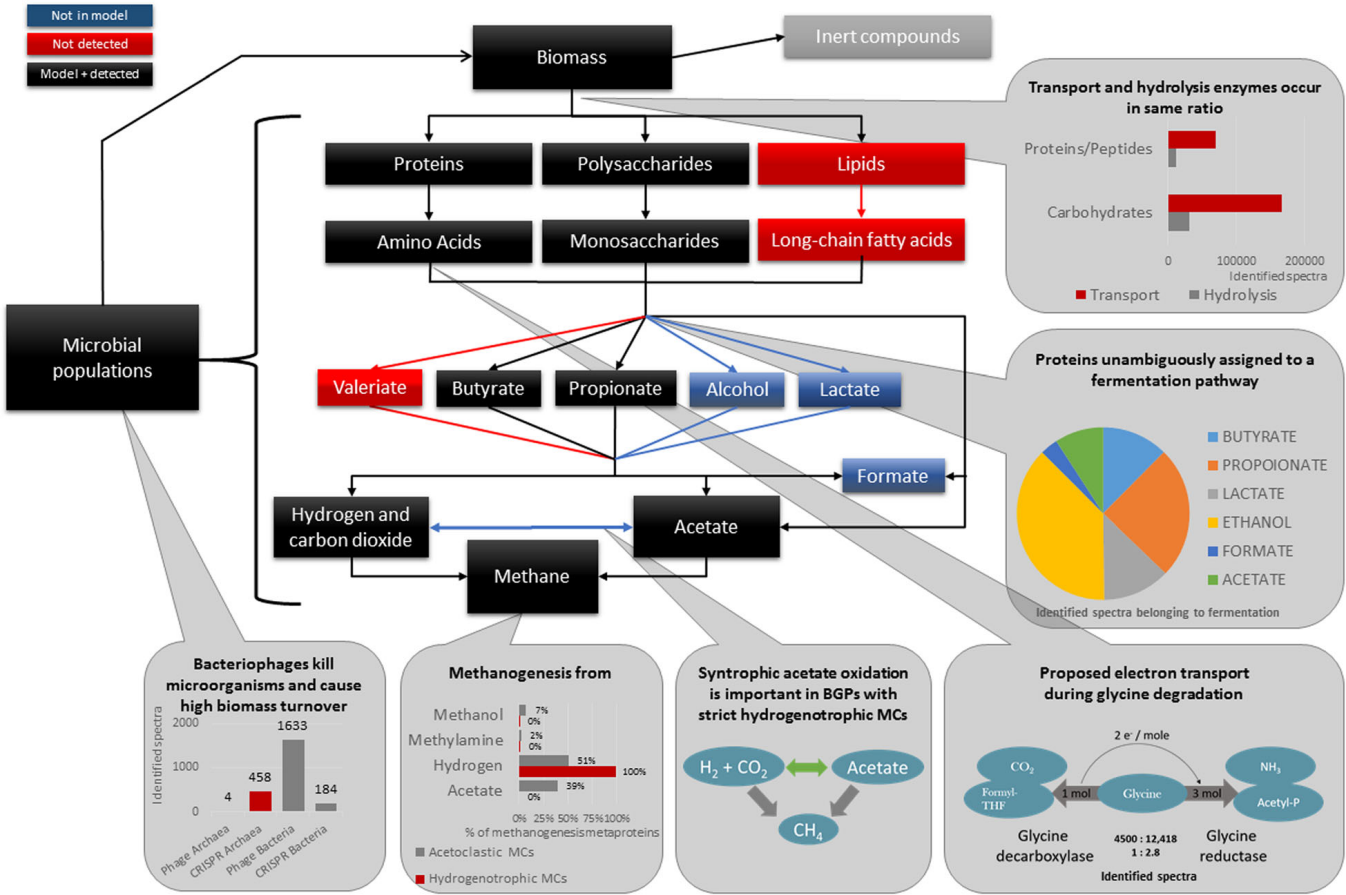
Mapping of the identified metaproteins to the Anaerobic Digestion Model 1. Identified metaproteins were assigned to the single steps of the Anaerobic Digestion Model 1. Significant differences between the assumed steps in the Anaerobic Digestion Model 1 and the proved steps by the identified metaproteins were highlighted in RED or BLUE color. Aspects that were not covered by metaproteomics analysis are displayed in gray (e.g., “Inert compounds”). For each of the analyzed steps a summary provides the most important findings of this study. MCs: microbial communities

Overall 0.4% ± 0.3% (minimum 0.11%, maximum 1.21%) of the identified spectra were associated with viral proteins (Fig. 2, Additional file 12). The highest virus abundance was observed for the thermophilic BGPs, i.e., BGP_05a and BGP_05b (Fig. 5 and Additional file 7: Table S5). In contrast to Fig. 2, Additional file 12, the calculation of the phage abundance in Fig. 5 and Additional file 7: Table S5 considers also phage metaproteins that were assigned automatically on root level, only (Additional file 9: Figure S2). The manual reannotation of this large group accounting 77% of all identified viral spectra was carried out using descriptions of metaproteins indicating typical viral functions. Furthermore, phage metagenome sequences from BGPs [13] were added to the reference database. But the number of identified phage proteins did not increase (data not shown). A large portion of phage proteins was identified based on single peptides matching from conserved domains. In future experiments, the identification of phage proteins has to be improved by better matching phage metagenomes.

The largest abundance of viral metaproteins was found for the orders *Caudovirales* (12.19% ± 14.95%, minimum 0.66%, maximum 58.72%), with its major families *Myoviridae* (3.78% ± 5.80%, minimum 0.00%, maximum 16.95%), *Siphoviridae* (7.15% ± 10.00%, minimum 0.00%, maximum 52.19%), and *Podoviridae* (1.26% ± 1.70%, minimum 0.00%, maximum 9.02%) (Additional file 6: Table S4). Some viral proteins were identified for plant and animal pathogens (e.g., *Rice dwarf virus*). Furthermore, 1.67% ± 2.74% of viral proteins were classified as prophage proteins encoded in the microbial genomes. Since only *viruses* targeting archaea and bacteria are important for the microbial communities we focused on this subgroup termed phage.

Most phage proteins were functionally annotated as capsid proteins (25.7% ± 61.1%, minimum 0.00%, maximum 82.19%) followed by structural proteins (12.9% ± 51.5%, minimum 0.00%, maximum 52.75%) and tail proteins (12.8% ± 38.8%, minimum 0.00%, maximum 66.67%) (Additional file 6: Table S4 and Additional file 10: Figure S3). Furthermore, several proteins required for phage replication such as terminase (1.2% ± 3.5%, minimum 0.00%, maximum 11.11%) or polymerase (3.4% ± 9.0%, minimum 0.00%, maximum 43.33%) were found. In the following, the possible impact of phages on the taxonomic composition of the microbial communities in the BGPs is investigated. First, major phage groups were classified by their host family and compared with the abundance of the major microbial families (Fig. 5, Additional file 7: Table S5). Second, the abundance of metaproteins related to the microbial immune response was calculated based on the detected number of spectra for CRISPR metaproteins (Fig. 5, Additional file 7: Table S5).

Out of 562,390 identified microbial and phage spectra (100%), 9340 (1.6% of the microbial community) spectra were assigned to phages and 3253 (0.6% of the microbial community) to CRISPR metaproteins (Additional file 7: Table S5). In contrast to the low phage abundance suggested in the krona plot (0.4%), phage abundance based on the spectral count is most likely significantly higher when taxonomical unassigned or to the host-assigned phage proteins (prophage proteins) are taken into account. This concerns for example major capsid proteins (UniRef50_B2ZYY5) which were not assigned to any taxonomy.

Furthermore, the presence and abundance of CRISPR and phage metaproteins varied for individual families and samples. In contrast to the high abundance of bacterial phages (i.e., 1.2% of the considered bacterial families), almost no archaeal phages (i.e., 0.1% of the considered families) were found (Fig. 5, Additional file 7: Table S5) (*p* value < 0.0042).

Main targets of phages were the bacterial families *Bacillaceae* (0.7% related to the abundance of this family), *Enterobacteriaceae* (2.8% related to the abundance of this family), and *Clostridiaceae* (2.3% related to the abundance of this family). In contrast to the abundance of phage metaproteins, the average abundance of CRISPR metaproteins related to the abundance of the considered families was 0.1% for bacterial and 0.8% for archaeal families, respectively. Among the methanogenic archaea, it was notable that only CRISPR metaproteins for the family *Methanocococaldacaea* (1.9% related to the abundance of this family) were observed*.*

Microorganisms can impede the growth or even kill other microorganisms coexisting in the same environment by secreting antimicrobial peptides or proteins [12]. Throughout all BGP samples, different antimicrobial peptides and proteins accounting to 0.1% of all spectra (2.907 spectra) were identified (Additional file 8: Table S6). In particular, large amounts of linocin, lysozyme, and maritimacin were found, with maritimacin being enriched in the thermophilic BGPs, namely in BGPs BGP_05a + b (*p* value < 0.00004). For the latter, no specific target organisms are reported [36]. Finally, it has to be taken into account that most taxonomic assignments of antimicrobial peptides and proteins only refer to a specific superkingdom hampering a deeper analysis of its origin. Nevertheless, their relatively high abundance under thermophilic conditions could be evidence for stronger competition under this process regime.

## Discussion

This study examined the microbial functional networks of ten agricultural BGPs and one laboratory scale biogas fermenter using a comprehensive, high-resolution metaproteomics approach. Additional pre-fractionation increased the number of identified metaproteins up to ten times in comparison to a previous study [29] and enabled a more detailed description of individual metabolic pathways in biogas production. Hierarchical clustering demonstrated the reproducibility of the metaproteomics workflow, as exemplified in Fig. 1, where first technical replicates and then samples for different time points grouped together.

Similar to earlier studies on BGPs [29], proteins were grouped into metaproteins based on homologous protein clusters (Uniref50) using MetaProteomeAnalyzer software. The taxonomic affiliations of determined metaproteins were defined as common ancestor taxonomies from all identified peptides belonging to proteins from the same UniRef50. As a result, specific taxonomic ranks could not be assigned to all metaproteins (e.g., 35% of bacteria were left unassigned taxonomy in the krona plot (Fig. 2, Additional file 12)). Grouping to UniProt Reference Clusters (UniRef) 50 is very stringent in comparison to other strategies, e.g., shared peptides. The lower number of resulting metaproteins was considered to be more beneficial to compare the samples of this comprehensive dataset. The selection of a grouping strategy is critical and should be decided considering several criteria, e.g., the size of the experiment and the focus of the experiment (focus on taxonomic or functional level). Specificity of taxonomies could be increased when processing the metagenome data to individual genome bins [37, 38].

### Assignment of metaproteins regarding their role in anaerobic digestion process

**Fig. 6:**
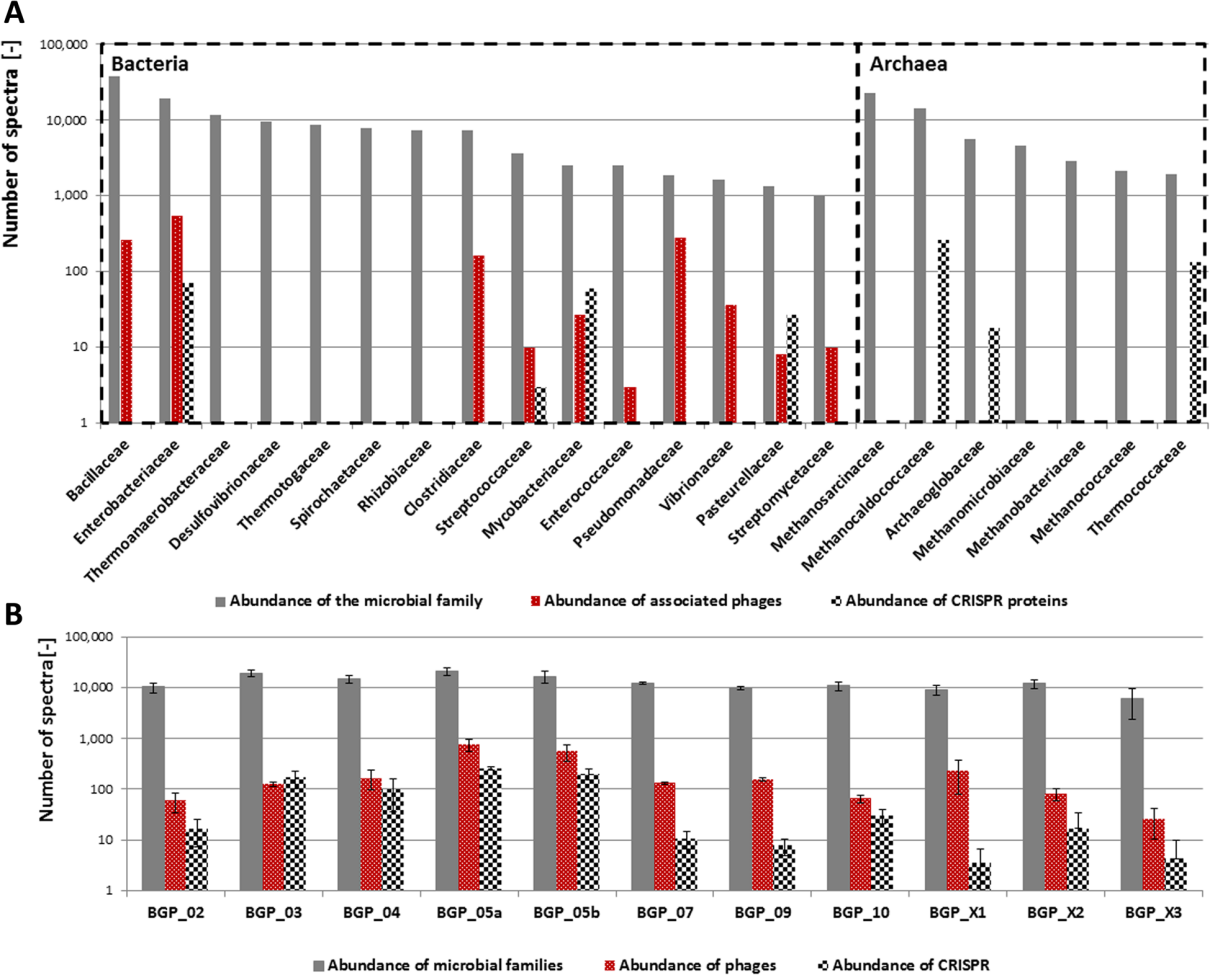
Abundances of microbial families, phages and CRISPR proteins. Figure A shows the main microbial families (at least 1000 spectra for each family) and their associated phages or CRISPR proteins based on the spectral count. Figure B shows the abundance of the microbial families, phages and CRISPR proteins for each biogas plant

The results of this study confirmed the taxonomic and functional composition obtained in previous metaproteome studies [26, 27, 29, 39, 40]. Furthermore, the assignment of the metaproteins to the different metabolic pathway as of the Anaerobic Digestion Model 1 fitted rather well. However, our results suggest that some biological processes are not or only poorly represented by this model (Fig. 6). For example, lactate fermentation is most likely taking place in BGPs as large amounts of lactate are produced during the ensiling process for conservation and storage of crop material as primary or co-substrate for the anaerobic digestion process.

The analysis of archaeal metaproteins showed that hydrogenotrophic methanogenesis was universal for all BGPs, with some microbial communities in BGPs strictly following this pathway. Acetoclastic methanogenesis was found to dominate microbial communities in five of 11 BGPs suggesting two groups of BGPs. The first group of BGPs (acetoclastic and hydrogenotrophic methanogenesis) contained only trace amounts of metaproteins linked to the bacterial C1 metabolism. The second group (strictly hydrogenotrophic BGPs) showed a high abundance of proteins related to the bacterial C1 metabolism enabling the interconversion of acetate to H_2_ and CO_2_. Due to the fact that proteins are missing for acetoclastic methanogenesis as a sink for acetate, the most likely metabolic flow is syntrophic acetate oxidation [10], which might be considered for extension of the Anaerobic Digestion Model 1. However, the presence of syntrophic acetate oxidation in the second group did not correlate to any of the considered process conditions (data not shown). Accordingly, the presence of two different types of microbial communities might also be influenced by a combination of parameters such as temperature, metabolite concentrations, and inoculum that could not be identified due to the still limited number of samples analyzed.

### Influence of syntrophy, competition, and phage-host interaction on anaerobic digestion process

Microbial communities in BGPs consist of a mixture of fermentative bacteria and methanogenic archaea. Certain microbial species depend on syntrophic interaction [4] where two different microbial species metabolize a certain substrate together, as shown for syntrophic acetate oxidation and hydrogenotrophic methanogenesis [10]. Furthermore, the present study showed that different microbial species are competing for substrates and that the microbial communities might also be shaped by phages. Apparently, *Rhizobiaceae* and *Bacillaceae* expressed high amounts of sugar transporters. Their growth is dependent on sugars released by cellulolytic *Thermotogaceae*, *Clostridiaceae*, and *Cellulomonadaceae* (Additional file 3: Note 1). Instead of expressing their own enzymes for hydrolyzing cellulose, they were cheating on monomeric sugars released by the enzymes of the cellulolytic microbes. Cheating could be considered somehow as competition and is preferred in less structured environments like mixed BGPs [41]. The detection of antimicrobial peptides and proteins such as lysozyme and maritimacin impeding the growth or killing potential competitors [12] suggests that biological warfare might play a crucial role in community composition and even nutrient turnover of BGPs. The highest concentrations of antimicrobial peptides and proteins as well as phages were observed in thermophilic BGPs, which have been shown to be less stable in operation [42]. Very likely, the presence of both can lead to stress of the microbial community and may contribute to process instabilities.

**Fig. 7:**
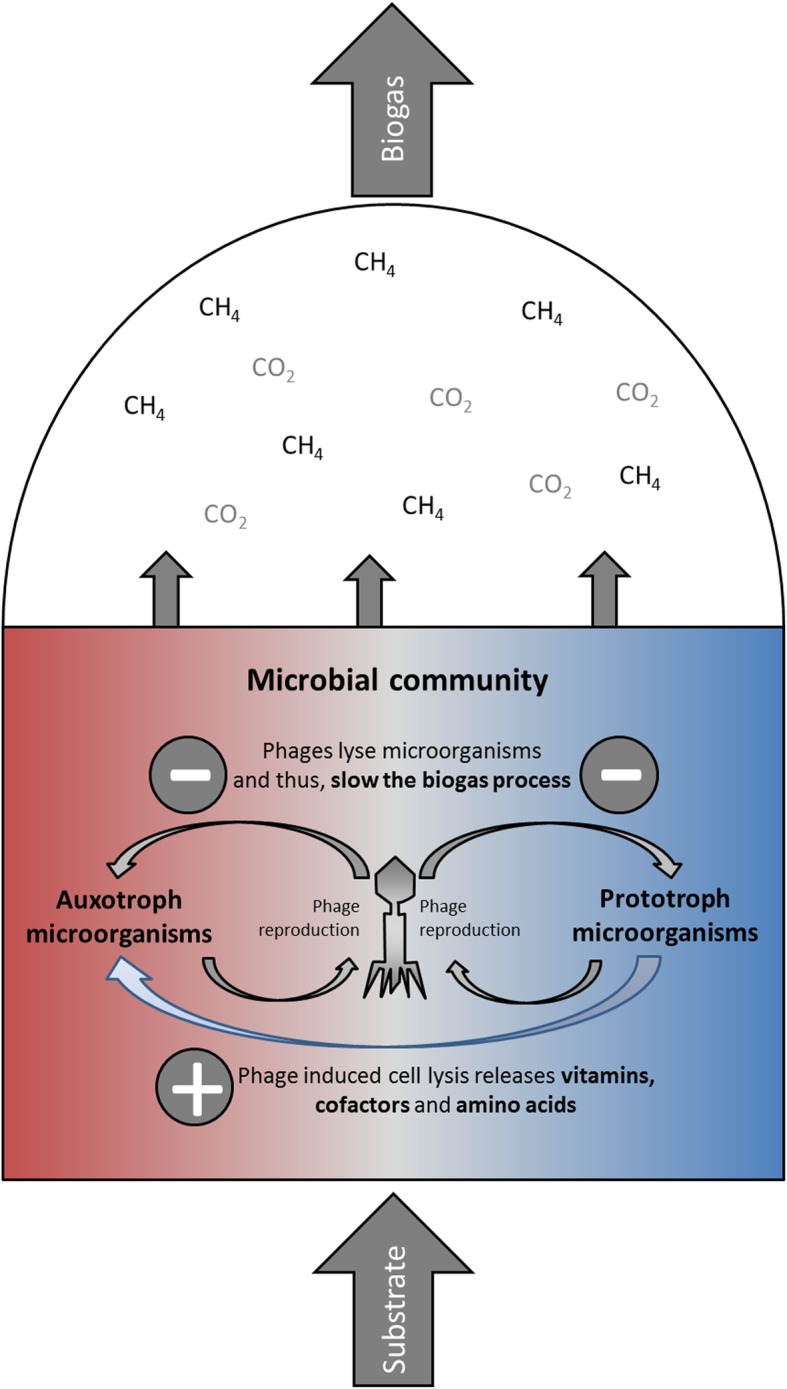
Impact of phages on biogas processes and on the nutrition cycle in biogas plants. The microbial community consists of auxotroph microorganisms and prototroph microorganisms. Whereas prototroph microorganisms may produce vitamins, cofactors and amino acids for their growth themselves, auxotroph microorganisms require external sources for these compounds. Phage induced cell lysis of both microbial groups slows biogas processes due to the lyses of the microorganisms. However, it represents also a major source of vitamins, cofactors and amino acids for the auxotroph microorganism

The presence of both phage proteins and microbial phage defense proteins belonging to the CRISPR system in all analyzed BGPs adds another level of competition. Taking into account the small number of sequenced phages, many phage proteins were likely not identified due to the lack of primary sequence data. Accordingly, the scarcity of sequence data also limited the detailed taxonomic assignment of phages to their hosts. Since the use of a phage-specific metagenome from other BGPs [13] did not increase the number of identified phage proteins, phages in BGPs are probably much more diverse than expected. In the BGPs studied here, *Caudovirales* constituted the largest order of phages as shown previously [13, 14]. At first glance, the average abundance of viral proteins appears to be low. However, taking into account the size of phages in comparison to microbial cells, this perspective changes drastically. Assuming spherical shapes, similar protein amounts of phages and microorganisms, a mean phage diameter of 100 nm and a mean cell diameter of 1.0 μm, and a mean abundance of viral proteins of 0.4% in BGPs correspond roughly to four phages per cell (Additional file 11: Note 2). Potentially, the actual amount of phages is even higher since Kleiner et al. [43] observed for a synthetic mock community an underrepresentation of phages by metaproteome analysis. This indeed is in the range of expected phage particles per cell in other ecosystems [44]. Moreover, phage metaproteins specifically targeting *Clostridiaceae* and *Enterobacteriaceae* amounted to 2.3–2.8% of the bacterial protein (Additional file 7: Table S5) corresponding to a phage load of approximately 20–30 phages per cell. Whereas the high abundance of phage and CRISPR metaproteins for *Enterobacteriaceae* might be explained by a higher rate of identification due to a variety of studies and associated protein entries in databases [45, 46], the high phage abundance of *Clostridiaceae* and *Bacillaceae* might be related to specific biological processes. In case phage abundance corresponds to a decrease in the number of main cellulose degraders belonging to the family *Clostridiaceae*, hydrolysis of complex polymers and thus anaerobic digestion could be negatively influenced (Fig. 7).

Due to the difficulties in the detection of phage proteins and the assignment of phages to specific hosts, it still remains unclear whether phage-induced cell lysis is a rare and transient event or has a significant impact on the composition of BGP communities and the dynamics of biomass conversion. On the one hand, the extent to which phages induce a re-routing of certain degradation pathways of biomass by killing key players of the microbial community has to be clarified in further experiments. On the other hand, lysis of selected bacterial populations must not necessarily be considered as a primarily negative effect on biogas production. In particular, the lysis of infected cells results in a release of highly complex intracellular compounds including vitamins, carbohydrates, and amino acids which can support the growth of other members of the community (Fig. 7). Whereas prototroph microorganisms may produce these compounds on their own, auxotroph microorganisms depend on such external sources [47]. The presence of transporters for vitamins, cofactors, and trace elements (Additional file 3: Note 1) detected in this study supports this hypothesis. This is also in accordance with results obtained for other habitats, i.e., marine or animal microbiomes [45]. Furthermore, pure cultures of secondary fermenters and methanogens are known to require the addition of complex substrates such as sludge fluid, rumen fluid, and yeast extract for growth [48].

Unlike phages targeting *Clostridiaceae*, the appearance of phages specific for *Bacillaceae* correlated with high expression of sporulation proteins, e.g., stage II sporulation protein D (UniRef50_P07372) and stage V sporulation protein T (UniRef50_P37554). Sporulation of *Bacillaceae* could be an option to escape from phage infection, because the replication of the phage genome is inhibited in sporulating cells [49]. The mechanism for preventing phage infection is stochastically trapping the phage DNA in only 20% of the spores [49] due to the reduction of the cell volume. However, certain phages such as phage φE might integrate their DNA very efficiently in the spore, providing pseudolysogeny [49]. In this case, phage DNA is stabilized in the endospore against hazardous environmental conditions, enabling a long-term survival of the phages. Upon germination and growth of vegetative cells, the virulence is activated. The co-occurrence of sporulation and phage proteins of *Bacillaceae* indicates the ongoing arms race between bacteria and phages.

Considering other bacterial families, the phage load was lower. For example, no phages were detected for the high abundant families *Thermoanaerobacteraceae* and *Desulfovibrionaceae*. However, the lack of phages for certain families could be also caused by the failure to assign more than 79.7% of viral proteins to their hosts (Additional file 5: Table S3).

In contrast to bacteria, only few phages were detected that target archaea. This is in accordance with the low number of phages known to date infecting methanogens [13]. In addition, the high expression level of antiviral defense metaproteins (i.e., CRISPR) in several of the archaeal families, e.g., *Methanococcaceae*, might play a role*.*

The results point out the presence of phages as factors shaping microbial communities in BGPs. Whether phage-induced cell lysis slows down the biogas processes or supports the growth of auxotrophic microbes in the biogas processes by nutrient cycling needs further clarification. The best confirmation of these results would be through the isolation and description of phages and corresponding hosts allowing experiments in well-defined systems. Enrichment and sequencing more phage metagenomes from BGPs [13] as well as annotating prophage sequences from genomes could improve the assignment of phage proteins to their hosts [50, 51]. Furthermore, the abundance of phages should be correlated to process conditions, if possible to process disturbances. For example, foaming in BGPs could be related to phage-induced cell lysis releasing proteins that stabilize foam. Metaproteomic experiments using phages or host cells labeled with non-canonical amino acids [52] or stable isotopes [53] could be carried out to estimate the fate of microorganisms in complex environments. Moreover, prophages could be induced by stressing microbial communities with antibiotics, heat, acidic pH, or reactive oxygen species [54].

In summary, microbial communities in BGPS are affected by microbial interactions such as syntrophy, competition, and host-phage interactions. Further research is required to understand whether phage-induced cell lysis slows down the conversion of substrates to biogas or support growth of auxotrophic microbes by cycling of nutrients.

## Methods

All chemicals were at least of analysis grade. For nanoHPLC-MS/MS, MS grade solvents were used.

### Biogas plant sampling and reactor performance

Ten large-scale BGPs (BGP_02, BGP_03, BGP_04, BGP_05a, BGP_05b, BGP_07, BGP_09, BGP_10, BGP_X1, BGP_X2) and one laboratory-scale reactor BGP (BGP_X3) operating under stable process conditions were sampled twice about 1 month apart (T1, T2) (Table 1). Samples were stored at − 20 °C until further processing. BGP operators provided information about biogas production, feedstocks, fermenter content, process temperature, pH value, acid content, and TAN (Table 1).

### Metaproteomics workflow

Protein extraction was carried out in duplicates according to the protocol of Heyer et al. [55]. LC-MS/MS measurements were conducted according to Heyer et al. [29].

In brief, cell lysis and protein extraction were carried out simultaneously by phenol extraction in a ball mill. Extracted proteins were dissolved in a 2-mL aqueous solution containing 7 M urea, 2 M thiourea, and 0.01 g mL^−1^ 1,4-dithiothreitol. Amido black assay was used to quantify protein concentration [56, 57]. After acetone precipitation, proteins were separated by SDS-PAGE [58] using 500-μg protein extract. Subsequently, the SDS-PAGE lanes were sliced into ten fractions, proteins trapped in the gel were digested tryptically to peptides [59] and dried in a vacuum centrifuge (Digital Series SpeedVac SPD121P, Thermo Scientific, Waltham, USA). Before LC-MS/MS measurements, the samples were dissolved in 30-μL solvent A (98% LC-MS Water, 2% ACN, 0.05% TFA), centrifuged (30 min, 13.000×*g*, 4 °C), and transferred into a HPLC vial. Peptides were analyzed by LC-MS/MS using an UltiMate 3000 RSLCnano LC system, coupled online to an Orbitrap Elite™ Hybrid Ion Trap-Orbitrap MS (both from Thermo Fisher Scientific, Bremen, Germany). After injection, 8-μL peptides were loaded isocratically on a trap column (Dionex Acclaim, nano trap column, 100 μm i.d. × 2 cm, PepMap100 C18, 5 μm, 100 Å, nanoViper) with a flow rate of 7-μL min^−1^ chromatographic liquid phase A (98% LC-MS Water, 2% ACN, 0.05% TFA) for desalting and concentration.

Chromatographic separation was performed on a Dionex Acclaim PepMap C18 RSLC nano-reversed phase column (2-μm particle size, 100-Å pore size, 75-μm inner diameter, and 250-mm length) at 40 °C column temperature. A flow rate of 300 nL min^−1^ was applied using a binary A/B-solvent gradient (solvent A 98% LC-MS Water, 2% acetonitrile, 0.1% formic acid; solvent B 80% acetonitrile, 10% LC-MS water, 10% trifluorethanol, 0.1% formic acid) starting with 4% B for 4 min, continuing with a linear increase to 55% B for 120 min, followed by a column wash with 90% B for 5 min, and a re-equilibration with 4% B for 25 min. For MS acquisition, a data-dependent MS/MS method was chosen. MS was operated in positive ion mode, and precursor ions were acquired in the orbital trap of the hybrid MS at a resolution of 30,000 and a m/z range of 350–2000. Subsequently, the fragment ion scan was done in the linear ion trap of the hybrid MS with a mass range and a scan rate with “standard” parameter settings for the top 20 most intense precursors selected for collision-induced dissociation. “Active Exclusion” was adjusted to 5 s for two similar precursor ions.

### Data handling

We used the Proteome Discoverer Software (Thermo Fisher Scientific, Bremen, Germany, version 1.4.1.14) to convert raw mass spectral data into mascot generic files. Protein database searches were performed with OMSSA [60] and X!Tandem [61] using the MetaProteomeAnalyzer (version 1.3, www.mpa.ovgu.de) [31], requiring at least one identified peptide for a successful protein identification. Furthermore, protein database searches using Mascot [62] (Matrix Science, London, England, version 2.5.1) were carried out through the ProteinScape Software (Bruker Daltonics, Bremen, Germany, version 3.1.3461), and obtained results were imported into the MPA. Finally, OMSSA, X!Tandem, and Mascot results were merged. Search parameters for the protein database searches were trypsin, one missed cleavage, monoisotopic mass, carbamidomethylation (cysteine) as fixed modification, and oxidation (methionine) as variable modifications, ±10 ppm precursor and ± 0.5 Da MS/MS fragment tolerance, 1^13^C and + 2/+3 charged peptide ions. Results were controlled using a target-decoy strategy and a cutoff of 1% for the false discovery rate [63]. Validated single peptides were included in search results. The protein database contained sequences aggregated from UniProtKB/SwissProt (version 23.10.2014) [64] and seven metagenomes from BGP samples [20, 22, 23, 65]. The final FASTA database comprised 2.349.714 protein entries. All result files were submitted to PRIDE [66] with the accession number PXD009349. Unknown protein sequences from the metagenome were identified by BLAST (NCBI-Blast-version 2.2.31) [67] against UniProtKB/SwissProt requiring a maximum *e* value of 10^−4^. All BLAST hits with best *e* value were considered for further processing. Whenever possible, metaproteins were annotated with NCBI taxonomy [34], biological processes (UniProtKB keywords), UniRef [33], enzyme commission numbers (EC), and Kyoto Encyclopedia of Genes and Genomes (KEGG) Orthologies (KO) based on their UniProt entries [68]. Furthermore, redundant homologous proteins were grouped into metaproteins, based on UniRef50 [33]. Finally, metaprotein profiles were exported as comma-separated value files (csv). For visualization of taxonomic and functional results, chord diagrams [69] and krona plots [70] were created.

### Replicates and statistical analysis

Four replicates were measured for each biogas plant. Concerning the biological replicates, nearly no BGPs of more than 9000 BGPs in Germany are operated under completely identical process conditions. “Real” biological replicates are the samples BGP5a and BGP5b (two parallel fermenters of a single BGP, which were operated similarly) and the two identical lab scale fermenter. For the simulation of biological replicates for the other BGPs, we chose to sample fermenters operating at steady state (see Table 1 for chemical and technical parameters) at two time points 1 month apart. Each of the biological replicates was sampled twice to cover the variability of sampling and extraction. Overall the number of replicates was limited by available time for LC-MSMS measurement (more than 4 weeks) and for computational analysis (approx. 6 months).

For the comparison of the different metaproteins, microbial taxa and biological processes the associated spectral counts were normalized to the total spectral count of each measurement. In order to test the similarity between the samples and the reproducibility of our workflow we performed cluster analyses using Matlab (The MathWorks GmbH, Ismaningen, Germany, version 8.3.0.532 (R2014a)), the “cityblock” distance and an “average” linkage. During our data evaluation we focused on pathways, which were present in high abundance and only made statements about the presence or absence of different pathways. Comparisons of two groups of microbial communities/ biogas plants were validated by student's t-test and a p-value smaller than 0.05 was used as significance threshold.

## Additional files

Additional file 1: **Figure S1.** A–L. 12% SDS-PAGE of 11 BGPs loaded with 500 μg of total protein. For protein separation a 12% SDS-PAGE with 1.5 mm gel thickness was carried out and stained with colloidal coomassie dye solution. Proteins were extracted by combined phenol extraction in a ball mill. (STD) size standard. T1 and T2 refer to the first and second sampling time point. Ext1 and Ext2 represent two independent extractions. (PPTX 36926 kb)

Additional file 2: **Table S1.** List of identifications. Worksheet S1: List of all identified metaproteins. Worksheet S2: List of all identified microbial metaproteins (archaea and bacteria). Worksheet S3: List of all superkingdoms, Worksheet S4: List of all identified microbial families (archaea and bacteria). Worksheet S5: List of all identified biological processes. (XLSX 9006 kb)

Additional file 3: Note 1. Assignment of metaproteins mapped to biological processes involved in AD. (DOCX 361 kb)

Additional file 4: **Table S2.** Input files for chord diagram. (XLSX 101 kb)

Additional file 5: **Table S3.** Detailed assignment of microbial metaproteins and their role in biomass degradation focusing on A_Hydrolysis, B_Substrate_Uptake, C_Fermentation, D_AA_Metabolism, and E_Methanogenese. Metaproteins were grouped by EC or KO number, respectively, in the case of B_Substrate_Uptake. For the assignment of metaproteins to B_fermentation, archaea were excluded and for E_Methanogesis only archaea were considered. (XLSX 21299 kb)

Additional file 6: **Table S4.** Abundance of microbial key families, phages and, metaproteins related to microbial immune response. This excel sheet contains the detailed grouping of all metaproteins by their families as well as by their belonging to phages, and microbial immune response. It was the basis for Additional file 7: Table S5. (XLSX 18377 kb)

Additional file 7: **Table S5.** Abundance of main microbial families, host families of phages as well as the abundance of microbial immune response as represented by CRISPR proteins. Identified microbial metaproteins, phage metaprotein and CRISPR metaproteins were grouped by their (host) families and their spectral counts are shown as averages with the associated standard deviation. In contrast to the calculation of the phage abundance in Fig. 2, Additional file 12 this calculation considers also metaproteins that were assigned on root level, only. These metaprotein were assigned to phages based on their function. The abundance of the microbial families was normalized to the total number of identified microbial spectra. For the abundance of phages metaproteins and CRISPR metaproteins the spectral counts were normalized to the spectral counts of the corresponding microbial families. For a better overview the table was divided in A.) Bacterial families, B.) Archaeal families, C.) Others and D.) Overall. The detailed assignment can be found in Additional file 6: Table S4. F: taxonomic family; P: phage; C: CRISPR proteins. Differences between the abundances of phages assigned to archaea and to bacteria were validated by student’s t-test, showing with a p-value <0.00442 larger amounts of phages assigned to bacteria. For further details for the creation of this table please refer to Additional file 6: Table S4. (PDF 785 kb)

Additional file 8: **Table S6.** Overview about all antimicrobial peptides and proteins metaproteins. (XLSX 18 kb)

Additional file 9: **Figure S2.** Taxonomic profile of all identified viruses based on the number of identified viral spectra summed over all analyzed BGPs. (PNG 107 kb)

Additional file 10: **Figure S3.** Functional assignment of all identified phage spectra summed over all BGPs. (PNG 113 kb)

Additional file 11: Note 2. Estimation of the number of phage particles. (DOCX 21 kb)

Additional file 12: An interactive version of Fig. 2. (HTML 408 kb)

Additional file 13: An interactive version of Fig. 3. (ZIP 6150 kb)

## Abbreviations

BGP(s): Biogas plant(s)
CH_4_: Methane
CO_2_: Carbon dioxide
CSTR: Continuous stirred-tank reactor
EC: Enzyme commission number
H_2_: Hydrogen
HRT: Hydraulic retention time
KEGG: Kyoto Encyclopedia of Genes and Genomes
KO: KEGG Orthology
LC: Liquid chromatography
MS: Mass spectrometry/mass spectrometer
MS/MS: Tandem mass spectrometry/tandem mass spectrometer
OLR: Organic loading rate
TAN: Total ammonia nitrogen
TS: Total solids
TVFA/TA: Total volatile fatty acids to total alkalinity (non-dimensional)
VFA: Volatile fatty acids
VS: Volatile solids
UniRef: UniProt Reference Clusters
