# Supplementary material for: Metaproteome analysis reveals that syntrophy, competition, and phage-host interaction shape microbial communities in biogas plants
Source: Microbiome. 2019 Apr 27;7:69. doi: 10.1186/s40168-019-0673-y (PMC6486700; doi:10.1186/s40168-019-0673-y)
Supplement: Supplementary file 1 — Figure S1. A–L. 12% SDS-PAGE of 11 BGPs loaded with 500 μg of total protein. For protein separation a 12% SDS-PAGE with 1.5 mm gel thickness was carried out and stained with colloidal coomassie dye solution. Proteins were extracted by combined phenol extraction in a ball mill. (STD) size standard. T1 and T2 refer to the first and second sampling time point. Ext1 and Ext2 represent two independent extractions. (PPTX 36926 kb) [file 40168_2019_673_MOESM1_ESM.pptx]

## Slide 1
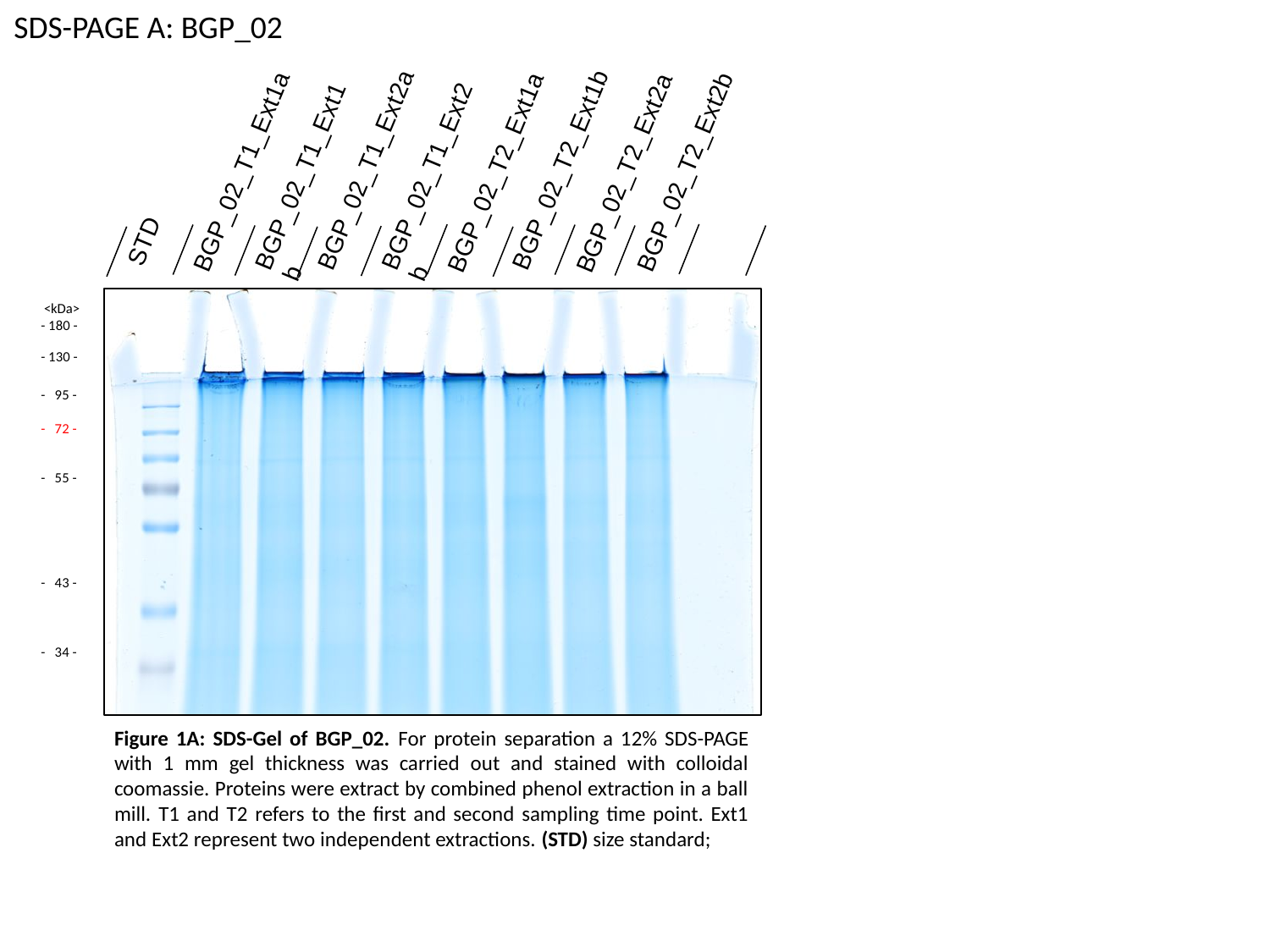

SDS-PAGE A: BGP_02
BGP_02_T1_Ext2a
BGP_02_T2_Ext1b
BGP_02_T2_Ext1a
BGP_02_T1_Ext1a
BGP_02_T2_Ext2a
BGP_02_T2_Ext2b
BGP_02_T1_Ext2b
BGP_02_T1_Ext1b
STD
 <kDa>
- 180 -
- 130 -
- 95 -
- 72 -
- 55 -
- 43 -
- 34 -
Figure 1A: SDS-Gel of BGP_02. For protein separation a 12% SDS-PAGE with 1 mm gel thickness was carried out and stained with colloidal coomassie. Proteins were extract by combined phenol extraction in a ball mill. T1 and T2 refers to the first and second sampling time point. Ext1 and Ext2 represent two independent extractions. (STD) size standard;

## Slide 2
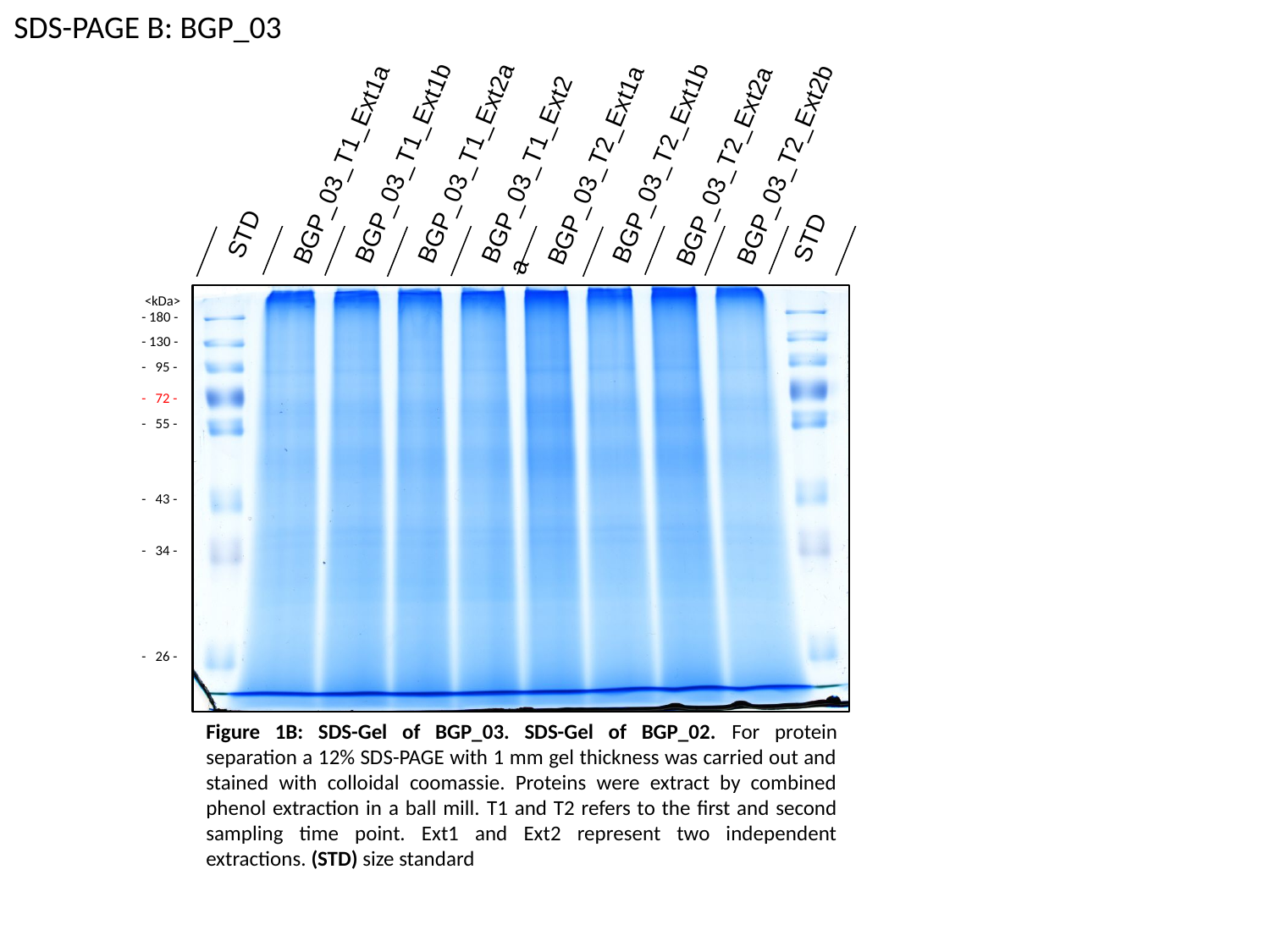

SDS-PAGE B: BGP_03
BGP_03_T1_Ext1b
BGP_03_T1_Ext2a
BGP_03_T2_Ext2b
BGP_03_T1_Ext1a
BGP_03_T2_Ext1b
BGP_03_T2_Ext1a
BGP_03_T2_Ext2a
BGP_03_T1_Ext2a
STD
STD
 <kDa>
- 180 -
- 130 -
- 95 -
- 72 -
- 55 -
- 43 -
- 34 -
- 26 -
Figure 1B: SDS-Gel of BGP_03. SDS-Gel of BGP_02. For protein separation a 12% SDS-PAGE with 1 mm gel thickness was carried out and stained with colloidal coomassie. Proteins were extract by combined phenol extraction in a ball mill. T1 and T2 refers to the first and second sampling time point. Ext1 and Ext2 represent two independent extractions. (STD) size standard

## Slide 3
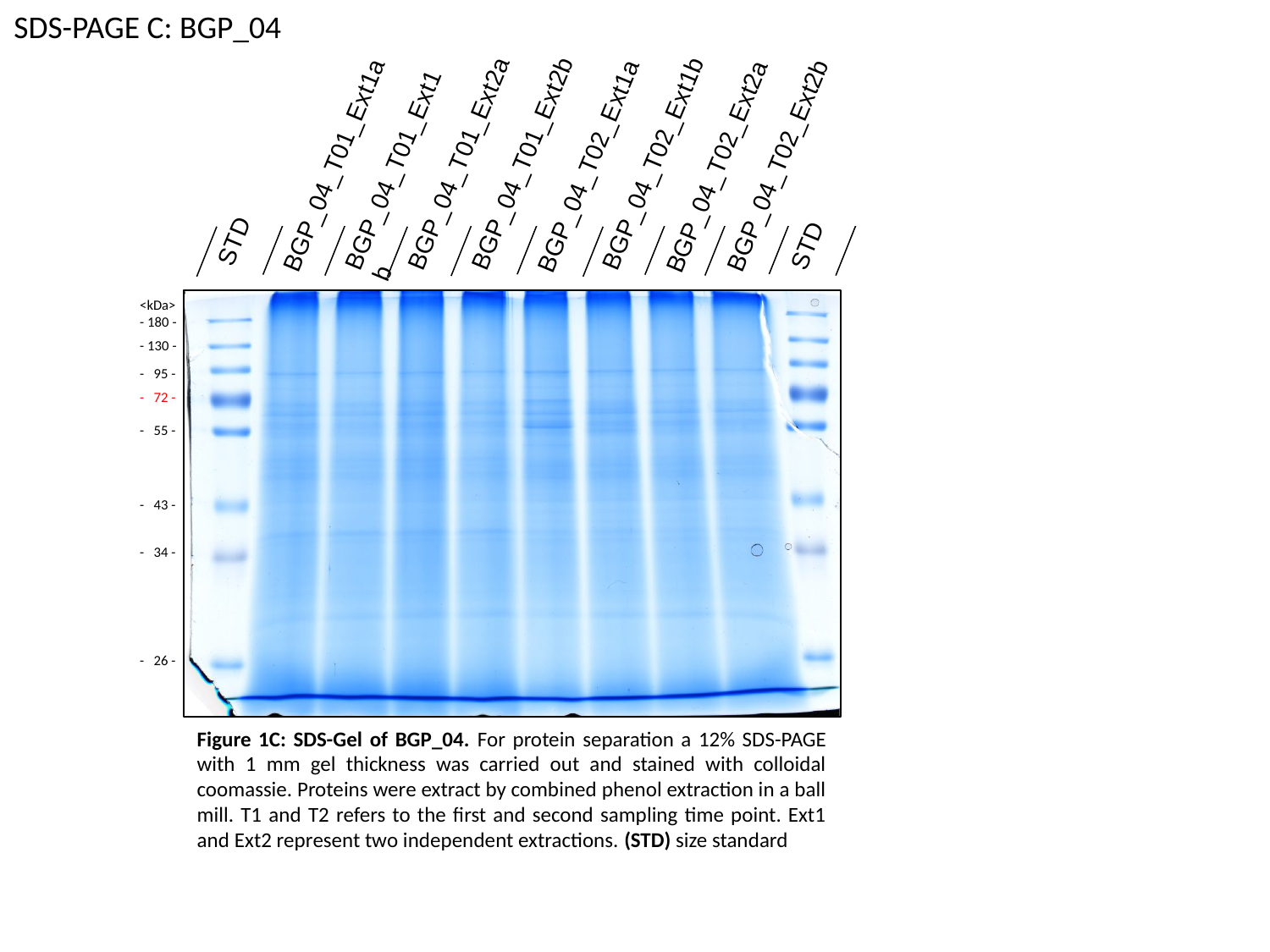

SDS-PAGE C: BGP_04
BGP_04_T02_Ext2a
BGP_04_T01_Ext2a
BGP_04_T01_Ext2b
BGP_04_T02_Ext1b
BGP_04_T02_Ext1a
BGP_04_T01_Ext1a
BGP_04_T02_Ext2b
BGP_04_T01_Ext1b
STD
STD
<kDa>
- 180 -
- 130 -
- 95 -
- 72 -
- 55 -
- 43 -
- 34 -
- 26 -
Figure 1C: SDS-Gel of BGP_04. For protein separation a 12% SDS-PAGE with 1 mm gel thickness was carried out and stained with colloidal coomassie. Proteins were extract by combined phenol extraction in a ball mill. T1 and T2 refers to the first and second sampling time point. Ext1 and Ext2 represent two independent extractions. (STD) size standard

## Slide 4
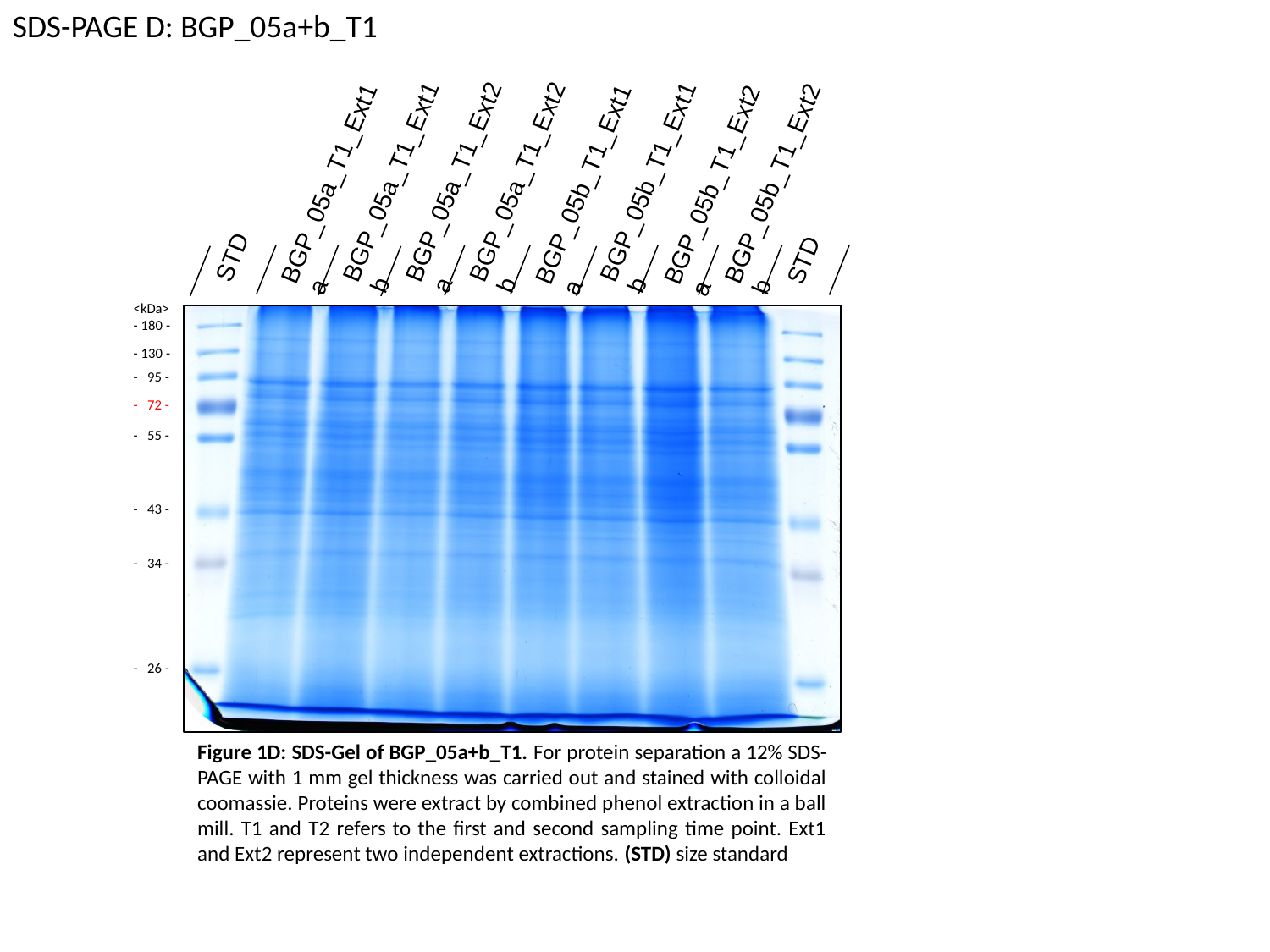

SDS-PAGE D: BGP_05a+b_T1
BGP_05b_T1_Ext1b
BGP_05a_T1_Ext2b
BGP_05a_T1_Ext2a
BGP_05b_T1_Ext1a
BGP_05a_T1_Ext1b
BGP_05b_T1_Ext2b
BGP_05b_T1_Ext2a
BGP_05a_T1_Ext1a
STD
STD
<kDa>
- 180 -
- 130 -
- 95 -
- 72 -
- 55 -
- 43 -
- 34 -
- 26 -
Figure 1D: SDS-Gel of BGP_05a+b_T1. For protein separation a 12% SDS-PAGE with 1 mm gel thickness was carried out and stained with colloidal coomassie. Proteins were extract by combined phenol extraction in a ball mill. T1 and T2 refers to the first and second sampling time point. Ext1 and Ext2 represent two independent extractions. (STD) size standard

## Slide 5
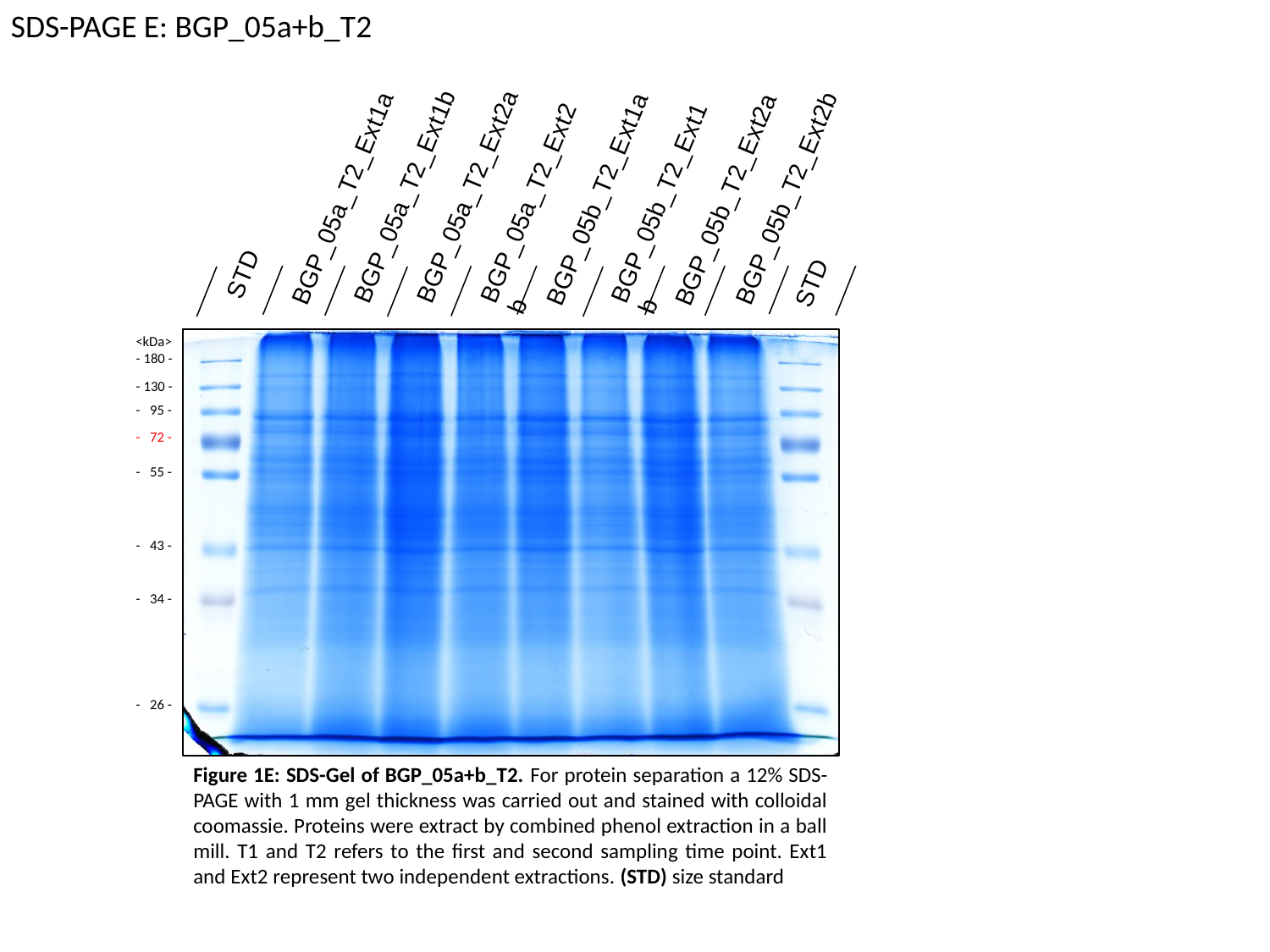

SDS-PAGE E: BGP_05a+b_T2
BGP_05a_T2_Ext1b
BGP_05a_T2_Ext1a
BGP_05b_T2_Ext1a
BGP_05b_T2_Ext2a
BGP_05a_T2_Ext2a
BGP_05b_T2_Ext2b
BGP_05a_T2_Ext2b
BGP_05b_T2_Ext1b
STD
STD
<kDa>
- 180 -
- 130 -
- 95 -
- 72 -
- 55 -
- 43 -
- 34 -
- 26 -
Figure 1E: SDS-Gel of BGP_05a+b_T2. For protein separation a 12% SDS-PAGE with 1 mm gel thickness was carried out and stained with colloidal coomassie. Proteins were extract by combined phenol extraction in a ball mill. T1 and T2 refers to the first and second sampling time point. Ext1 and Ext2 represent two independent extractions. (STD) size standard

## Slide 6
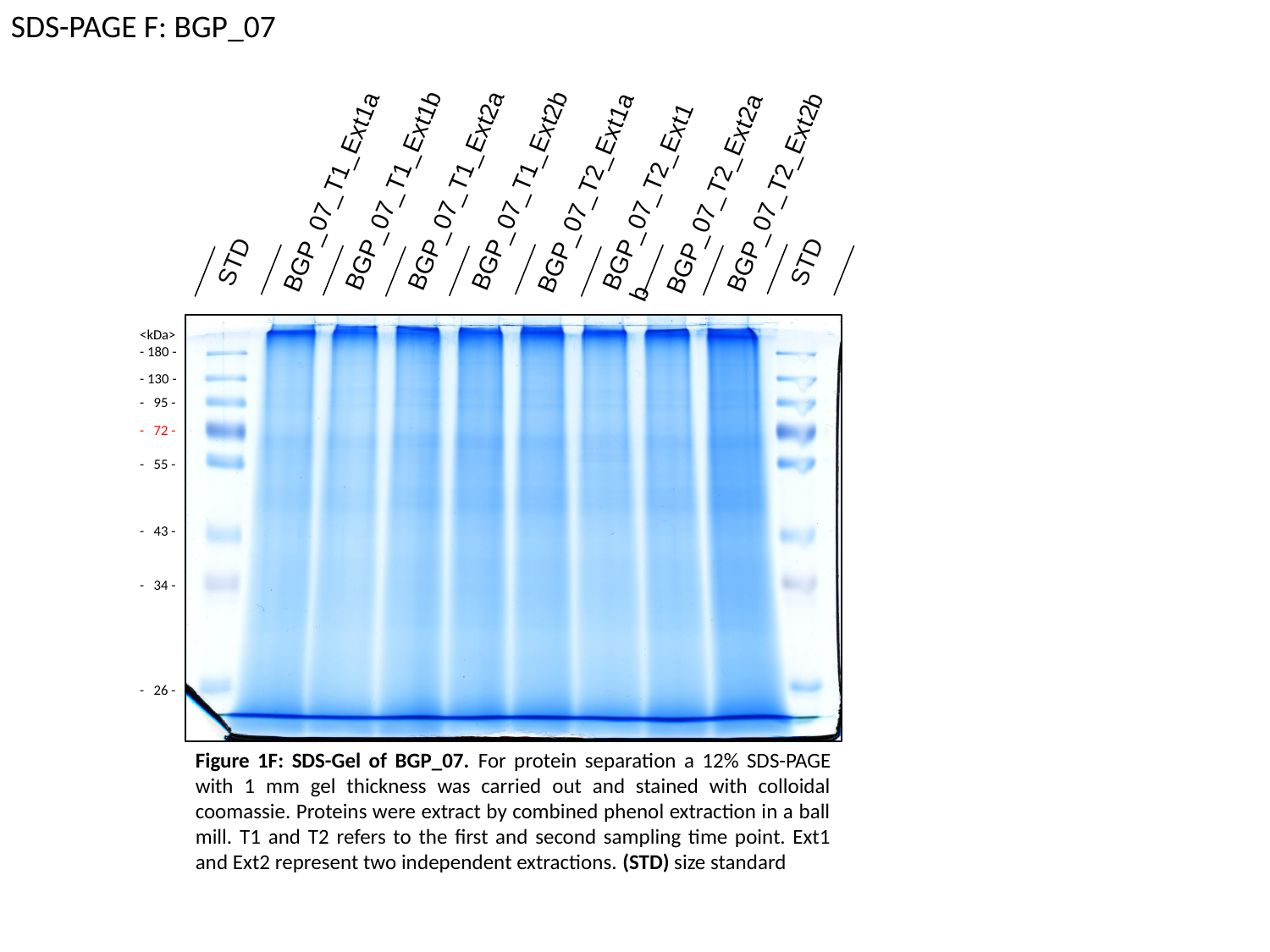

SDS-PAGE F: BGP_07
BGP_07_T2_Ext2b
BGP_07_T1_Ext1b
BGP_07_T1_Ext1a
BGP_07_T2_Ext2a
BGP_07_T1_Ext2b
BGP_07_T2_Ext1a
BGP_07_T1_Ext2a
BGP_07_T2_Ext1b
STD
STD
<kDa>
- 180 -
- 130 -
- 95 -
- 72 -
- 55 -
- 43 -
- 34 -
- 26 -
Figure 1F: SDS-Gel of BGP_07. For protein separation a 12% SDS-PAGE with 1 mm gel thickness was carried out and stained with colloidal coomassie. Proteins were extract by combined phenol extraction in a ball mill. T1 and T2 refers to the first and second sampling time point. Ext1 and Ext2 represent two independent extractions. (STD) size standard

## Slide 7
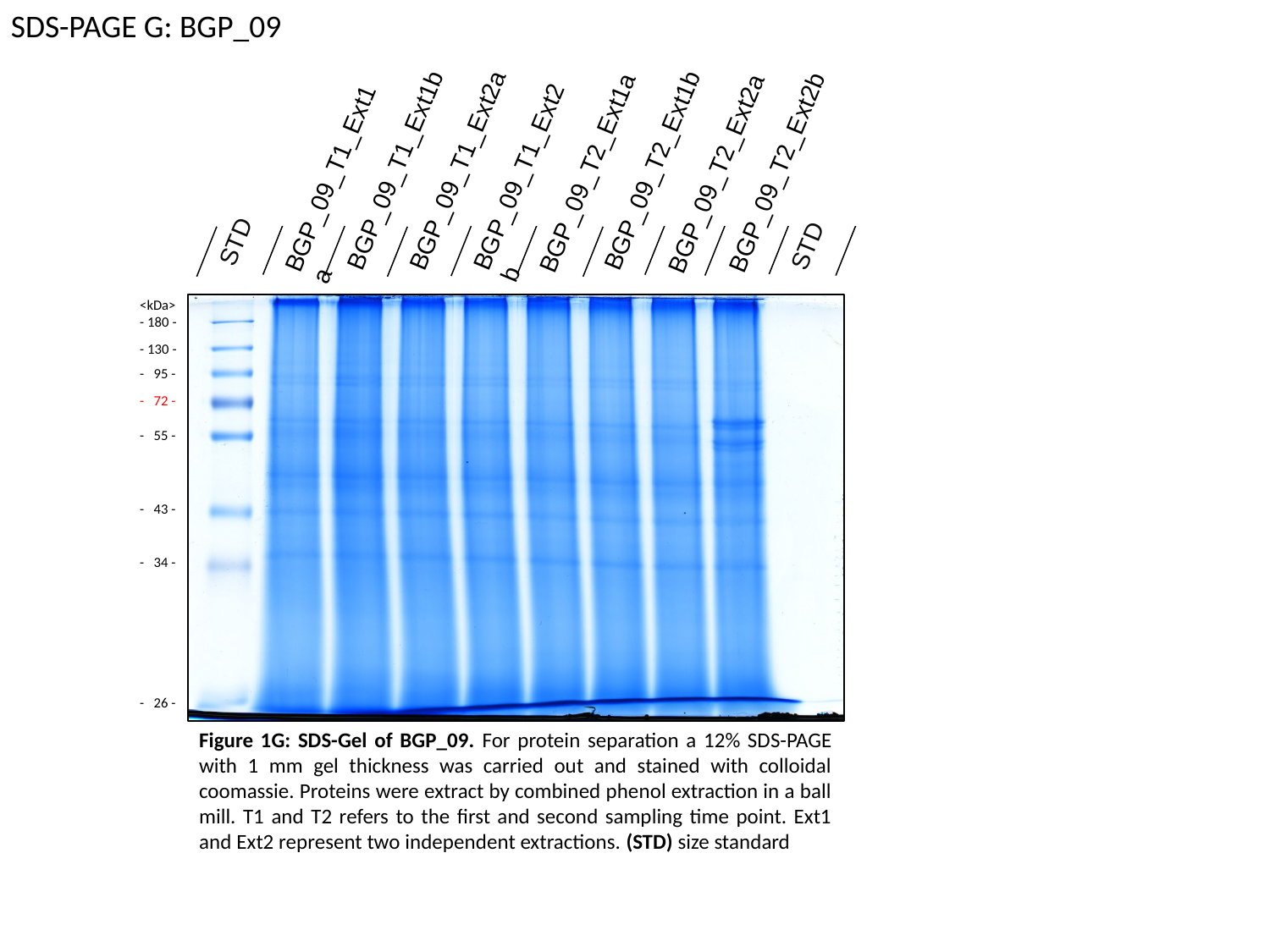

SDS-PAGE G: BGP_09
BGP_09_T2_Ext1b
BGP_09_T1_Ext2a
BGP_09_T2_Ext2b
BGP_09_T2_Ext2a
BGP_09_T1_Ext1b
BGP_09_T2_Ext1a
BGP_09_T1_Ext1a
BGP_09_T1_Ext2b
STD
STD
<kDa>
- 180 -
- 130 -
- 95 -
- 72 -
- 55 -
- 43 -
- 34 -
- 26 -
Figure 1G: SDS-Gel of BGP_09. For protein separation a 12% SDS-PAGE with 1 mm gel thickness was carried out and stained with colloidal coomassie. Proteins were extract by combined phenol extraction in a ball mill. T1 and T2 refers to the first and second sampling time point. Ext1 and Ext2 represent two independent extractions. (STD) size standard

## Slide 8
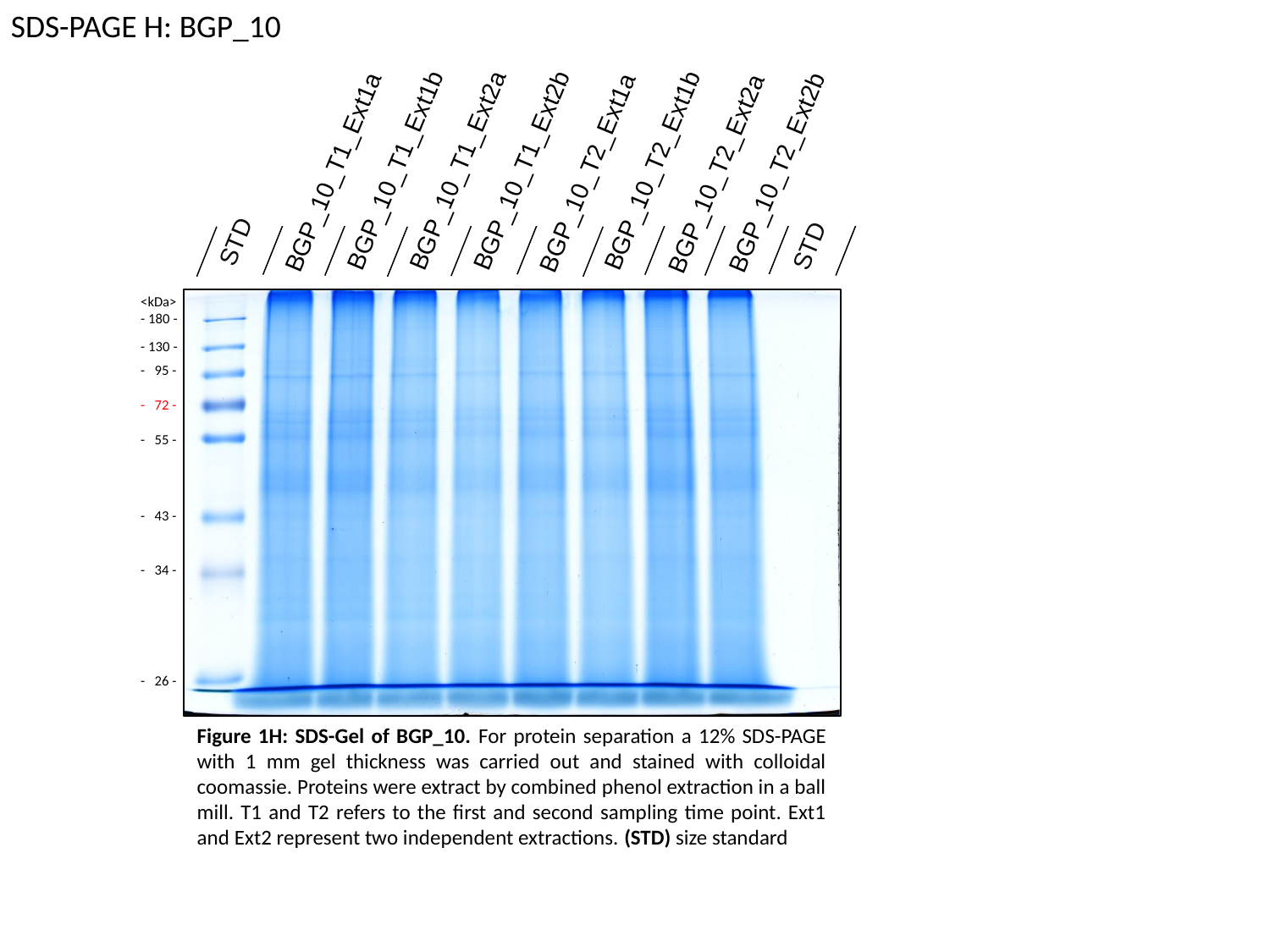

SDS-PAGE H: BGP_10
BGP_10_T1_Ext1b
BGP_10_T2_Ext2a
BGP_10_T1_Ext2a
BGP_10_T2_Ext2b
BGP_10_T2_Ext1b
BGP_10_T2_Ext1a
BGP_10_T1_Ext1a
BGP_10_T1_Ext2b
STD
STD
<kDa>
- 180 -
- 130 -
- 95 -
- 72 -
- 55 -
- 43 -
- 34 -
- 26 -
Figure 1H: SDS-Gel of BGP_10. For protein separation a 12% SDS-PAGE with 1 mm gel thickness was carried out and stained with colloidal coomassie. Proteins were extract by combined phenol extraction in a ball mill. T1 and T2 refers to the first and second sampling time point. Ext1 and Ext2 represent two independent extractions. (STD) size standard

## Slide 9
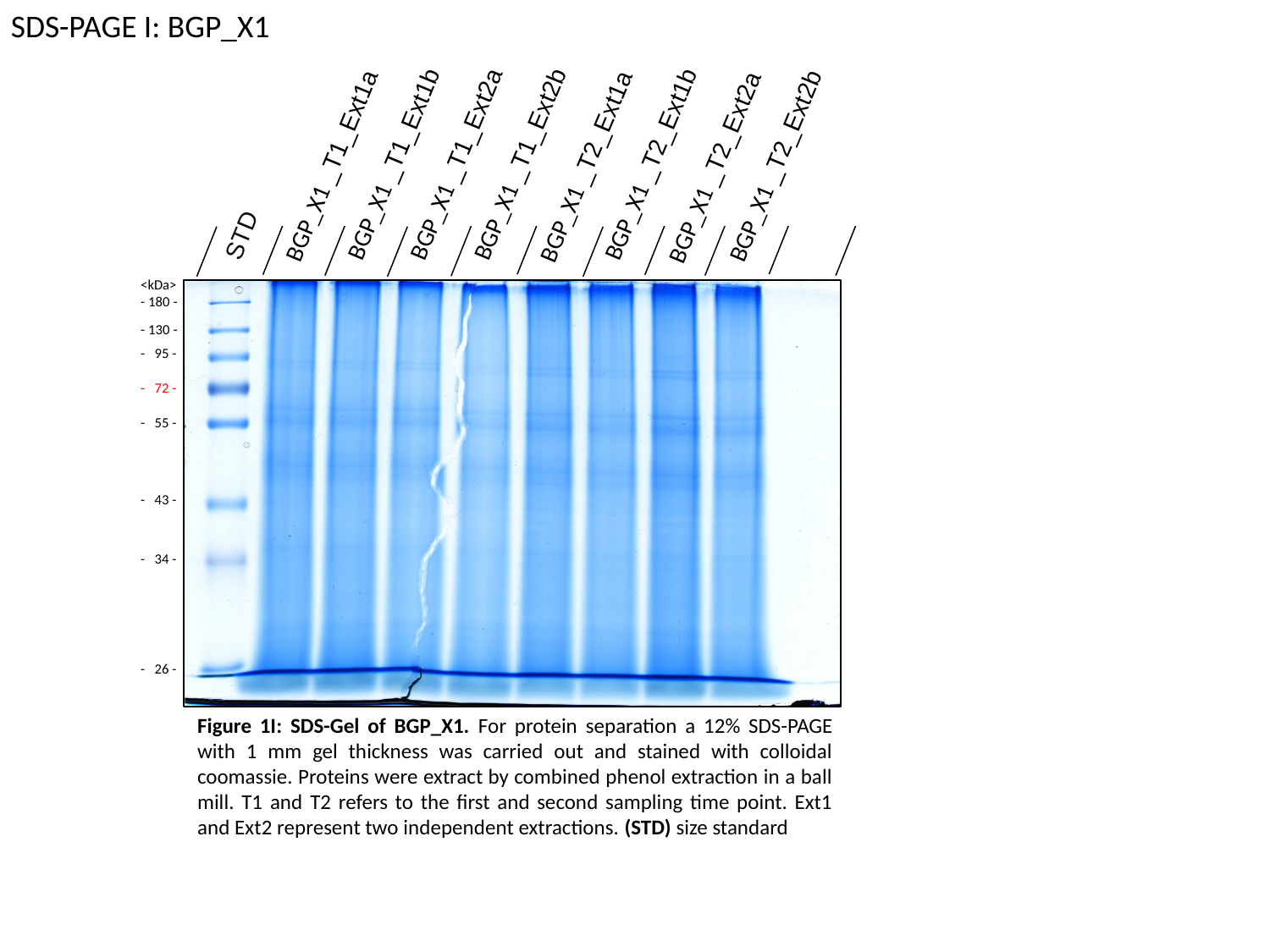

SDS-PAGE I: BGP_X1
BGP_X1 _T2_Ext1b
BGP_X1 _T1_Ext1b
BGP_X1 _T2_Ext2a
BGP_X1 _T2_Ext1a
BGP_X1 _T2_Ext2b
BGP_X1 _T1_Ext2a
BGP_X1 _T1_Ext2b
BGP_X1 _T1_Ext1a
STD
<kDa>
- 180 -
- 130 -
- 95 -
- 72 -
- 55 -
- 43 -
- 34 -
- 26 -
Figure 1I: SDS-Gel of BGP_X1. For protein separation a 12% SDS-PAGE with 1 mm gel thickness was carried out and stained with colloidal coomassie. Proteins were extract by combined phenol extraction in a ball mill. T1 and T2 refers to the first and second sampling time point. Ext1 and Ext2 represent two independent extractions. (STD) size standard

## Slide 10
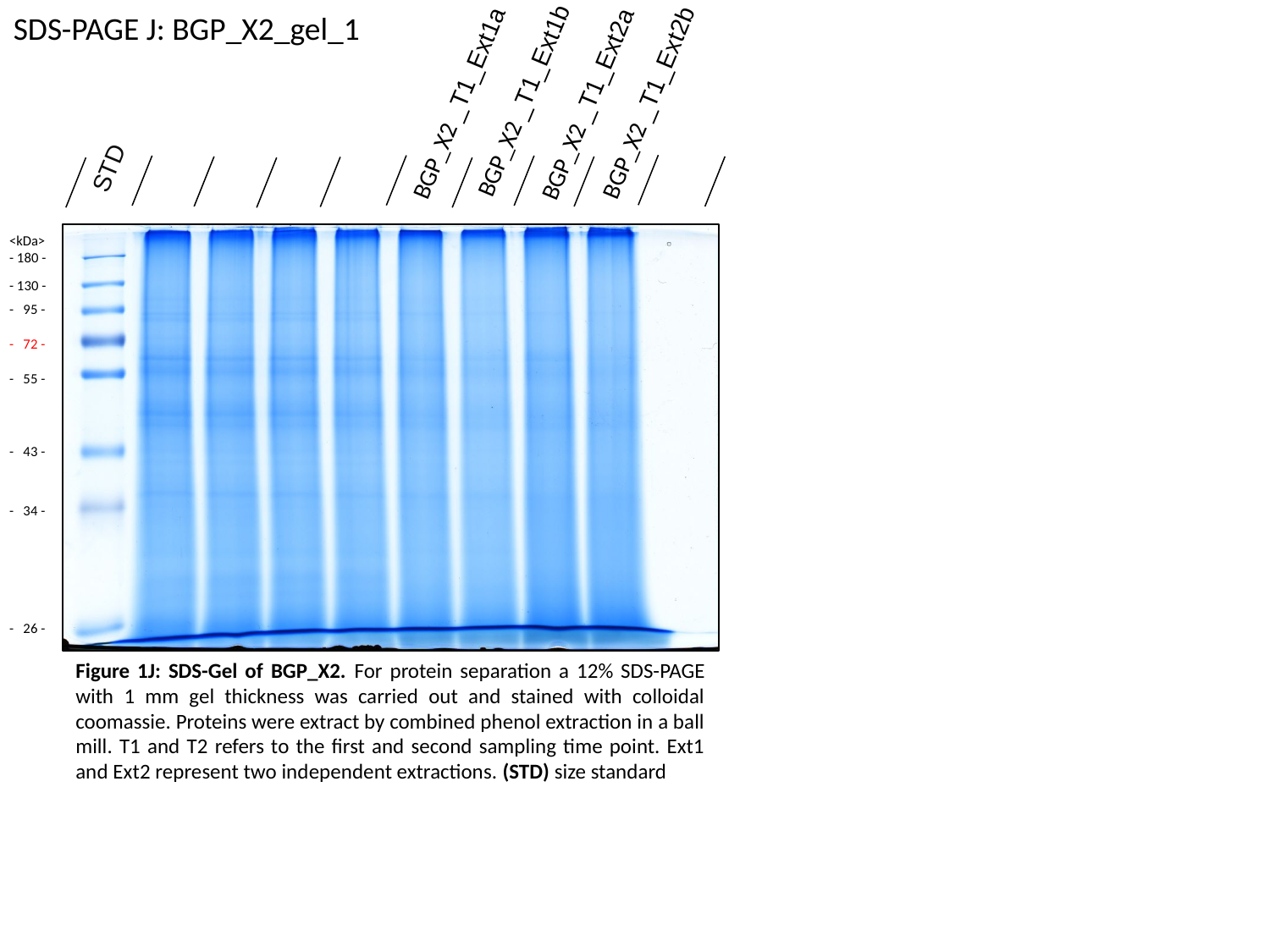

BGP_X2 _T1_Ext1b
BGP_X2 _T1_Ext2a
BGP_X2 _T1_Ext1a
BGP_X2 _T1_Ext2b
STD
SDS-PAGE J: BGP_X2_gel_1
<kDa>
- 180 -
- 130 -
- 95 -
- 72 -
- 55 -
- 43 -
- 34 -
- 26 -
Figure 1J: SDS-Gel of BGP_X2. For protein separation a 12% SDS-PAGE with 1 mm gel thickness was carried out and stained with colloidal coomassie. Proteins were extract by combined phenol extraction in a ball mill. T1 and T2 refers to the first and second sampling time point. Ext1 and Ext2 represent two independent extractions. (STD) size standard

## Slide 11
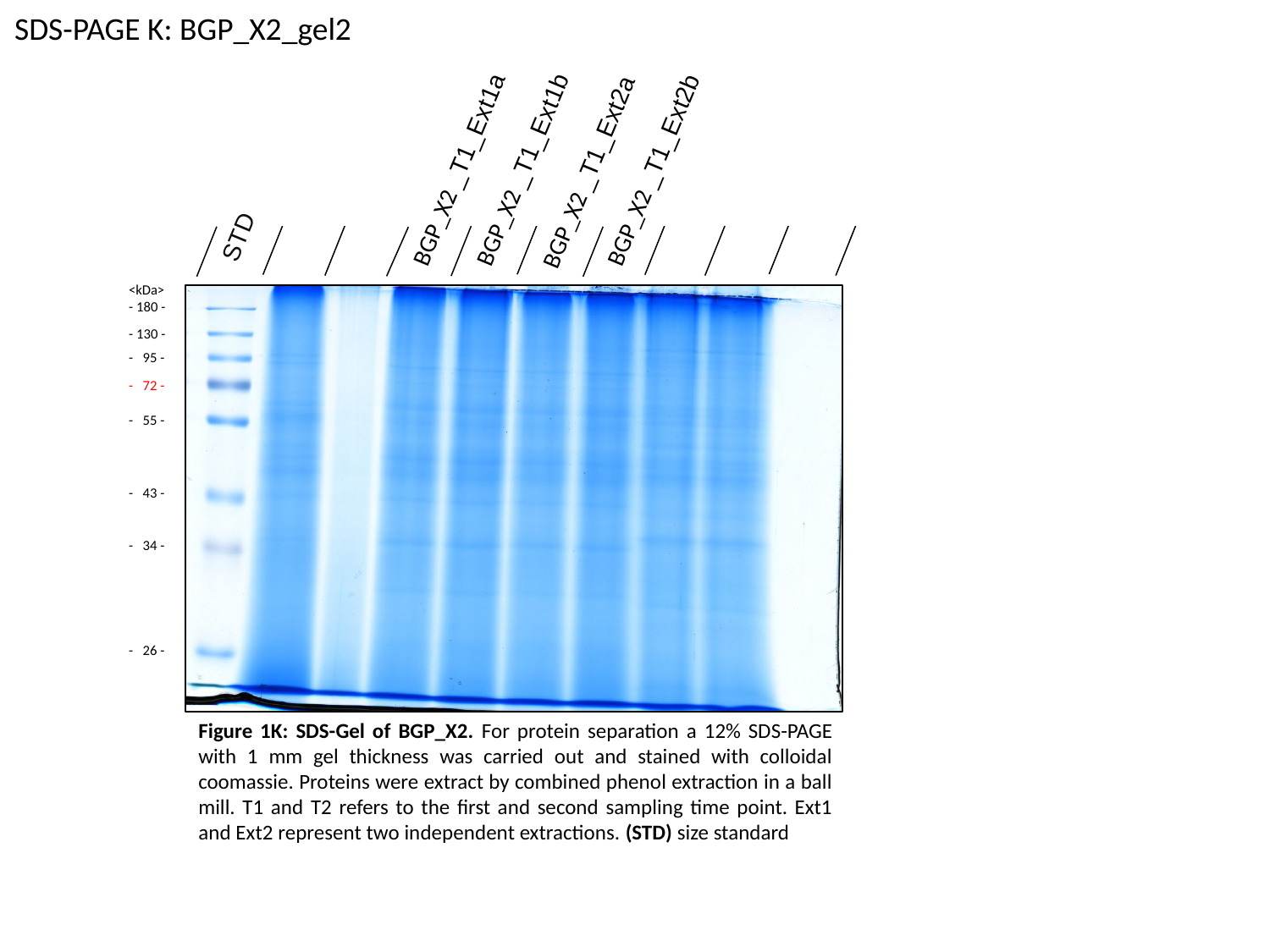

BGP_X2 _T1_Ext2b
BGP_X2 _T1_Ext2a
BGP_X2 _T1_Ext1a
BGP_X2 _T1_Ext1b
STD
SDS-PAGE K: BGP_X2_gel2
<kDa>
- 180 -
- 130 -
- 95 -
- 72 -
- 55 -
- 43 -
- 34 -
- 26 -
Figure 1K: SDS-Gel of BGP_X2. For protein separation a 12% SDS-PAGE with 1 mm gel thickness was carried out and stained with colloidal coomassie. Proteins were extract by combined phenol extraction in a ball mill. T1 and T2 refers to the first and second sampling time point. Ext1 and Ext2 represent two independent extractions. (STD) size standard

## Slide 12
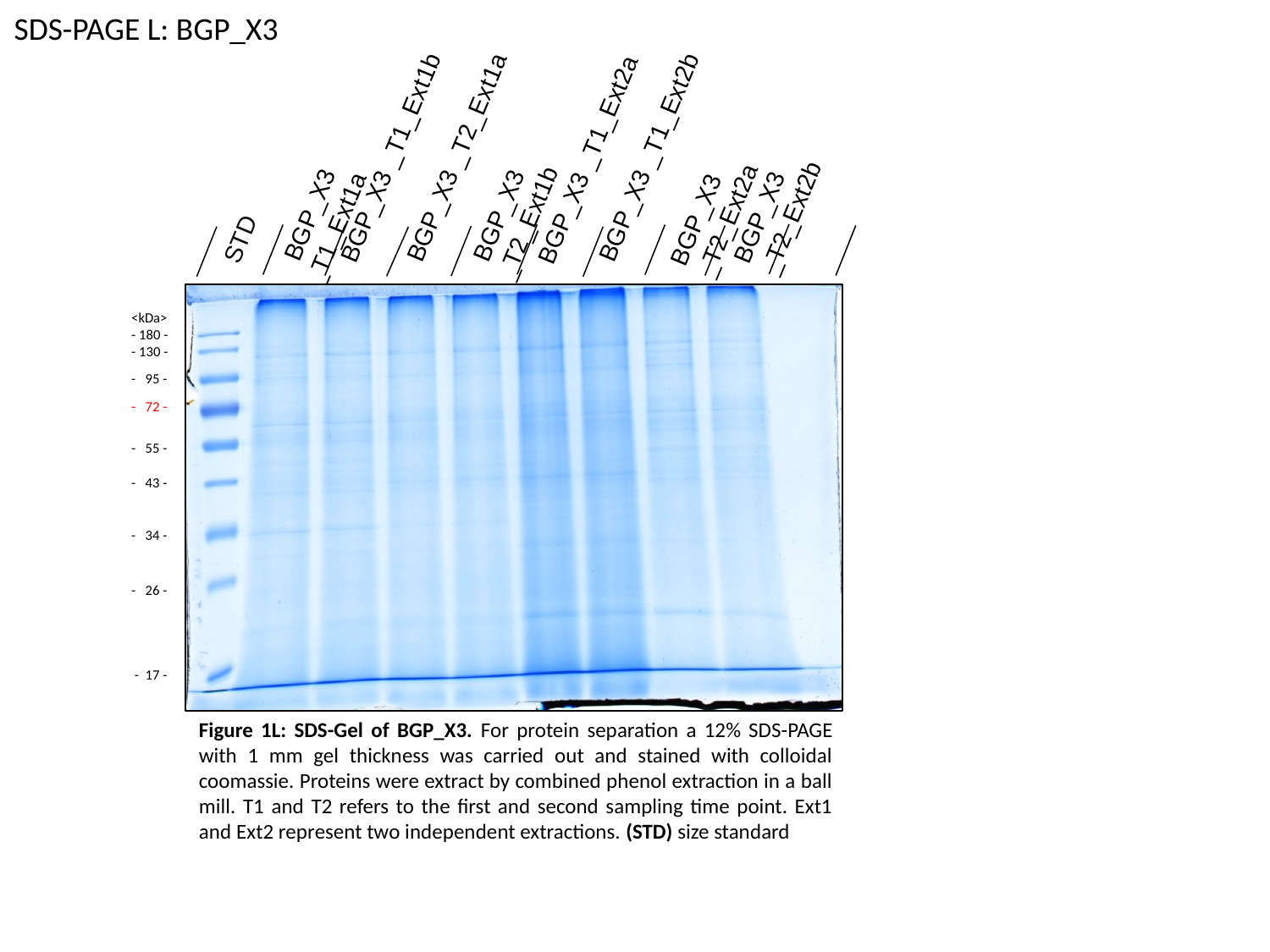

BGP_X3 _T1_Ext2b
 BGP_X3 _T1_Ext2a
 BGP_X3 _T2_Ext1a
 BGP_X3 _T1_Ext1b
 BGP_X3 _T1_Ext1a
BGP_X3 _T2_Ext2b
BGP_X3 _T2_Ext2a
 BGP_X3 _T2_Ext1b
SDS-PAGE L: BGP_X3
STD
<kDa>
- 180 -
- 130 -
- 95 -
- 72 -
- 55 -
- 43 -
- 34 -
- 26 -
 - 17 -
Figure 1L: SDS-Gel of BGP_X3. For protein separation a 12% SDS-PAGE with 1 mm gel thickness was carried out and stained with colloidal coomassie. Proteins were extract by combined phenol extraction in a ball mill. T1 and T2 refers to the first and second sampling time point. Ext1 and Ext2 represent two independent extractions. (STD) size standard
