# Supplementary material for: Metaproteome analysis reveals that syntrophy, competition, and phage-host interaction shape microbial communities in biogas plants
Source: Microbiome. 2019 Apr 27;7:69. doi: 10.1186/s40168-019-0673-y (PMC6486700; doi:10.1186/s40168-019-0673-y)
Supplement: Supplementary file 3 — Note 1. Assignment of metaproteins mapped to biological processes involved in AD. (DOCX 361 kb) [file 40168_2019_673_MOESM3_ESM.docx]

Additional Note1:

Assignment of metaproteins mapped to biological processes involved in AD

The strength of metaproteomics is that individual metaproteins can be quantified and mapped to actually occurring biological processes in AD. Detailed assignments of metaproteins to hydrolysis (Additional Table 3 A_Hydrolysis) and substrate uptake (Additional Table 3 Table B_Substrate_Uptake), fermentation pathways (Additional Table 3 C_Fermentation), amino acid metabolism (Additional Table 3 D_AA Metabolism) and CH_4_ production through methanogenesis (Additional Table 3 E_Methanogenese) were prepared.

### Hydrolysis

Polysaccharides constitute the main component of substrates, thus most hydrolytic enzymes could be assigned to polysaccharide degradation (Additional Table 3 A_Hydrolysis). For example, pullanases (EC 3.2.1.41) and glucanases (EC 3.2.1.4 and EC 3.2.1.91) as well as beta-xylanases (EC 3.2.1.8) were involved in degradation of glucans and hemicellulose. No significant amounts of specific lignin degrading enzymes such as laccase (EC 1.11.1.19) and peroxidases (EC 1.11.1.19) were found^1^. The presence of the enzyme 6-oxocyclohex-1-ene-1-carbonyl-CoA hydrolase (EC 3.7.1.21) proofs the anaerobic metabolisms of aromatic compounds^2^, but does not specify if lignin was significantly degraded.

The abundance of proteinases (EC 3.4.21-25.X), aminopeptidases (EC 3.4.11.-), the enzymes glycerophosphoryl diester phosphodiesterase (EC 3.1.4.46) and long-chain-fatty-acid--CoA ligase (EC 6.2.1.3) showed that protein degradation plays a more important role than lipid metabolism.

Although most hydrolases were expressed in all BGPs, several differences were observed. Degradation of cellulose (enzymes e.g. endoglucanase, EC 3.2.1.4, exoglucanase, EC 3.2.1.91), fatty acids (long-chain-fatty-acid--CoA ligase, EC: 6.2.1.3) and aromatic compounds (6-oxocyclohex-1-ene-1-carbonyl-CoA hydrolase, EC 3.7.1.21) was more abundant in mesophilic BGPs, while thermophilic BGPs possessed larger amounts of enzymes involved in degradation of hemicellulose (alpha-L-arabinofuranosidase, EC 3.2.1.55), starch (glucosidase, EC 3.2.1.20) and pullan (pullulanase, EC 3.2.1.41).

### Uptake of substrate

Transport proteins where categorized according to their ligands as far as this information was available uptake (Additional Table 3 Table B_Substrate_Uptake, Table A1).

Transport of sugars (65.8%) constitutes the largest group of transporters. A high amount of peptide transporters (26.4%) suggesting an important role in the microbial communities was detected, too. Almost no transporters for fermentation products such as short chain fatty acids and alcohols were found. Minor amounts of a formate transport (1.2%) were detected only. The formate transporter indicated for syntrophic formate exchange. Finally, a several transporters for co-factors were found: vitamin B12 (0.2%), folate (0.4%) and thiamine (0.4%).

Table A1: Abundance of transport metaproteins based on the number of identified spectra and grouped by the transported components.

### Analysis of fermentation pathways

Microorganisms in BGPs utilize sugars through fermentation processes (Additional Table 3 C_Fermentation). First fermentation pathways are glycolysis and the pentose phosphate pathway producing the central metabolite pyruvate and acetyl-CoA. For recycling NAD^+^ as electron acceptor NADH produced during sugar degradation is consumed by fermentation of pyruvate and acetyl-CoA to acetate, formate, ethanol, lactate, propionate and butyrate (Fig. A1). The corresponding metaproteins for these pathways were found at different levels in the eleven BGPs studied (Additional

Table 3 C_Fermentation). Furthermore, metaproteins suggesting the formation of acetoin and succinate were identified in smaller quantities. Contrary, no enzymes involved in the formation of valeriate were detected.

Subsequent to primary fermentation, alcohols and short chain fatty acids were further degraded to acetate, CO_2_ and H_2_coupled to hydrogenases. However, differentiation between primary and secondary fermentation is difficult due to unspecific taxonomic classification of many metaproteins and the fact that they catalyze these fermentation reactions in both directions (e.g. lactate dehydrogenase). More specific assignment of these metaproteins to taxonomies would enable a better differentiation between primary and secondary fermentation. The correlation of metaproteins to process parameters (Additional Table 3 C_Fermentation) revealed a negative correlation between propionate fermentation and TAN and a positive correlation between temperature and the glycolysis or pentose phosphate pathway.

**
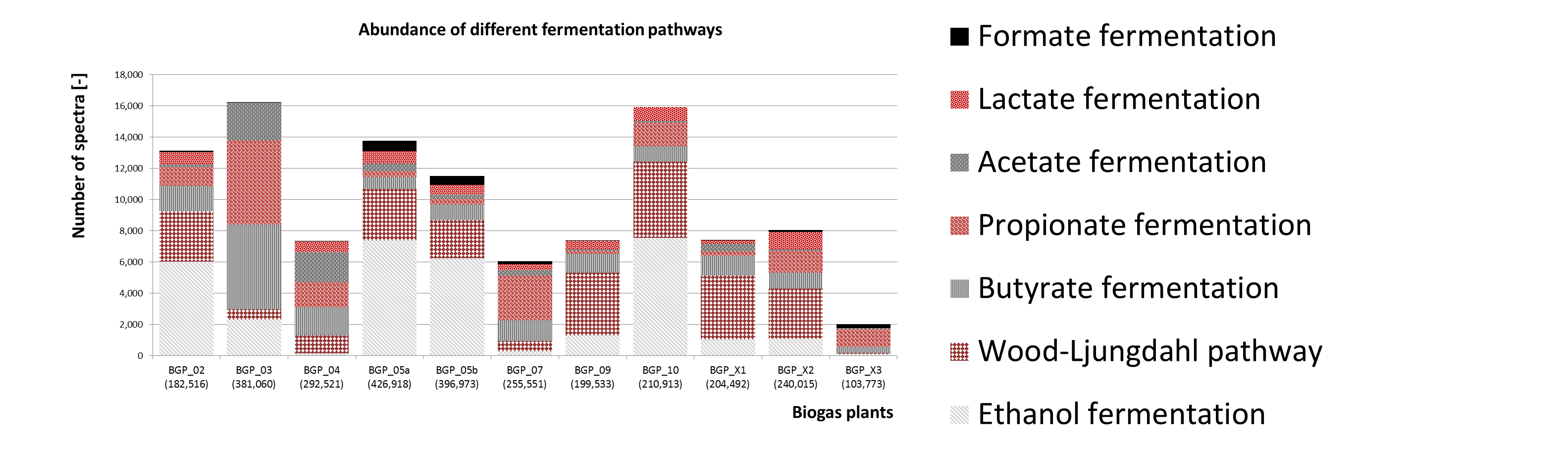
**

Figure A1: Abundance of the different fermentation pathways.

Spectral counts of representative metaproteins for each fermentation pathway (Additional Table 3 C_Fermentation) were summed and normalized to the total spectral count of the considered fermentation pathways for each BGP.

**Amino acid metabolism**

BGPs are fed with significant amounts of protein rich substrate. Metaproteins for the degradation of all 20 amino acids and ornithine as well as large amounts of protein and peptide transporters were detected (Additional Table 3 D_AA Metabolism). While the main sources of amino acids are proteins, glycine is also released by the degradation of nucleotides (e.g. Phosphoribosylamine--glycine ligase, EC 6.3.4.13). Additionally, proteins involved in the biosynthesis of amino acids were also identified. For example, the enzymes 4-hydroxy-tetrahydrodipicolinate reductase (EC 1.17.1.8) *and 4-hydroxy-tetrahydrodipicolinate synthase (EC 4.3.3.7)* were involved in the biosynthesis of lysine.

Abundance of metaproteins for the metabolism of different amino acid varied significantly (Additional Table 3 D_AA Metabolism). High amounts of metaproteins for the metabolism of the acidic amino acid glutamate as well as the polar and neutral amino acids glycine, serine and cysteine could be detected. Furthermore, the enzymes for the metabolism of unpolar and hydrophobic amino acids isoleucine, leucine and valine were identified in large amounts, too.

The most abundant metaproteins associated to the amino acid metabolism were glutamate dehydrogenase (EC 1.4.1.4) and glycine reductase (EC 1.21.4.2). In the case of amino acid degradation, glutamate dehydrogenase deaminates glutamate to 2-oxoglutarate and releases ammonia. Glutamate is produced from 2-oxoglutarate during transaminase reactions (such as the branched-chain amino acid aminotransferase (EC 2.6.1.42) and deaminated by glutamate dehydrogenase to release inorganic nitrogen as ammonia from biomass.

Glycine reductase deaminates glycine to acetyl-phosphate. This reaction was found to be coupled to the degradation of glycine by the glycine cleavage system in order to balance the redox potential^3^. Whereas glycine reductase consumes two electrons, the glycine cleavage system and subsequent degradation to CO_2_ produces six electrons (EC 1.4.4.2 glycine dehydrogenase (decarboxylation), EC 2.1.2.10 aminomethyltransferase, EC 1.8.1.4 dihydrolipoamide dehydrogenase, K02437 glycine cleavage system H protein). The correlation between glycine reductase and the glycine cleavage system in BGPs confirmed these previous results.

**Homoacetogenesis or syntrophic acetate oxidation**

The Wood-Ljungdahl pathway allows the conversion of acetate to CO_2_ and H_2_ (syntrophic acetate oxidation) or *vice versa* (homacetogenesis). Expression of metaproteins associated to these pathways was detected and will be discussed in the chapter methanogenesis, as these pathways are closely related to the utilization of H_2_.

**Methanogenesis**

Hydrogenotrophic and acetoclastic methanogenesis were confirmed as the dominant pathways to produce CH_4_ by the number of metaproteins detected (Fig. A2)^4^. Additionally, enzymes for the methylotrophic production of CH_4_ from methylamines and methanol were identified (Additional Table 3 E_Methanogenese). A clear distinction emerged between strictly hydrogenotrophic BGPs and BGPs producing CH_4_ through both pathways: acetoclastic and hydrogenotrophic methanogenesis. In congruence with previous results no acetoclastic methanogenesis was found in thermophilic BGPs 05_1 and 05_2^5^. The mesophilic BGP 04, showed the largest abundance of acetoclastic and methylotrophic methanogenesis among all samples. The different methanogenesis pathways converge in the production of 5-methyltetrahydromethanopterin, which is converted into CH_4_ by the enzymes tetrahydromethanopterin S-methyltransferase (EC 2.1.1.86) and methyl-CoM reductase (EC 2.8.4.1). The abundance of both metaproteins indicated the amount of methanogenic archaea present in the BGPs^6, 7^. Whereas differences in the amounts of both enzymes were observed (Fig. A2), the ratio between both proteins remained nearly constant (data not shown). The lowest abundance of both enzymes was found in the in thermophilic BGPs 05_1 and 05_2.


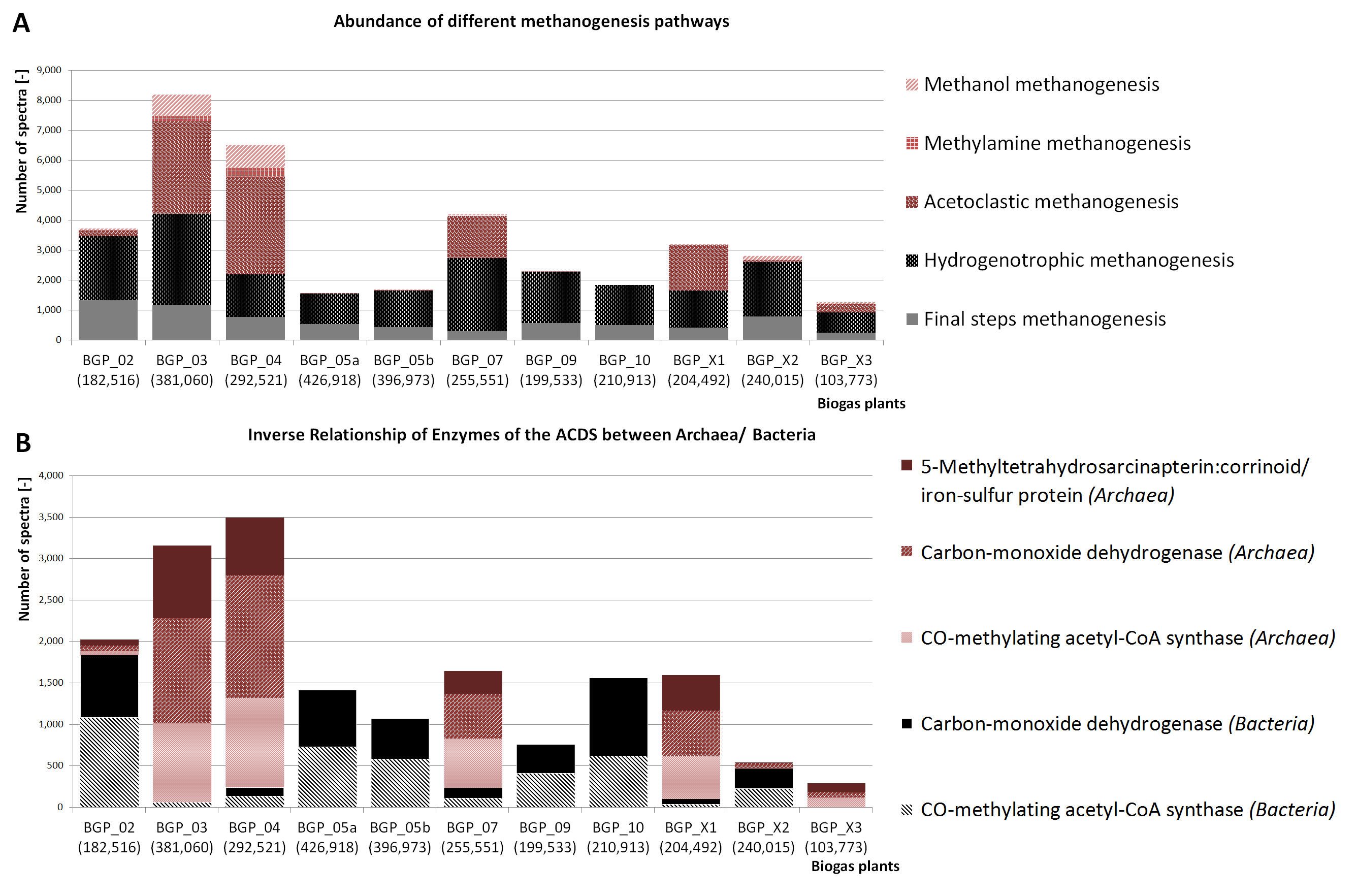


Figure A2: Abundance of archaeal and bacterial acetyl-CoA decarbonylase/synthase (ACDS) as well as of methanogenesis pathways. Spectral counts of representative metaproteins for A. methanogenesis pathway and B. each ACDS metaprotein (Additional Table 3 E_Methanogenese) sorted by archaeal and non-archaeal and summed. The back bars indicate bacterial one carbon metabolism and hydrogenotrophic methanogenesis. The red bars are associated with either acetoclastic methanogenesis or acetoclastic methanogenesis as well as the methanol and methylamine pathways. Equals Fig. 4 in the main manuscript.

Since acetate is a ubiquitous product of bacterial fermentation, it has to be converted into CH_4_ even in strictly hydrogenotrophic BGPs. As an alternative to archaeal acetyl-CoA decarboxylase/synthase complexes, certain bacteria possess also an acetyl-CoA decarboxylase/synthase complex (ACDS) but lacking the gamma-delta components. Bacteria use this complex for either homoacetogenesis or syntrophic acetate oxidation. A negative correlation between archaeal ACDS*^8^* and bacterial ACDS was observed (Fig. A2): high amounts of ACDS in archaea correlates with low amounts of ACDS in bacteria and *vice versa*.

BGP_02, 05_1, BGP_05_2, BGP_09, BGP_10, BGP_X2 are dominated by bacterial ACDS, indicating either homacetogenesis or syntrophic acetate oxidation but no acetoclastic methanogenesis. Furthermore many dehydrogenases and other proteins related to the Wood-Ljungdahl pathway were specific for this group of BGPs. The high abundance of bacterial ACDS did not significantly correlate with process parameters. A small tendency towards BGPs with higher total acid amount and higher temperature can be observed (Additional Table 3 E_Methanogenese). In contrast the second group of BGPs possessed larger amounts of archaeal ACDS but no bacterial ACDS. Further proteins e.g. acetate kinase (EC 2.7.2.1) and V-type ATP synthases (EC 3.6.3.14) co-occurring with archaeal ACDS complex components provide further evidence for acetoclastic methanogenesis.

**Discussion**

The results of this investigation confirmed previous studies^5, 9, 10, 11, 12, 13, 14^.

### Hydrolysis & fermentation

The identified hydrolytic enzymes proof the degradation of glucans, hemicelluloses, proteins and, to a limited extent, aromatic compounds and lipids. In contrast, no specific enzymes for the degradation of lignin^1^ were observed, matching the insufficient lignin degradation under anaerobic conditions. Transporters for the expected hydrolysis products, peptides and sugars, were found abundantly. Fermentation of substrates started with either glycolysis, pentose phosphate pathway or individual degradation pathways of amino acids. Finally, metaproteins for the following fermentation pathways producing H_2_, CO_2_, ethanol and the short chain fatty acids, formiate, acetate, propionate, lactate and butyrate were found^14^ In contrast to ADM1, valeriate was missing, but formiate, ethanol and lactate may be considered as new metabolites in the model. However adding new metabolites increases the complexity of the ADM1, in particular when redundancy is increased in the model. Furthermore, the varying amounts of enzymes indicate that sugars are not degraded in certain stoichiometric ratios as assumed by the ADM1.

No transport proteins for propionate, butyrate and ethanol were found. These molecules were probably passively transported *via* diffusion^15, 16^. Formiate transporters were found abundantly, providing further evidence for the important role of this metabolite. Formiate in particular could serve as an underappreciated alternative to inter-species H_2_transfer in syntrophic interactions^17, 18^. Obviously, ethanol and the short chain fatty acids were further degraded into acetate, CO_2_ and H_2_by secondary fermenters. However, pathways for primary and secondary fermentation share the majority of enzymes and could not be differentiated adequately. The utilization of amino acids is more complex following individual metabolic pathways. Different abundances of amino acid metabolizing pathways in BGPs were discussed to be related to different availability in the feed substrates^4^. The degradation of most amino acids was linked to the transamination of oxoglutarate to glutamate. In accordance to ADM1, glutamate conversion releasing NH_3_ is a key function for degradation of amino acids, except for glycine degradation. Enzymes for the reduction reaction from glycine to acetyl-phosphate and an oxidation reaction from glycine to formate^3^ were also found highly correlated in BGPs. Both reactions could be coupled similar to a Stickland fermentation^19^. The assumptions going into the ADM1, namely that amino acid degradation produces all short chain fatty acids and H_2_, is confirmed by our findings. Overall, the metaprotein profiles clearly showed that according to ADM1 the majority of substrates were converted into formate, H_2_, acetate and CO_2_. Finally, methanogens use these compounds for CH_4_ production.

### Acetogenesis & methanogenesis

The analysis of archaeal metaproteins revealed that hydrogenotrophic methanogenesis was universal for all BGPs, with some BGPs strictly following this pathway. Acetoclastic methanogenesis was found predominantly in five of eleven BGPs. Methylotrophic methanogenesis was also detected^20^, and represented up to 20% of metaproteins of the CH_4_ producing pathways in BGP_04. Methylamines and methanol are the necessary precursors for methylotrophic methanogenesis using cholines.

As already discussed in the main manuscript, two groups of BGPs were classified. Whereas the first group of BGPs with acetoclastic and hydrogenotrophic methanogenesis contained only trace amounts of the bacterial C1 metabolism, the second group of strictly hydrogenotrophic BGPs, showed high abundance of bacterial C1 metabolism allowing the interconversion of acetate to H_2_ and CO_2_. Due to the missing acetoclastic methanogenesis as sink for acetate, the most likely metabolic flow is SAO^21^. However, the assignment to one of these groups did not correlate to any process conditions. The distinction might be also influenced by a combination of parameters such as temperature, metabolite concentrations and the inoculum.

Detailed analysis of BGPs methanogenesis revealed two pathways related to methanogenesis that are not covered by ADM1, recently. Whereas the abundance of methylotrophic methanogenesis could be probably neglected, SAO seems to be a major process pathways in agricultural BGPs. Thus, it may be considered when adapting ADM1 to agricultural BGPs.

**Reference**

1. de Gonzalo G, Colpa DI, Habib MH, Fraaije MW. Bacterial enzymes involved in lignin degradation. Journal of Biotechnology 236, 110-119 (2016).

2. Porter AW, Young LY. Benzoyl-CoA, a universal biomarker for anaerobic degradation of aromatic compounds. Advances in Applied Microbiology 88, 167-203 (2014).

3. Andreesen JR. Glycine metabolism in anaerobes. Antonie Van Leeuwenhoek 66, 223-237 (1994).

4. Heyer R, Kohrs F, Reichl U, Benndorf D. Metaproteomics of complex microbial communities in biogas plants. Microbial Biotechnology 8, 749-763 (2015).

5. Kohrs F, et al. Sample prefractionation with liquid isoelectric focusing enables in depth microbial metaproteome analysis of mesophilic and thermophilic biogas plants. Anaerobe 29, 59-67 (2014).

6. Heyer R, et al. Metaproteome analysis of the microbial communities in agricultural biogas plants. New Biotechnology 30, 614-622 (2013).

7. Munk B, Bauer C, Gronauer A, Lebuhn M. Population dynamics of methanogens during acidification of biogas fermenters fed with maize silage. Engineering in Life Sciences 10, 496-508 (2010).

8. Maupin-Furlow JA, Ferry JG. Analysis of the CO dehydrogenase/acetyl-coenzyme A synthase operon of Methanosarcina thermophila. Journal of Bacteriology 178, 6849-6856 (1996).

9. Abram F, Gunnigle E, O'Flaherty V. Optimisation of protein extraction and 2-DE for metaproteomics of microbial communities from anaerobic wastewater treatment biofilms. Electrophoresis 30, 4149-4151 (2009).

10. Hagen LH, et al. Quantitative Metaproteomics Highlight the Metabolic Contributions of Uncultured Phylotypes in a Thermophilic Anaerobic Digester. Applied and Environmental Microbiology 83, (2017).

11. Hanreich A, et al. Metagenome and metaproteome analyses of microbial communities in mesophilic biogas-producing anaerobic batch fermentations indicate concerted plant carbohydrate degradation. Systematic and Applied Microbiology 36, 330-338 (2013).

12. Heyer R, et al. Proteotyping of biogas plant microbiomes separates biogas plants according to process temperature and reactor type. Biotechnology for Biofuels 9, 155 (2016).

13. Jia X, Xi BD, Li MX, Yang Y, Wang Y. Metaproteomics analysis of the functional insights into microbial communities of combined hydrogen and methane production by anaerobic fermentation from reed straw. Plos One 12, (2017).

14. Lü F, et al. Metaproteomics of cellulose methanisation under thermophilic conditions reveals a surprisingly high proteolytic activity. The ISME Journal 8, 88-102 (2014).

15. Abbott DA, Zelle RM, Pronk JT, van Maris AJA. Metabolic engineering of Saccharomyces cerevisiae for production of carboxylic acids: current status and challenges. Fems Yeast Research 9, 1123-1136 (2009).

16. Dashper SG, Reynolds EC. Lactic acid excretion by Streptococcus mutans. Microbiology-Uk 142, 33-39 (1996).

17. Dube CD, Guiot SR. Direct Interspecies Electron Transfer in Anaerobic Digestion: A Review. Advances in Biochemical Engineering / Biotechnology 151, 101-115 (2015).

18. Rotaru AE, et al. Interspecies Electron Transfer via Hydrogen and Formate Rather than Direct Electrical Connections in Cocultures of Pelobacter carbinolicus and Geobacter sulfurreducens. Applied and Environmental Microbiology 78, 7645-7651 (2012).

19. Nisman B. The Stickland reaction. Bacteriological Reviews 18, 16-42 (1954).

20. Ziganshin AM, Ziganshina EE, Kleinsteuber S, Nikolausz M. Comparative Analysis of Methanogenic Communities in Different Laboratory-Scale Anaerobic Digesters. Archaea, (2016).

21. Westerholm M, Moestedt J, Schnurer A. Biogas production through syntrophic acetate oxidation and deliberate operating strategies for improved digester performance. Applied Energy 179, 124-135 (2016).
