# Supplementary material for: Metaproteome analysis reveals that syntrophy, competition, and phage-host interaction shape microbial communities in biogas plants
Source: Microbiome. 2019 Apr 27;7:69. doi: 10.1186/s40168-019-0673-y (PMC6486700; doi:10.1186/s40168-019-0673-y)
Supplement: Supplementary file 7 — Table S5. Abundance of main microbial families, host families of phages as well as the abundance of microbial immune response as represented by CRISPR proteins. Identified microbial metaproteins, phage metaprotein and CRISPR metaproteins were grouped by their (host) families and their spectral counts are shown as averages with the associated standard deviation. In contrast to the calculation of the phage abundance in Fig. 2, Additional file 12 this calculation considers also metaproteins that were assigned on root level, only. These metaprotein were assigned to phages based on their function. The abundance of the microbial families was normalized to the total number of identified microbial spectra. For the abundance of phages metaproteins and CRISPR metaproteins the spectral counts were normalized to the spectral counts of the corresponding microbial families. For a better overview the table was divided in A.) Bacterial families, B.) Archaeal families, C.) Others and D.) Overall. The detailed assignment can be found in Additional file 6: Table S4. F: taxonomic family; P: phage; C: CRISPR proteins. Differences between the abundances of phages assigned to archaea and to bacteria were validated by student’s t-test, showing with a p-value <0.00442 larger amounts of phages assigned to bacteria. For further details for the creation of this table please refer to Additional file 6: Table S4. (PDF 785 kb) [file 40168_2019_673_MOESM7_ESM.pdf]

A. Bacterial families		Total sum of spectra	Average BGP_02	Average BGP_03	Average BGP_04	Average BGP_05a	Average BGP_05b	Average BGP_07	Average BGP_09	Average BGP_10	Average BGP_X1	Average BGP_X2	Average BGP_X3	Total amount of	
Bacillaceae	F	37541	381 ± 67	954 ± 159	1178 ± 95	1501 ± 247	1159 ± 368	882 ± 95	760 ± 109	875 ± 151	536 ± 95	893 ± 63	268 ± 144	%of all microorganisms	6.7%
	P	264	3 ± 3	3 ± 2	4 ± 2	19 ± 8	17 ± 10	8 ± 4	5 ± 3	1 ± 1	4 ± 3	1 ± 2	2 ± 3	%of phage proteins for this family	0.7%
	C	0	0 ± 0	0 ± 0	0 ± 0	0 ± 0	0 ± 0	0 ± 0	0 ± 0	0 ± 0	0 ± 0	0 ± 0	0 ± 0	%of CRISPR proteins for this family	0.0%
Enterobacteriaceae	F	19308	159 ± 12	543 ± 103	495 ± 125	587 ± 209	431 ± 139	594 ± 50	308 ± 45	350 ± 81	384 ± 105	373 ± 51	604 ± 751	%of all microorganisms	3.4%
	P	538	4 ± 1	2 ± 1	3 ± 1	17 ± 8	7 ± 5	4 ± 2	69 ± 17	4 ± 3	22 ± 4	2 ± 1	2 ± 2	%of phage proteins for this family	2.8%
	C	71	1 ± 1	0 ± 0	10 ± 3	1 ± 1	0 ± 1	0 ± 0	3 ± 1	0 ± 1	1 ± 1	2 ± 2	0 ± 0	%of CRISPR proteins for this family	0.4%
Thermoanaerobacteraceae	F	11565	692 ± 482	24 ± 9	108 ± 45	540 ± 155	373 ± 135	68 ± 12	348 ± 84	456 ± 180	47 ± 29	229 ± 36	8 ± 7	%of all microorganisms	2.1%
	P	0	0 ± 0	0 ± 0	0 ± 0	0 ± 0	0 ± 0	0 ± 0	0 ± 0	0 ± 0	0 ± 0	0 ± 0	0 ± 0	%of phage proteins for this family	0.0%
	C	0	0 ± 0	0 ± 0	0 ± 0	0 ± 0	0 ± 0	0 ± 0	0 ± 0	0 ± 0	0 ± 0	0 ± 0	0 ± 0	%of CRISPR proteins for this family	0.0%
Desulfovibrionaceae	F	9585	102 ± 32	474 ± 167	180 ± 54	94 ± 25	91 ± 34	481 ± 153	82 ± 17	304 ± 95	226 ± 95	138 ± 84	226 ± 230	%of all microorganisms	1.7%
	P	0	0 ± 0	0 ± 0	0 ± 0	0 ± 0	0 ± 0	0 ± 0	0 ± 0	0 ± 0	0 ± 0	0 ± 0	0 ± 0	%of phage proteins for this family	0.0%
	C	1	0 ± 0	0 ± 0	0 ± 0	0 ± 0	0 ± 0	0 ± 0	0 ± 0	0 ± 1	0 ± 0	0 ± 0	0 ± 0	%of CRISPR proteins for this family	0.0%
Thermotogaceae	F	8618	16 ± 4	24 ± 6	42 ± 10	1043 ± 153	917 ± 233	16 ± 5	18 ± 5	10 ± 4	22 ± 4	28 ± 17	19 ± 11	%of all microorganisms	1.5%
	P	0	0 ± 0	0 ± 0	0 ± 0	0 ± 0	0 ± 0	0 ± 0	0 ± 0	0 ± 0	0 ± 0	0 ± 0	0 ± 0	%of phage proteins for this family	0.0%
	C	0	0 ± 0	0 ± 0	0 ± 0	0 ± 0	0 ± 0	0 ± 0	0 ± 0	0 ± 0	0 ± 0	0 ± 0	0 ± 0	%of CRISPR proteins for this family	0.0%
Spirochaetaceae	F	7847	53 ± 23	82 ± 8	156 ± 17	104 ± 28	101 ± 11	347 ± 86	47 ± 5	765 ± 318	141 ± 22	131 ± 14	35 ± 42	%of all microorganisms	1.4%
	P	0	0 ± 0	0 ± 0	0 ± 0	0 ± 0	0 ± 0	0 ± 0	0 ± 0	0 ± 0	0 ± 0	0 ± 0	0 ± 0	%of phage proteins for this family	0.0%
	C	0	0 ± 0	0 ± 0	0 ± 0	0 ± 0	0 ± 0	0 ± 0	0 ± 0	0 ± 0	0 ± 0	0 ± 0	0 ± 0	%of CRISPR proteins for this family	0.0%
Rhizobiaceae	F	7406	43 ± 30	192 ± 41	325 ± 25	224 ± 78	209 ± 21	109 ± 25	127 ± 29	191 ± 38	205 ± 74	164 ± 33	62 ± 64	%of all microorganisms	1.3%
	P	0	0 ± 0	0 ± 0	0 ± 0	0 ± 0	0 ± 0	0 ± 0	0 ± 0	0 ± 0	0 ± 0	0 ± 0	0 ± 0	%of phage proteins for this family	0.0%
	C	0	0 ± 0	0 ± 0	0 ± 0	0 ± 0	0 ± 0	0 ± 0	0 ± 0	0 ± 0	0 ± 0	0 ± 0	0 ± 0	%of CRISPR proteins for this family	0.0%
Clostridiaceae	F	7279	75 ± 24	288 ± 21	212 ± 42	432 ± 65	333 ± 96	145 ± 32	46 ± 9	73 ± 2	72 ± 40	111 ± 14	33 ± 22	%of all microorganisms	1.3%
	P	166	0 ± 0	0 ± 0	0 ± 0	31 ± 19	11 ± 4	0 ± 0	0 ± 0	0 ± 0	0 ± 0	0 ± 0	0 ± 0	%of phage proteins for this family	2.3%
	C	0	0 ± 0	0 ± 0	0 ± 0	0 ± 0	0 ± 0	0 ± 0	0 ± 0	0 ± 0	0 ± 0	0 ± 0	0 ± 0	%of CRISPR proteins for this family	0.0%
Streptococcaceae	F	3675	219 ± 163	130 ± 27	45 ± 13	88 ± 31	57 ± 15	68 ± 20	40 ± 22	64 ± 30	33 ± 16	159 ± 133	18 ± 12	%of all microorganisms	0.7%
	P	10	0 ± 0	0 ± 1	1 ± 1	0 ± 0	0 ± 1	1 ± 1	1 ± 1	0 ± 0	0 ± 0	0 ± 0	0 ± 1	%of phage proteins for this family	0.3%
	C	3	0 ± 0	0 ± 0	0 ± 0	1 ± 1	0 ± 0	0 ± 0	0 ± 0	0 ± 0	0 ± 0	0 ± 0	0 ± 0	%of CRISPR proteins for this family	0.1%
Mycobacteriaceae	F	2561	46 ± 12	143 ± 42	81 ± 13	97 ± 18	91 ± 45	48 ± 8	31 ± 9	19 ± 4	30 ± 10	33 ± 16	24 ± 15	%of all microorganisms	0.5%
	P	27	0 ± 1	0 ± 0	0 ± 0	1 ± 1	1 ± 1	1 ± 1	1 ± 1	0 ± 0	1 ± 1	3 ± 3	0 ± 1	%of phage proteins for this family	1.1%
	C	59	0 ± 0	6 ± 4	2 ± 1	0 ± 0	0 ± 0	5 ± 3	0 ± 0	0 ± 1	0 ± 0	1 ± 2	1 ± 1	%of CRISPR proteins for this family	2.3%
Enterococcaceae	F	2512	2 ± 2	13 ± 4	215 ± 54	48 ± 25	40 ± 13	53 ± 17	108 ± 21	16 ± 10	66 ± 12	58 ± 24	10 ± 9	%of all microorganisms	0.4%
	P	3	0 ± 0	0 ± 0	0 ± 0	0 ± 0	0 ± 1	0 ± 0	0 ± 0	0 ± 0	0 ± 0	0 ± 0	1 ± 1	%of phage proteins for this family	0.1%
	C	0	0 ± 0	0 ± 0	0 ± 0	0 ± 0	0 ± 0	0 ± 0	0 ± 0	0 ± 0	0 ± 0	0 ± 0	0 ± 0	%of CRISPR proteins for this family	0.0%
Pseudomonadaceae	F	1875	14 ± 3	49 ± 12	57 ± 9	35 ± 3	37 ± 6	86 ± 5	42 ± 36	59 ± 20	51 ± 21	31 ± 9	9 ± 5	%of all microorganisms	0.3%
	P	283	1 ± 1	1 ± 1	1 ± 1	39 ± 15	28 ± 16	2 ± 2	0 ± 0	0 ± 0	0 ± 1	0 ± 1	0 ± 0	%of phage proteins for this family	15.1%
	C	0	0 ± 0	0 ± 0	0 ± 0	0 ± 0	0 ± 0	0 ± 0	0 ± 0	0 ± 0	0 ± 0	0 ± 0	0 ± 0	%of CRISPR proteins for this family	0.0%
Vibrionaceae	F	1649	6 ± 2	40 ± 18	33 ± 6	123 ± 24	92 ± 32	27 ± 4	5 ± 2	9 ± 1	14 ± 7	25 ± 14	40 ± 38	%of all microorganisms	0.3%
	P	36	0 ± 0	3 ± 2	1 ± 1	0 ± 0	0 ± 0	0 ± 0	0 ± 0	0 ± 0	0 ± 0	2 ± 2	4 ± 2	%of phage proteins for this family	2.2%
	C	0	0 ± 0	0 ± 0	0 ± 0	0 ± 0	0 ± 0	0 ± 0	0 ± 0	0 ± 0	0 ± 0	0 ± 0	0 ± 0	%of CRISPR proteins for this family	0.0%
Pasteurellaceae	F	1347	21 ± 5	51 ± 15	39 ± 9	27 ± 14	38 ± 15	65 ± 24	8 ± 2	28 ± 10	14 ± 8	23 ± 8	24 ± 24	%of all microorganisms	0.2%
	P	8	1 ± 1	1 ± 1	0 ± 0	0 ± 0	0 ± 0	0 ± 0	0 ± 0	0 ± 0	0 ± 0	0 ± 0	0 ± 1	%of phage proteins for this family	0.6%
	C	27	0 ± 0	5 ± 4	2 ± 2	0 ± 0	0 ± 0	0 ± 0	0 ± 0	0 ± 0	0 ± 0	0 ± 0	0 ± 1	%of CRISPR proteins for this family	2.0%
Streptomycetaceae	F	1000	10 ± 7	54 ± 1	30 ± 8	26 ± 11	35 ± 10	26 ± 8	9 ± 1	20 ± 10	21 ± 3	15 ± 5	5 ± 3	%of all microorganisms	0.2%
	P	10	0 ± 0	0 ± 0	0 ± 0	2 ± 2	1 ± 1	0 ± 0	0 ± 0	0 ± 0	0 ± 0	0 ± 1	0 ± 0	%of phage proteins for this family	1.0%
	C	0	0 ± 0	0 ± 0	0 ± 0	0 ± 0	0 ± 0	0 ± 0	0 ± 0	0 ± 0	0 ± 0	0 ± 0	0 ± 0	%of CRISPR proteins for this family	0.0%
Acholeplasmataceae	F	894	18 ± 2	27 ± 9	21 ± 1	35 ± 20	21 ± 6	18 ± 3	24 ± 3	9 ± 2	19 ± 3	20 ± 14	11 ± 6	%of all microorganisms	0.2%
	P	89	0 ± 0	0 ± 0	0 ± 0	7 ± 2	7 ± 3	5 ± 3	4 ± 2	0 ± 0	0 ± 0	0 ± 0	0 ± 0	%of phage proteins for this family	10.0%
	C	0	0 ± 0	0 ± 0	0 ± 0	0 ± 0	0 ± 0	0 ± 0	0 ± 0	0 ± 0	0 ± 0	0 ± 0	0 ± 0	%of CRISPR proteins for this family	0.0%
Chlamydiaceae	F	529	3 ± 2	20 ± 8	7 ± 1	39 ± 6	28 ± 3	6 ± 5	5 ± 2	1 ± 1	8 ± 6	11 ± 12	4 ± 2	%of all microorganisms	0.1%
	P	47	0 ± 0	0 ± 0	0 ± 0	0 ± 0	0 ± 0	0 ± 1	0 ± 0	10 ± 4	2 ± 1	0 ± 0	0 ± 0	%of phage proteins for this family	8.9%
	C	0	0 ± 0	0 ± 0	0 ± 0	0 ± 0	0 ± 0	0 ± 0	0 ± 0	0 ± 0	0 ± 0	0 ± 0	0 ± 0	%of CRISPR proteins for this family	0.0%
Burkholderiaceae	F	436	1 ± 1	36 ± 9	18 ± 9	21 ± 9	12 ± 4	5 ± 3	1 ± 1	2 ± 1	4 ± 1	6 ± 5	4 ± 2	%of all microorganisms	0.1%
	P	9	1 ± 3	0 ± 0	0 ± 0	1 ± 1	0 ± 1	0 ± 0	0 ± 0	0 ± 0	0 ± 0	0 ± 1	0 ± 0	%of phage proteins for this family	2.1%
	C	0	0 ± 0	0 ± 0	0 ± 0	0 ± 0	0 ± 0	0 ± 0	0 ± 0	0 ± 0	0 ± 0	0 ± 0	0 ± 0	%of CRISPR proteins for this family	0.0%
Neisseriaceae	F	323	5 ± 6	21 ± 3	3 ± 2	5 ± 3	5 ± 5	9 ± 3	1 ± 1	1 ± 1	1 ± 1	18 ± 19	13 ± 12	%of all microorganisms	0.1%
	P	0	0 ± 0	0 ± 0	0 ± 0	0 ± 0	0 ± 0	0 ± 0	0 ± 0	0 ± 0	0 ± 0	0 ± 0	0 ± 0	%of phage proteins for this family	0.0%
	C	3	0 ± 0	1 ± 1	0 ± 0	0 ± 1	0 ± 0	0 ± 0	0 ± 0	0 ± 0	0 ± 0	0 ± 0	0 ± 0	%of CRISPR proteins for this family	0.9%
Chlorobiaceae	F	298	2 ± 2	16 ± 9	15 ± 2	9 ± 3	8 ± 2	6 ± 4	4 ± 1	3 ± 2	5 ± 1	6 ± 1	1 ± 1	%of all microorganisms	0.1%
	P	0	0 ± 0	0 ± 0	0 ± 0	0 ± 0	0 ± 0	0 ± 0	0 ± 0	0 ± 0	0 ± 0	0 ± 0	0 ± 0	%of phage proteins for this family	0.0%
	C	8	0 ± 0	2 ± 2	0 ± 1	0 ± 0	0 ± 0	0 ± 0	0 ± 0	0 ± 0	0 ± 0	0 ± 0	0 ± 0	%of CRISPR proteins for this family	2.7%

Thermaceae	F	237	1 ± 1	3 ± 3	7 ± 1	19 ± 7	15 ± 9	2 ± 2	3 ± 2	2 ± 2	4 ± 1	3 ± 2	2 ± 3	%of all microorganisms	0.0%
	P	0	0 ± 0	0 ± 0	0 ± 0	0 ± 0	0 ± 0	0 ± 0	0 ± 0	0 ± 0	0 ± 0	0 ± 0	0 ± 0	%of phage proteins for this family	0.0%
	C	4	0 ± 1	0 ± 0	1 ± 1	0 ± 0	0 ± 0	0 ± 0	0 ± 0	0 ± 0	0 ± 0	0 ± 0	0 ± 0	%of CRISPR proteins for this family	1.7%
Listeriaceae	F	217	1 ± 1	11 ± 7	5 ± 2	5 ± 4	1 ± 1	5 ± 3	11 ± 5	8 ± 4	3 ± 1	7 ± 5	1 ± 2	%of all microorganisms	0.0%
	P	2	0 ± 1	0 ± 0	0 ± 0	0 ± 0	0 ± 0	0 ± 0	0 ± 1	0 ± 0	0 ± 0	0 ± 0	0 ± 0	%of phage proteins for this family	0.9%
	C	0	0 ± 0	0 ± 0	0 ± 0	0 ± 0	0 ± 0	0 ± 0	0 ± 0	0 ± 0	0 ± 0	0 ± 0	0 ± 0	%of CRISPR proteins for this family	0.0%
Geobacteraceae	F	141	9 ± 11	6 ± 5	1 ± 1	5 ± 6	3 ± 1	1 ± 1	5 ± 4	1 ± 1	1 ± 1	3 ± 3	2 ± 2	%of all microorganisms	0.0%
	P	0	0 ± 0	0 ± 0	0 ± 0	0 ± 0	0 ± 0	0 ± 0	0 ± 0	0 ± 0	0 ± 0	0 ± 0	0 ± 0	%of phage proteins for this family	0.0%
	C	1	0 ± 0	0 ± 0	0 ± 0	0 ± 1	0 ± 0	0 ± 0	0 ± 0	0 ± 0	0 ± 0	0 ± 0	0 ± 0	%of CRISPR proteins for this family	0.7%
Rhodospirillaceae	F	72	1 ± 1	7 ± 5	2 ± 3	2 ± 3	1 ± 1	2 ± 3	1 ± 1	0 ± 0	1 ± 1	1 ± 1	1 ± 1	%of all microorganisms	0.0%
	P	0	0 ± 0	0 ± 0	0 ± 0	0 ± 0	0 ± 0	0 ± 0	0 ± 0	0 ± 0	0 ± 0	0 ± 0	0 ± 0	%of phage proteins for this family	0.0%
	C	7	0 ± 0	0 ± 0	0 ± 1	0 ± 0	0 ± 0	2 ± 2	0 ± 0	0 ± 0	0 ± 0	0 ± 0	0 ± 0	%of CRISPR proteins for this family	9.7%
Bdellovibrionaceae	F	12	0 ± 0	0 ± 1	0 ± 0	0 ± 1	1 ± 1	0 ± 0	0 ± 1	0 ± 1	1 ± 1	0 ± 1	0 ± 0	%of all microorganisms	0.0%
	P	3	1 ± 1	0 ± 0	0 ± 0	0 ± 0	0 ± 0	0 ± 0	0 ± 0	0 ± 0	0 ± 0	0 ± 0	0 ± 0	%of phage proteins for this family	25.0%
	C	0	0 ± 0	0 ± 0	0 ± 0	0 ± 0	0 ± 0	0 ± 0	0 ± 0	0 ± 0	0 ± 0	0 ± 0	0 ± 0	%of CRISPR proteins for this family	0.0%
Halomonadaceae	F	7	0 ± 1	0 ± 0	0 ± 0	1 ± 1	0 ± 1	1 ± 1	0 ± 0	0 ± 0	0 ± 0	0 ± 0	0 ± 0	%of all microorganisms	0.0%
	P	74	0 ± 1	0 ± 0	0 ± 0	9 ± 7	9 ± 8	0 ± 0	0 ± 0	0 ± 1	0 ± 0	0 ± 1	0 ± 0	%of phage proteins for this family	1057.1%
	C	0	0 ± 0	0 ± 0	0 ± 0	0 ± 0	0 ± 0	0 ± 0	0 ± 0	0 ± 0	0 ± 0	0 ± 0	0 ± 0	%of CRISPR proteins for this family	0.0%
Spiroplasmataceae	F	0	0 ± 0	0 ± 0	0 ± 0	0 ± 0	0 ± 0	0 ± 0	0 ± 0	0 ± 0	0 ± 0	0 ± 0	0 ± 0	%of all microorganisms	0.0%
	P	1	0 ± 0	0 ± 0	0 ± 0	0 ± 1	0 ± 0	0 ± 0	0 ± 0	0 ± 0	0 ± 0	0 ± 0	0 ± 0	%of phage proteins for this family	0.0%
	C	0	0 ± 0	0 ± 0	0 ± 0	0 ± 0	0 ± 0	0 ± 0	0 ± 0	0 ± 0	0 ± 0	0 ± 0	0 ± 0	%of CRISPR proteins for this family	0.0%
Sum of considered bacteria	F	126934	1879 ± 895	3206 ± 692	3272 ± 548	5106 ± 1150	4094 ± 1206	3067 ± 569	2033 ± 414	3265 ± 968	1905 ± 558	2485 ± 582	1422 ± 1408	%of all microorganisms	22.6%
	P	1570	11 ± 10	10 ± 6	9 ± 6	124 ± 62	80 ± 48	20 ± 12	79 ± 23	15 ± 8	28 ± 9	9 ± 10	9 ± 9	%of phage proteins for this family	1.2%
	C	184	1 ± 1	14 ± 9	14 ± 8	2 ± 3	0 ± 1	7 ± 5	3 ± 1	1 ± 2	1 ± 1	3 ± 4	1 ± 2	%of CRISPR proteins for this family	0.1%

**B. Archaeal families**		**Total sum of spectra**	**Average BGP_02**	**Average BGP_03**	**Average BGP_04**	**Average BGP_05a**	**Average BGP_05b**	**Average BGP_07**	**Average BGP_09**	**Average BGP_10**	**Average BGP_X1**	**Average BGP_X2**	**Average BGP_X3**	**Total amount of**	
Methanosarcinaceae	F	22754	359 ± 95	1722 ± 395	1030 ± 438	227 ± 25	218 ± 36	366 ± 26	411 ± 53	379 ± 114	398 ± 81	498 ± 217	81 ± 82	%of all microorganisms	4.0%
	P	0	0 ± 0	0 ± 0	0 ± 0	0 ± 0	0 ± 0	0 ± 0	0 ± 0	0 ± 0	0 ± 0	0 ± 0	0 ± 0	%of phage proteins for this family	0.0%
	C	0	0 ± 0	0 ± 0	0 ± 0	0 ± 0	0 ± 0	0 ± 0	0 ± 0	0 ± 0	0 ± 0	0 ± 0	0 ± 0	%of CRISPR proteins for this family	0.0%
Methanocaldococcaceae	F	14127	236 ± 194	673 ± 28	475 ± 94	367 ± 82	284 ± 96	270 ± 48	217 ± 32	322 ± 49	183 ± 71	374 ± 213	132 ± 105	%of all microorganisms	2.5%
	P	0	0 ± 0	0 ± 0	0 ± 0	0 ± 0	0 ± 0	0 ± 0	0 ± 0	0 ± 0	0 ± 0	0 ± 0	0 ± 0	%of phage proteins for this family	0.0%
	C	263	1 ± 1	1 ± 1	1 ± 1	35 ± 13	15 ± 6	0 ± 1	0 ± 0	13 ± 4	0 ± 0	1 ± 1	0 ± 1	%of CRISPR proteins for this family	1.9%
Archaeoglobaceae	F	5601	141 ± 99	195 ± 56	147 ± 35	171 ± 20	193 ± 10	64 ± 28	143 ± 31	96 ± 22	62 ± 21	116 ± 54	72 ± 85	%of all microorganisms	1.0%
	P	0	0 ± 0	0 ± 0	0 ± 0	0 ± 0	0 ± 0	0 ± 0	0 ± 0	0 ± 0	0 ± 0	0 ± 0	0 ± 0	%of phage proteins for this family	0.0%
	C	18	0 ± 0	0 ± 0	5 ± 2	0 ± 0	0 ± 0	0 ± 0	0 ± 0	0 ± 0	0 ± 0	0 ± 0	0 ± 0	%of CRISPR proteins for this family	0.3%
Methanomicrobiaceae	F	4637	22 ± 12	98 ± 20	84 ± 17	182 ± 27	131 ± 58	26 ± 5	203 ± 51	79 ± 8	112 ± 21	223 ± 105	1 ± 1	%of all microorganisms	0.8%
	P	0	0 ± 0	0 ± 0	0 ± 0	0 ± 0	0 ± 0	0 ± 0	0 ± 0	0 ± 0	0 ± 0	0 ± 0	0 ± 0	%of phage proteins for this family	0.0%
	C	0	0 ± 0	0 ± 0	0 ± 0	0 ± 0	0 ± 0	0 ± 0	0 ± 0	0 ± 0	0 ± 0	0 ± 0	0 ± 0	%of CRISPR proteins for this family	0.0%
Methanobacteriaceae	F	2891	88 ± 86	119 ± 38	42 ± 12	64 ± 21	51 ± 28	39 ± 7	57 ± 32	62 ± 22	23 ± 1	63 ± 48	117 ± 116	%of all microorganisms	0.5%
	P	0	0 ± 0	0 ± 0	0 ± 0	0 ± 0	0 ± 0	0 ± 0	0 ± 0	0 ± 0	0 ± 0	0 ± 0	0 ± 0	%of phage proteins for this family	0.0%
	C	0	0 ± 0	0 ± 0	0 ± 0	0 ± 0	0 ± 0	0 ± 0	0 ± 0	0 ± 0	0 ± 0	0 ± 0	0 ± 0	%of CRISPR proteins for this family	0.0%
Methanococcaceae	F	2162	44 ± 34	103 ± 23	40 ± 12	74 ± 23	67 ± 15	10 ± 2	66 ± 19	31 ± 9	20 ± 3	84 ± 87	2 ± 2	%of all microorganisms	0.4%
	P	0	0 ± 0	0 ± 0	0 ± 0	0 ± 0	0 ± 0	0 ± 0	0 ± 0	0 ± 0	0 ± 0	0 ± 0	0 ± 0	%of phage proteins for this family	0.0%
	C	0	0 ± 0	0 ± 0	0 ± 0	0 ± 0	0 ± 0	0 ± 0	0 ± 0	0 ± 0	0 ± 0	0 ± 0	0 ± 0	%of CRISPR proteins for this family	0.0%
Thermococcaceae	F	1945	12 ± 8	37 ± 9	35 ± 14	127 ± 17	112 ± 39	10 ± 5	46 ± 3	13 ± 4	59 ± 6	22 ± 13	16 ± 17	%of all microorganisms	0.3%
	P	0	0 ± 0	0 ± 0	0 ± 0	0 ± 0	0 ± 0	0 ± 0	0 ± 0	0 ± 0	0 ± 0	0 ± 0	0 ± 0	%of phage proteins for this family	0.0%
	C	132	0 ± 0	1 ± 1	9 ± 7	13 ± 7	8 ± 5	0 ± 0	2 ± 1	0 ± 0	1 ± 1	0 ± 1	0 ± 0	%of CRISPR proteins for this family	6.8%
Halobacteriaceae	F	588	5 ± 3	20 ± 9	11 ± 7	25 ± 19	23 ± 13	2 ± 3	21 ± 15	3 ± 2	3 ± 1	32 ± 33	2 ± 3	%of all microorganisms	0.1%
	P	2	0 ± 0	0 ± 0	0 ± 0	0 ± 1	0 ± 0	0 ± 0	0 ± 0	0 ± 0	0 ± 0	0 ± 0	0 ± 1	%of phage proteins for this family	0.3%
	C	43	0 ± 1	0 ± 0	0 ± 0	4 ± 2	5 ± 1	0 ± 0	0 ± 0	2 ± 2	0 ± 0	1 ± 1	0 ± 0	%of CRISPR proteins for this family	7.3%
Sulfolobaceae	F	339	8 ± 3	9 ± 2	12 ± 3	11 ± 2	11 ± 7	6 ± 3	4 ± 1	6 ± 4	10 ± 2	7 ± 1	2 ± 1	%of all microorganisms	0.1%
	P	2	0 ± 0	0 ± 0	0 ± 1	0 ± 0	0 ± 0	0 ± 0	0 ± 0	0 ± 0	0 ± 0	0 ± 0	0 ± 1	%of phage proteins for this family	0.6%
	C	1	0 ± 0	0 ± 0	0 ± 1	0 ± 0	0 ± 0	0 ± 0	0 ± 0	0 ± 0	0 ± 0	0 ± 0	0 ± 0	%of CRISPR proteins for this family	0.3%
Thermoproteaceae	F	80	0 ± 1	2 ± 1	2 ± 1	5 ± 1	3 ± 2	3 ± 2	1 ± 1	0 ± 0	1 ± 1	2 ± 2	3 ± 2	%of all microorganisms	0.0%
	P	0	0 ± 0	0 ± 0	0 ± 0	0 ± 0	0 ± 0	0 ± 0	0 ± 0	0 ± 0	0 ± 0	0 ± 0	0 ± 0	%of phage proteins for this family	0.0%
	C	1	0 ± 0	0 ± 0	0 ± 0	0 ± 0	0 ± 0	0 ± 0	0 ± 0	0 ± 0	0 ± 1	0 ± 0	0 ± 0	%of CRISPR proteins for this family	1.3%
Haloarculaceae	F	0	0 ± 0	0 ± 0	0 ± 0	0 ± 0	0 ± 0	0 ± 0	0 ± 0	0 ± 0	0 ± 0	0 ± 0	0 ± 0	%of all microorganisms	0.0%
	P	63	0 ± 0	0 ± 0	1 ± 1	0 ± 0	0 ± 0	0 ± 0	2 ± 1	1 ± 1	10 ± 8	1 ± 1	2 ± 3	%of phage proteins for this family	0.0%
	C	0	0 ± 0	0 ± 0	0 ± 0	0 ± 0	0 ± 0	0 ± 0	0 ± 0	0 ± 0	0 ± 0	0 ± 0	0 ± 0	%of CRISPR proteins for this family	0.0%
Sum of considered archaea	F	55124	915 ± 536	2977 ± 580	1876.3 ± 633	1251.3 ± 237	1091.8 ± 305	794.5 ± 129	1168.3 ± 238	990.75 ± 234	870.25 ± 207	1420 ± 773	426 ± 414	%of all microorganisms	9.8%
	P	67	0 ± 0	0 ± 0	1.25 ± 1.3	0.25 ± 0.5	0 ± 0	0 ± 0	1.75 ± 0.5	0.75 ± 1	9.75 ± 8.1	1 ± 0.8	2 ± 4	%of phage proteins for this family	0.1%
	C	458	1 ± 1.5	1.75 ± 1.8	14.25 ± 9.4	51.25 ± 22	27.5 ± 12	0.25 ± 0.5	1.5 ± 1	14.5 ± 6.3	0.75 ± 1.1	1.5 ± 2	0.25 ± 0.5	%of CRISPR proteins for this family	0.8%

C. Others		Total sum of spectra	Average BGP_02	Average BGP_03	Average BGP_04	Average BGP_05a	Average BGP_05b	Average BGP_07	Average BGP_09	Average BGP_10	Average BGP_X1	Average BGP_X2	Average BGP_X3	Total amount of	
Unassigned	F	348885	6845 ± 1273	11948 ± 1753	8775 ± 1383	13245 ± 2432	10335 ± 2670	7455 ± 366	5947 ± 400	5942 ± 1002	5816 ± 1395	7253 ± 1418	3662 2406	%of all microorganisms	62.0%
	P	7703	48 ± 20	115 ± 12	156 ± 74	627 ± 178	470 ± 187	110 ± 7	76 ± 18	49 ± 8	190 ± 149	70 ± 18	15 10	%of phage proteins for this family	2.2%
	C	2611	14 ± 7	151 ± 55	71 ± 50	205 ± 22	173 ± 44	4 ± 2	3 ± 2	15 ± 9	2 ± 2	12 ± 15	3 5	%of CRISPR proteins for this family	0.7%
Other not considered microbial families	F	31447	477 ± 148	1139 ± 336	731 ± 73	1062 ± 225	798 ± 253	836 ± 37	591 ± 75	501 ± 31	546 ± 44	762 ± 171	420 252	%of all microorganisms	5.6%
	P	0	0 ± 0	0 ± 0	0 ± 0	0 ± 0	0 ± 0	0 ± 0	0 ± 0	0 ± 0	0 ± 0	0 ± 0	0 0	%of phage proteins for this family	0.0%
	C	0	0 ± 0	0 ± 0	0 ± 0	0 ± 0	0 ± 0	0 ± 0	0 ± 0	0 ± 0	0 ± 0	0 ± 0	0 0	%of CRISPR proteins for this family	0.0%

D. Overall		Total sum of spectra	Average BGP_02	Average BGP_03	Average BGP_04	Average BGP_05a	Average BGP_05b	Average BGP_07	Average BGP_09	Average BGP_10	Average BGP_X1	Average BGP_X2	Average BGP_X3	Total amount of	
Total microbial spectra	F	562390	10116 ± 2228	19270 ± 3006	14655 ± 2402	20664 ± 3631	16319 ± 4223	12151 ± 609	9739 ± 617	10698 ± 1986	9137 ± 2078	11920 ± 2436	5930 ± 3638	%of all microorganisms	100.0%
	P	9340	59 ± 25	125 ± 11	166 ± 70	751 ± 200	550 ± 191	130 ± 7	157 ± 10	65 ± 12	228 ± 149	80 ± 20	26 ± 15	%of phage proteins for this family	1.7%
	C	3253	16 ± 9	166 ± 63	100 ± 64	258 ± 22	201 ± 48	11 ± 4	8 ± 3	30 ± 10	4 ± 3	17 ± 17	4 ± 6	%of CRISPR proteins for this family	0.6%
