# Supplementary figures and images for: Metaproteome analysis reveals that syntrophy, competition, and phage-host interaction shape microbial communities in biogas plants
Source: Microbiome. 2019 Apr 27;7:69. doi: 10.1186/s40168-019-0673-y (PMC6486700; doi:10.1186/s40168-019-0673-y)

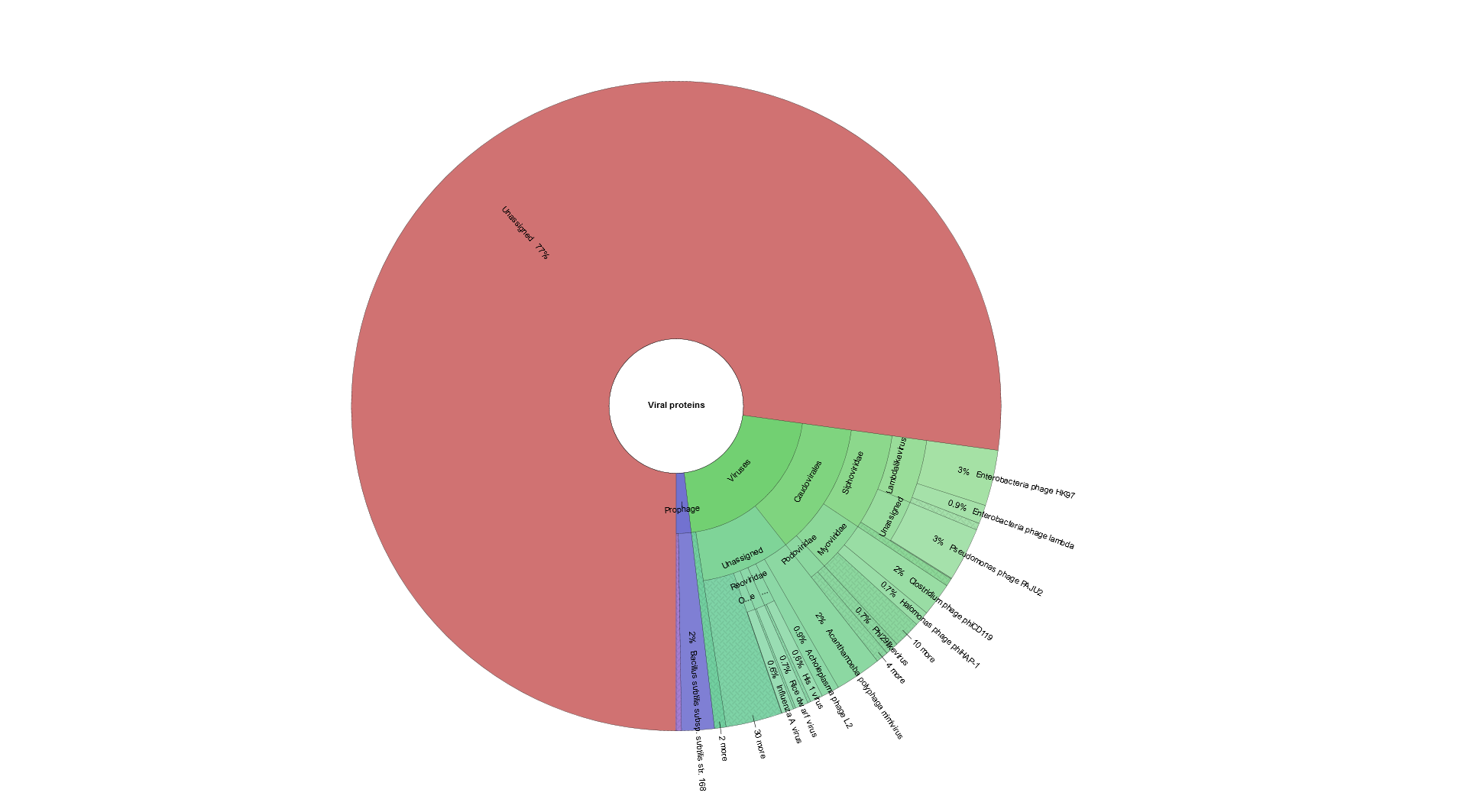

Supplement: Supplementary file 9 — Figure S2. Taxonomic profile of all identified viruses based on the number of identified viral spectra summed over all analyzed BGPs. (PNG 107 kb) [file 40168_2019_673_MOESM9_ESM.png]

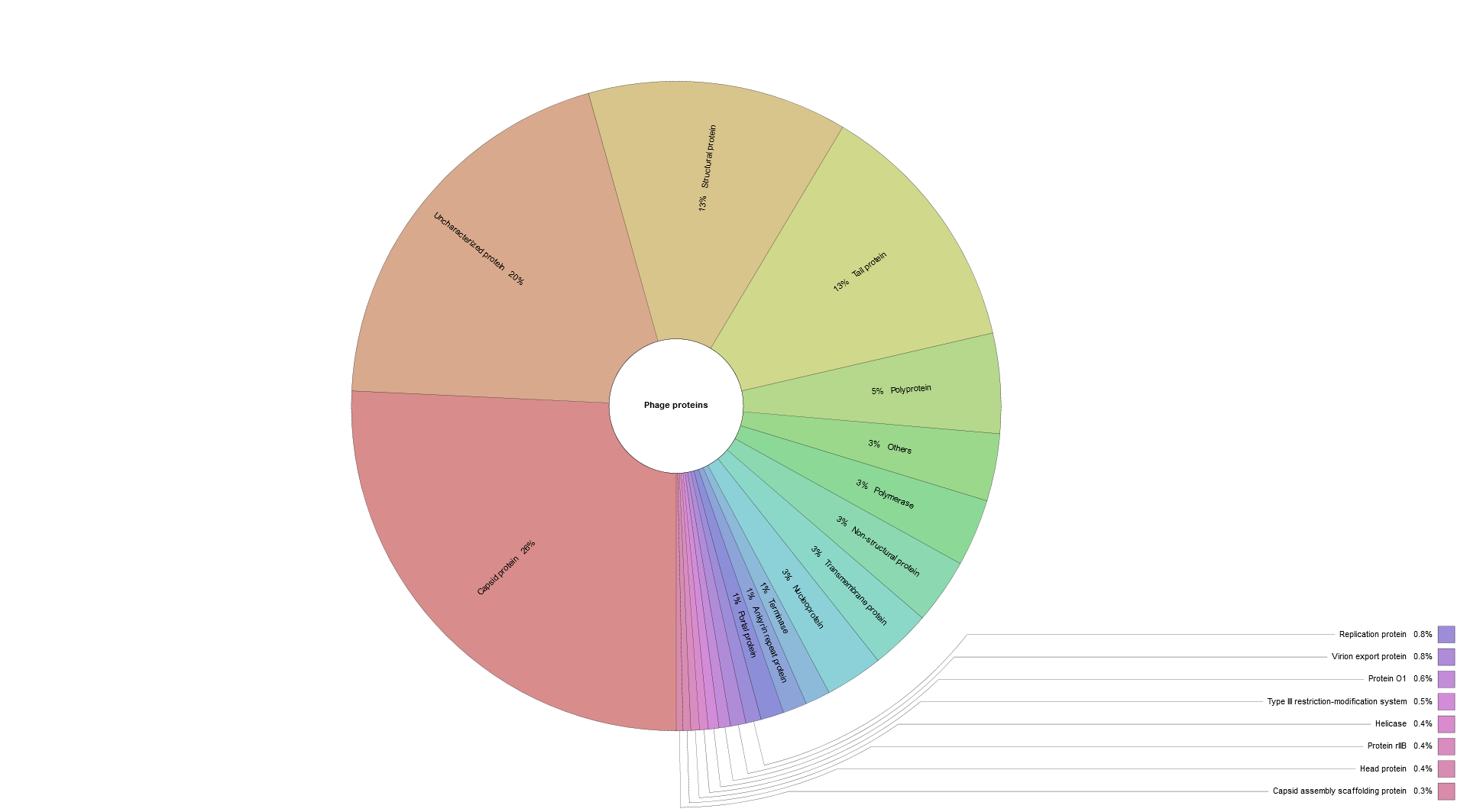

Supplement: Supplementary file 10 — Figure S3. Functional assignment of all identified phage spectra summed over all BGPs. (PNG 113 kb) [file 40168_2019_673_MOESM10_ESM.png]

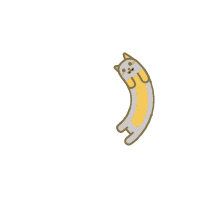

Supplement: Supplementary file 13 — An interactive version of Fig. 3. (ZIP 6150 kb) [file 40168_2019_673_MOESM13_ESM.zip › interactive_figure_3/bower_components/download.gif]
