# Supplementary material for: Metaproteome analysis reveals that syntrophy, competition, and phage-host interaction shape microbial communities in biogas plants
Source: Microbiome. 2019 Apr 27;7:69. doi: 10.1186/s40168-019-0673-y (PMC6486700; doi:10.1186/s40168-019-0673-y)
Supplement: Supplementary file 11 — Note 2. Estimation of the number of phage particles. (DOCX 21 kb) [file 40168_2019_673_MOESM11_ESM.docx]

Additional Note 2:

Estimation of the number of phage particles

The taxonomic assignment of all identified metaproteins revealed that based on the number of identified spectra about 0.4% of the microbial protein amount belonged to phages (manuscript Fig. 2). Since proteins are a main component of the microbial biomass this value correlates quite well with the phage biomass. In addition, the number of phage particles should be estimated based on this value. Therefore, the shape of microorganisms and phages were assumed spherical with a mean diameter of 1.0 µm for microorganisms and 100 nm for phages. Furthermore, the protein contents of phages and microorganisms were considered as equal.

Using equation 1 for sphere volumes the volume of a microorganisms is about 0.52 µm^3^ and the volume of phages is about 0.00052 µm^3^. This means that the phage volume is thousand times smaller than the volume of microorganisms. In consequence, a protein amount of 0.4% of the total microbial community corresponds to four times more phage particles than microorganisms.

$V= \frac{1}{6}\pi d^{3}$ (equation 1)

$$V_{microorganisms}= 0.52 {\mu m}^{3}$$

$$V_{phages}= 0.00052 {\mu m}^{3}$$
