# Supplementary material for: Metaproteome analysis reveals that syntrophy, competition, and phage-host interaction shape microbial communities in biogas plants
Source: Microbiome. 2019 Apr 27;7:69. doi: 10.1186/s40168-019-0673-y (PMC6486700; doi:10.1186/s40168-019-0673-y)
Supplement: Supplementary file 12 — An interactive version of Fig. 2. (HTML 408 kb) [file 40168_2019_673_MOESM12_ESM.html]

Javascript must be enabled to view this page.

magnitude

 562390

 122898

 1619

 1619

 246

 242

 228

 228

 7

 7

 5

 5

 2

 2

 4

 4

 4

 953

 953

 953

 953

 339

 339

 108

 108

 5

 5

 226

 3

 6

 19

 117

 81

 81

 1

 1

 1

 80

 2

 2

 75

 42

 2

 7

 1

 9

 14

 3

 3

 103744

 5601

 5601

 5601

 5601

 5600

 1

 588

 588

 588

 51

 51

 36

 36

 333

 1

 332

 95

 95

 2

 2

 13

 13

 20

 20

 38

 38

 2892

 2892

 2891

 44

 35

 9

 6

 3

 3

 4

 4

 2837

 29

 1180

 1628

 1

 1

 1

 16317

 16317

 14127

 14127

 14127

 2162

 2091

 3

 52

 14

 2016

 6

 71

 71

 28

 28

 28

 38813

 48

 48

 48

 48

 5407

 237

 237

 237

 4637

 4575

 4575

 62

 62

 104

 9

 9

 85

 85

 10

 10

 100

 100

 100

 329

 329

 329

 24666

 852

 852

 539

 92

 221

 22754

 6

 6

 20728

 164

 8966

 3672

 63

 7863

 2020

 2020

 1060

 1060

 1060

 8692

 8692

 8692

 8692

 24

 24

 24

 24

 24

 37429

 37429

 37429

 37429

 37429

 1945

 1945

 1945

 847

 198

 319

 97

 14

 219

 488

 488

 610

 25

 109

 436

 4

 36

 135

 135

 17

 17

 17

 117

 117

 66

 42

 9

 1

 1

 1

 1

 1

 1

 1

 1

 1

 3

 3

 3

 3

 3

 3

 17529

 17529

 17529

 17529

 17529

 17529

 2

 2

 1

 1

 1

 1

 1

 1

 1

 1

 437326

 13

 6

 6

 6

 6

 6

 7

 7

 7

 7

 7

 11004

 11004

 9714

 4

 4

 4

 357

 357

 357

 1

 1

 1

 1

 1

 1

 40

 40

 40

 103

 103

 1

 1

 8

 10

 54

 8

 9

 1

 2

 9

 387

 387

 372

 7

 6

 2

 1

 1

 1

 4

 4

 4

 26

 20

 20

 6

 6

 194

 169

 4

 1

 6

 1

 12

 99

 8

 3

 35

 1

 1

 21

 21

 2

 2

 1

 1

 19

 3

 3

 3

 1

 2

 13

 2

 11

 2561

 2561

 1

 6

 3

 40

 7

 26

 28

 8

 232

 1

 1705

 2

 10

 492

 1223

 2

 2

 1221

 9

 53

 1

 1

 10

 3

 1139

 5

 4

 4

 4

 23

 23

 23

 16

 16

 5

 10

 1

 25

 7

 1

 5

 1

 1

 1

 17

 17

 3722

 3705

 3705

 17

 17

 3

 3

 3

 1000

 1000

 1

 613

 72

 15

 1

 1

 18

 14

 6

 127

 1

 1

 37

 6

 1

 19

 4

 5

 1

 1

 1

 6

 3

 11

 1

 1

 28

 5

 591

 591

 591

 18

 24

 2

 38

 2

 133

 374

 421

 421

 421

 421

 278

 278

 278

 278

 1558

 1558

 1558

 1512

 1506

 1432

 74

 3

 3

 3

 3

 46

 42

 42

 4

 4

 4296

 2815

 2815

 1497

 1497

 558

 88

 3

 726

 102

 20

 989

 116

 116

 871

 871

 2

 2

 6

 6

 6

 323

 271

 271

 52

 52

 50

 50

 50

 47

 47

 3

 3

 1173

 1173

 1129

 84

 84

 62

 11

 32

 19

 419

 419

 11

 11

 553

 553

 44

 44

 44

 256

 236

 236

 230

 230

 6

 6

 20

 20

 12

 12

 8

 8

 2

 2

 2

 2

 2

 533

 533

 533

 529

 308

 16

 209

 11

 72

 7

 4

 1

 2

 214

 214

 4

 4

 4

 298

 298

 298

 298

 108

 4

 24

 80

 133

 4

 128

 1

 11

 11

 5

 3

 2

 6

 6

 35

 35

 237

 208

 199

 199

 10

 7

 3

 189

 7

 4

 178

 9

 9

 9

 9

 27

 27

 27

 27

 27

 2

 1

 1

 1

 1

 1

 1

 1

 1

 2568

 3

 3

 3

 3

 3

 2565

 478

 478

 478

 478

 1825

 1825

 2

 2

 12

 5

 4

 1

 2

 113

 113

 36

 36

 367

 21

 64

 13

 2

 1

 175

 10

 2

 7

 5

 1

 59

 7

 1285

 1285

 10

 10

 204

 204

 3

 1

 2

 169

 3

 165

 1

 32

 32

 13

 13

 4

 4

 2

 2

 7

 7

 45

 45

 45

 28

 7

 10

 2

 2

 2

 2

 2

 2

 381

 381

 144

 144

 144

 4

 4

 1

 135

 237

 237

 237

 4

 3

 1

 2

 227

 19

 19

 19

 19

 19

 14

 3

 2

 27

 1

 1

 1

 1

 1

 26

 26

 26

 26

 26

 2

 2

 2

 2

 2

 2

 113247

 58452

 43767

 5

 5

 4

 1

 37541

 7

 7

 35728

 63

 39

 42

 67

 194

 146

 4

 11

 503

 293

 27

 5

 5

 22

 1

 1

 4

 30

 1

 1

 1

 1

 6

 10

 6

 27793

 621

 7

 2

 5822

 344

 52

 4

 175

 90

 23

 28

 28

 58

 58

 1376

 1376

 217

 217

 55

 120

 3

 39

 142

 13

 1

 5

 7

 55

 6

 39

 10

 2

 2

 72

 1

 68

 2

 1

 69

 2

 2

 2

 2

 65

 65

 3680

 3668

 3668

 12

 5

 7

 2113

 2

 2

 29

 29

 2082

 1130

 198

 5

 16

 1

 4

 6

 1

 27

 1

 2

 43

 5

 21

 52

 432

 1

 2

 76

 3

 56

 6913

 2512

 2512

 5

 35

 27

 2445

 366

 311

 14

 16

 3

 2

 2

 1

 38

 3

 46

 4

 1

 181

 15

 15

 40

 40

 235

 140

 140

 20

 20

 75

 73

 2

 125

 125

 125

 3675

 1771

 84

 158

 28

 1407

 94

 65

 65

 1839

 905

 2

 17

 4

 5

 1

 46

 3

 1

 67

 571

 183

 7

 4

 8

 15

 7772

 7772

 7772

 7772

 30127

 12625

 7279

 430

 211

 139

 80

 6809

 2312

 268

 40

 123

 267

 3

 4

 636

 1012

 214

 281

 128

 46

 12

 1

 1

 1

 2

 9

 74

 20

 253

 1102

 40

 40

 32

 32

 32

 37

 37

 37

 2061

 2061

 1815

 239

 5

 2

 42

 42

 42

 7

 7

 7

 616

 47

 47

 145

 145

 133

 133

 222

 222

 69

 69

 438

 438

 142

 5

 5

 286

 1573

 1573

 1573

 274

 274

 3

 271

 266

 266

 266

 474

 474

 474

 474

 26

 26

 26

 26

 5096

 5096

 5096

 5096

 11906

 21

 21

 21

 11565

 482

 482

 157

 157

 6851

 6851

 3777

 3777

 298

 29

 1

 241

 19

 8

 320

 225

 13

 196

 6

 10

 95

 1

 94

 1554

 1554

 1554

 1554

 1554

 23114

 23114

 23114

 23114

 23114

 142

 142

 142

 141

 140

 1

 5

 134

 1

 1

 1

 1

 1

 8

 8

 8

 8

 8

 8

 4

 4

 4

 4

 4

 4

 43

 43

 43

 43

 43

 43

 84604

 10433

 126

 126

 124

 1

 123

 2

 2

 7

 7

 7

 7

 403

 403

 399

 399

 4

 4

 8567

 50

 50

 28

 20

 1

 1

 5

 5

 5

 63

 17

 1

 14

 1

 1

 9

 7

 2

 4

 4

 33

 33

 60

 48

 8

 4

 1

 35

 12

 12

 1

 1

 1

 518

 518

 388

 18

 1

 104

 3

 4

 26

 2

 2

 5

 5

 19

 19

 360

 360

 360

 7406

 162

 43

 1

 102

 11

 5

 14

 14

 31

 6

 19

 2

 3

 1

 7199

 876

 2

 6321

 78

 8

 8

 1

 1

 69

 68

 1

 282

 11

 9

 9

 2

 2

 271

 1

 1

 1

 1

 28

 27

 1

 127

 15

 112

 4

 4

 104

 104

 6

 6

 98

 26

 1

 1

 6

 6

 8

 5

 1

 2

 7

 7

 4

 4

 72

 2

 2

 8

 8

 62

 8

 54

 703

 31

 7

 2

 2

 3

 4

 3

 1

 13

 13

 7

 1

 6

 672

 3

 3

 669

 153

 20

 1

 135

 65

 217

 40

 1

 28

 1

 8

 247

 2

 2

 2

 245

 4

 4

 196

 15

 181

 5

 4

 1

 3

 3

 1

 1

 36

 2

 34

 2749

 2079

 335

 5

 5

 1

 1

 329

 42

 2

 15

 255

 3

 12

 436

 64

 2

 9

 1

 3

 4

 1

 23

 2

 2

 2

 6

 9

 134

 9

 117

 3

 2

 3

 1

 1

 2

 1

 1

 235

 59

 176

 78

 3

 1

 1

 1

 33

 33

 3

 3

 13

 13

 17

 1

 16

 4

 4

 4

 4

 1

 1

 14

 1

 1

 5

 5

 2

 2

 6

 6

 1216

 1214

 1214

 1

 1

 1

 1

 2

 2

 2

 2

 149

 149

 8

 8

 140

 140

 1

 1

 323

 323

 12

 12

 1

 1

 3

 3

 307

 16

 2

 284

 5

 68

 68

 17

 13

 4

 51

 51

 70

 70

 70

 70

 58

 58

 1

 1

 5

 5

 8

 8

 44

 44

 11687

 12

 12

 12

 12

 93

 82

 21

 21

 18

 18

 43

 43

 11

 11

 11

 9922

 337

 337

 335

 2

 9585

 9585

 12

 836

 8234

 1

 76

 425

 1

 278

 141

 141

 1

 1

 25

 6

 28

 5

 75

 129

 1

 1

 128

 22

 106

 8

 8

 8

 398

 1

 1

 1

 391

 71

 2

 9

 5

 55

 320

 1

 319

 6

 6

 6

 640

 640

 640

 640

 344

 340

 340

 340

 4

 4

 4

 1485

 1458

 148

 57

 57

 91

 4

 5

 5

 3

 1

 6

 61

 6

 1232

 734

 2

 1

 9

 717

 5

 317

 317

 181

 181

 78

 78

 78

 4

 4

 4

 4

 23

 23

 19

 19

 4

 4

 39734

 13

 13

 13

 13

 48

 48

 39

 1

 4

 3

 21

 4

 6

 9

 9

 533

 27

 19

 10

 9

 4

 4

 4

 4

 10

 10

 10

 185

 185

 8

 9

 167

 1

 7

 7

 7

 7

 5

 5

 2

 2

 297

 297

 245

 5

 1

 2

 13

 5

 1

 13

 5

 1

 2

 3

 1

 234

 234

 234

 234

 39

 13

 4

 4

 9

 9

 25

 10

 10

 13

 13

 2

 2

 1

 1

 1

 19308

 19308

 41

 5

 36

 14

 14

 2

 2

 22

 1

 16

 5

 7

 7

 43

 38

 5

 12

 3

 9

 45

 3

 29

 13

 9

 1

 5

 3

 8151

 8128

 1

 21

 1

 104

 27

 74

 3

 17

 2

 15

 55

 48

 6

 1

 10

 10

 12

 3

 8

 1

 9045

 9045

 1370

 47

 90

 43

 1

 1

 1

 1

 2

 1

 1170

 13

 108

 79

 29

 41

 2

 8

 20

 9

 2

 8

 8

 7

 7

 3

 3

 182

 12

 57

 4

 28

 81

 116

 34

 34

 34

 82

 11

 2

 9

 71

 71

 60

 60

 60

 60

 34

 18

 18

 18

 6

 6

 6

 7

 5

 5

 2

 2

 3

 3

 3

 1347

 1347

 30

 27

 3

 4

 4

 2

 2

 32

 32

 1094

 201

 879

 5

 8

 1

 4

 4

 168

 168

 13

 13

 2153

 276

 199

 100

 78

 11

 3

 7

 8

 4

 4

 12

 3

 4

 1

 3

 1

 57

 57

 1875

 5

 5

 37

 37

 1791

 789

 1

 7

 2

 5

 14

 4

 2

 441

 240

 2

 103

 22

 5

 154

 42

 42

 2

 2

 2

 13568

 13568

 13559

 13553

 2

 2

 2

 7

 7

 2

 2

 22

 20

 20

 1

 15

 2

 1

 1

 2

 2

 2

 1649

 1649

 15

 12

 3

 468

 2

 466

 18

 18

 1148

 409

 6

 284

 368

 8

 46

 2

 2

 3

 20

 610

 610

 43

 43

 7

 7

 474

 1

 1

 452

 4

 12

 4

 86

 86

 18516

 18516

 18516

 18516

 18483

 1

 6

 2

 1

 23

 199489

 199489

 199489

 199489

 199424

 199424

 64

 64

 1

 1

 8005

 8005

 8005

 113

 113

 113

 45

 45

 26

 2

 17

 7847

 943

 4

 905

 1

 3

 30

 15

 15

 1

 1

 6888

 3663

 136

 2

 2952

 135

 4

 4

 4

 4

 4

 4

 2219

 2219

 894

 894

 833

 833

 61

 31

 1

 17

 12

 5

 5

 5

 5

 1320

 1320

 1310

 3

 3

 71

 8

 157

 1

 16

 3

 90

 888

 1

 6

 63

 10

 3

 5

 2

 8618

 8618

 8618

 8618

 20

 20

 1

 1

 1768

 1768

 2306

 2306

 335

 333

 2

 4188

 24

 3657

 246

 234

 22

 5

 5

 2

 2

 2

 2

 2

 3

 3

 3

 3

 3

 2166

 2166

 2166

 1231

 377

 9

 9

 74

 74

 1

 1

 4

 3

 1

 166

 166

 1

 1

 17

 17

 49

 19

 24

 3

 1

 2

 36

 36

 2

 2

 16

 1

 1

 13

 1

 2

 2

 140

 1

 1

 2

 2

 65

 51

 1

 13

 56

 56

 2

 1

 1

 14

 14

 714

 27

 6

 12

 9

 404

 1

 30

 3

 277

 93

 10

 10

 2

 2

 271

 3

 1

 267

 33

 7

 7

 7

 25

 1

 1

 1

 1

 2

 2

 3

 3

 3

 3

 3

 1

 2

 4

 3

 1

 4

 1

 2

 1

 4

 1

 1

 2

 1

 1

 1

 7

 1

 1

 1

 6

 3

 3

 1

 1

 2

 2

 26

 26

 4

 2

 2

 16

 16

 6

 2

 3

 1

 53

 40

 1

 1

 1

 1

 31

 1

 30

 5

 5

 1

 1

 1

 1

 13

 13

 11

 2

 816

 3

 3

 1

 1

 1

 1

 1

 1

 1

 1

 1

 8

 8

 8

 4

 4

 1

 3

 1

 1

 1

 1

 1

 1

 2

 1

 1

 1

 1

 1

 1

 1

 2

 1

 1

 1

 1

 1

 1

 1

 4

 4

 4

 2

 2

 2

 4

 4

 4

 17

 16

 16

 1

 1

 15

 3

 3

 9

 9

 3

 3

 1

 1

 1

 1

 1

 1

 52

 3

 3

 47

 47

 2

 2

 231

 231

 231

 3

 3

 3

 62

 60

 60

 1

 1

 1

 1

 15

 13

 13

 2

 2

 1

 1

 1

 2

 2

 1

 1

 89

 89

 89

 15

 2

 1

 1

 13

 13

 44

 21

 21

 1

 1

 20

 20

 2

 2

 76

 1

 1

 5

 5

 65

 65

 5

 1

 3

 1

 24

 1

 1

 1

 1

 22

 1

 3

 13

 5

 1

 1

 1

 2

 2

 2

 1

 1

 1

 78

 2

 2

 1

 1

 63

 63

 2

 2

 10

 2

 7

 1

 51

 1

 1

 50

 1

 49
