# Supplementary material for: Metaproteome analysis reveals that syntrophy, competition, and phage-host interaction shape microbial communities in biogas plants
Source: Microbiome. 2019 Apr 27;7:69. doi: 10.1186/s40168-019-0673-y (PMC6486700; doi:10.1186/s40168-019-0673-y)
Supplement: Supplementary file 13 — An interactive version of Fig. 3. (ZIP 6150 kb) [file 40168_2019_673_MOESM13_ESM.zip › interactive_figure_3/bower_components/modernizr/media/Modernizr 2 Logo.pdf]

Modernizr
