# Supplementary material for: Metaproteome analysis reveals that syntrophy, competition, and phage-host interaction shape microbial communities in biogas plants
Source: Microbiome. 2019 Apr 27;7:69. doi: 10.1186/s40168-019-0673-y (PMC6486700; doi:10.1186/s40168-019-0673-y)
Supplement: Supplementary file 13 — An interactive version of Fig. 3. (ZIP 6150 kb) [file 40168_2019_673_MOESM13_ESM.zip › interactive_figure_3/index/index.html]

TaxFuncRel


# Taxonomy Function Relationship in metaproteomics

---

###### Taxonomy Function Relationship

##### Objects

Reset all filter

Search in the objects:

- {{ filter.name }}

  {{ filter.name }}


##### Filters

Filter every object

Add all filter

- {{ filter.name }}

  {{ filter.name }}

##### Data Source

Link to CSV Data:


load


###### {{ tooltip.sname }} to {{ tooltip.tname }} in relation with Impact {{tooltip.svalue}}/{{tooltip.stotal}} ({{(tooltip.svalue/tooltip.stotal)\*100}} %) and

###### {{ tooltip.tname }} to {{ tooltip.sname }} in relation with Impact {{tooltip.tvalue}}/{{tooltip.ttotal}} ({{(tooltip.tvalue/tooltip.ttotal)\*100}} %)

###### {{ tooltip.gname }} has a total of {{tooltip.gvalue}}


- Source delimited.io
- Data from MeatProteomeAnalyzer
